# Unique adaptations in sulfatases underpin colonic mucin degradation by *Akkermansia muciniphila*

**DOI:** 10.1038/s41564-026-02424-1

**Published:** 2026-07-27

**Authors:** Debajit Dey, Naba D. Salman, Charles W. E. Tomlinson, Chunsheng Jin, Grete Raba, Sadie R. Schaus, Marcus Nilsson, Zak McIver, Reyme Herman, Adam Simpkin, Matthew Davy, Daniel J. Rigden, Mirjam Czjzek, Dominic P. Byrne, Mihai Oltean, Alexander Case, Christoph G. Baumann, Gareth S. A. Wright, Sjoerd van der Post, Edwin A. Yates, Eric C. Martens, Lauren Davey, Ana S. Luis, Alan Cartmell

**Affiliations:** 1 Department of Biology, University of York, York, UK; 2 York Structural Biology Laboratory (YSBL), Department of Chemistry, University of York, York, UK; 3 York Biomedical Research Institute (YBRI), University of York, York, UK; 4 Institute of Biomedicine, Department of Medical Biochemistry and Cell Biology, University of Gothenburg, Gothenburg, Sweden; 5 Proteomics Core Facility at Sahlgrenska Academy, University of Gothenburg, Gothenburg, Sweden; 6 Department of Chemistry and Biotechnology, Tallinn University of Technology, Tallinn, Estonia; 7 Department of Microbiology and Immunology, University of Michigan, Ann Arbor, MI USA; 8 Department of Biochemistry, Cell and Systems Biology, Institute of Systems, Molecular and Integrative Biology, University of Liverpool, Liverpool, UK; 9 Integrative Biology of Marine Models, UMR 8227, Station Biologique de Roscoff, CNRS, Sorbonne Université, Univ Paris 06, Roscoff, France; 10 Department of Transplantation Surgery, Institute for Clinical Sciences, Sahlgrenska Academy, University of Gothenburg, Gothenburg, Sweden; 11 Transplant Institute, Sahlgrenska University Hospital, Gothenburg, Sweden; 12 School of Life Sciences, University of Essex, Colchester, UK; 13 Department of Biochemistry and Microbiology, University of Victoria, Victoria, British Columbia Canada; 14 SciLifeLab, University of Gothenburg, Gothenburg, Sweden

**Keywords:** Glycobiology, Microbiome, X-ray crystallography, Hydrolases

## Abstract

Excessive foraging of colonic mucin glycans by gut bacteria is associated with diseases such as inflammatory bowel disease. Although *Akkermansia muciniphila* is an important mucin degrader, the role of carbohydrate sulfatases that facilitate digestion of these heavily sulfated glycans remains unclear. Combining in vitro digestion assays, proteomics and structural biology, we show that *A. muciniphila* sulfatases, such as Amuc1755 and Amuc0953, have rare adaptations targeted towards known sulfated mucin structures. They show larger degrees of modularity, including a previously unknown mucin-binding domain. When grown on colonic mucin substrates, glycoproteins of reduced size were important for the growth of *A. muciniphila*. Further mutational analysis and localization studies revealed that desulfation of *N*-acetyl-d-glucosamine was periplasmic, while desulfation of d-galactose occurred extracellularly and in the periplasm. These data improve our understanding of contexts for the positive health correlations of *A. muciniphila* while metabolizing colonic mucin as its sole carbon source.

## Abstract (web summary)

Structural and biochemical follow-up of a serendipitous discovery reveals that *Akkermansia muciniphila* carbohydrate sulfatases are uniquely adapted to digest colonic mucins in collaboration with other members of gut microbiota.

## Main

The human colonic microbiota (henceforth microbiota) is a vast microbial community composed of hundreds of bacterial species that greatly impact the physiology of the host^1,2^. In the colon, the mucins of the host not only form a protective barrier that separates the microbiota from the host epithelial layer, but also serve as both a nutrient source and a colonization factor^3,4^. MUC2 is the major gel-forming colonic mucin, and up to 80% of its mass can comprise O-glycans^5^. Sulfation of mucin O-glycans is variable along the gastrointestinal tract, increasing from proximal to distal regions, with colonic MUC2 showing the highest sulfation^6^, up to 10% by mass^7^. This sulfation adds complexity and requires that colonic bacteria express carbohydrate sulfatases to fully metabolize colonic mucin^4,8^. All known carbohydrate sulfatases belong to the S1 family of sulfatases, which is divided into 110 subfamilies denoted S1_*X* (refs. ^9,10^).

A physiologically healthy mucosal barrier must be maintained for microbiota eubiosis^11,12^. Increased activity of microbiota carbohydrate-active enzymes can lead to excessive degradation of the mucin O-glycans^13,14^, disruption of the protective mucus barrier and diseases such as ulcerative colitis^15,16^, a type of inflammatory bowel disease. Indeed, some microbiota species, such as *Bacteroides thetaiotaomicron* and *Bacteroides vulgatus*, have been shown to drive ulcerative colitis in a sulfatase-dependent manner in a susceptible mouse model^16,17^, and it was shown that a single sulfatase was required for *B. thetaiotaomicron* to efficiently use colonic mucin oligosaccharides. Microbiota carbohydrate sulfatase activity is also increased in humans with active ulcerative colitis^14^, making these enzymes potential drug targets.

The mucin-specialist *Akkermansia muciniphila*, which can account for up to ~3% of the total microbiota^18,19^, encodes multiple carbohydrate sulfatases, but its prevalence in studies of disease association in humans was inversely correlated with disease and inflammation markers^20–22^ while, in animal models, its beneficial effects were dependent on the context^23^. On low-fibre diets, *A. muciniphila* increased pathogen susceptibility^24^ and food-driven allergy^25^, but on high-fibre diets, these effects were nullified^24^. Finally, increased *A. muciniphila* abundance in IL-10-deficient mice was sufficient to drive colitis-like disease^26^.

The *A. muciniphila* genome encodes 10 S1 carbohydrate sulfatases, from 6 subfamilies^10^, that are related to the mucin-targeting sulfatases described in *B. thetaiotaomicron*^4,27^. To date, most studies of *A. muciniphila* have focused on characterizing its glycoside hydrolases, using gastric mucins, which are low in sulfation. In gastric mucin, only the O6 position of *N*-acetyl-d-glucosamine (GlcNAc) is known to be sulfated, while sulfation of colonic mucin is known to occur on the O3, O4 and O6 positions of d-galactose (Gal), as well as the O6 position of GlcNAc^6,28^. Recent data, however, have shown that heavily sulfated colonic mucin glycoprotein, and not free colonic mucin oligosaccharides (cMOs), are needed to support growth^29,30^. Yet, no detailed study on the *A. muciniphila* carbohydrate sulfatases, and their role in metabolizing heavily sulfated colonic mucins, has been conducted. Such knowledge is critical to understanding the role of *A. muciniphila* carbohydrate sulfatases in the metabolism of heavily sulfated colonic mucin.

In this study, we used wild type and sulfatase transposon mutants to analyse the growth of *A. muciniphila* on colonic mucins, to reveal that glycoprotein fragments, rather than intact colonic mucin glycoproteins, are the preferred substrate of *A. muciniphila*. We provide detailed kinetic information for 8 of the 10 *A. muciniphila* S1 sulfatases, define their cellular location and structurally characterize 5 of these enzymes. We also identify the sulfatase-linked mucin-binding module that is restricted to *Akkermansia* species. These data inform the sulfobiology of this important microbiota member and reveal the adaptations that *A. muciniphila* has evolved to degrade colonic mucins.

## Results

### *A. muciniphila* requires glycoprotein forms of colonic glycans for growth

**Fig. 1:**
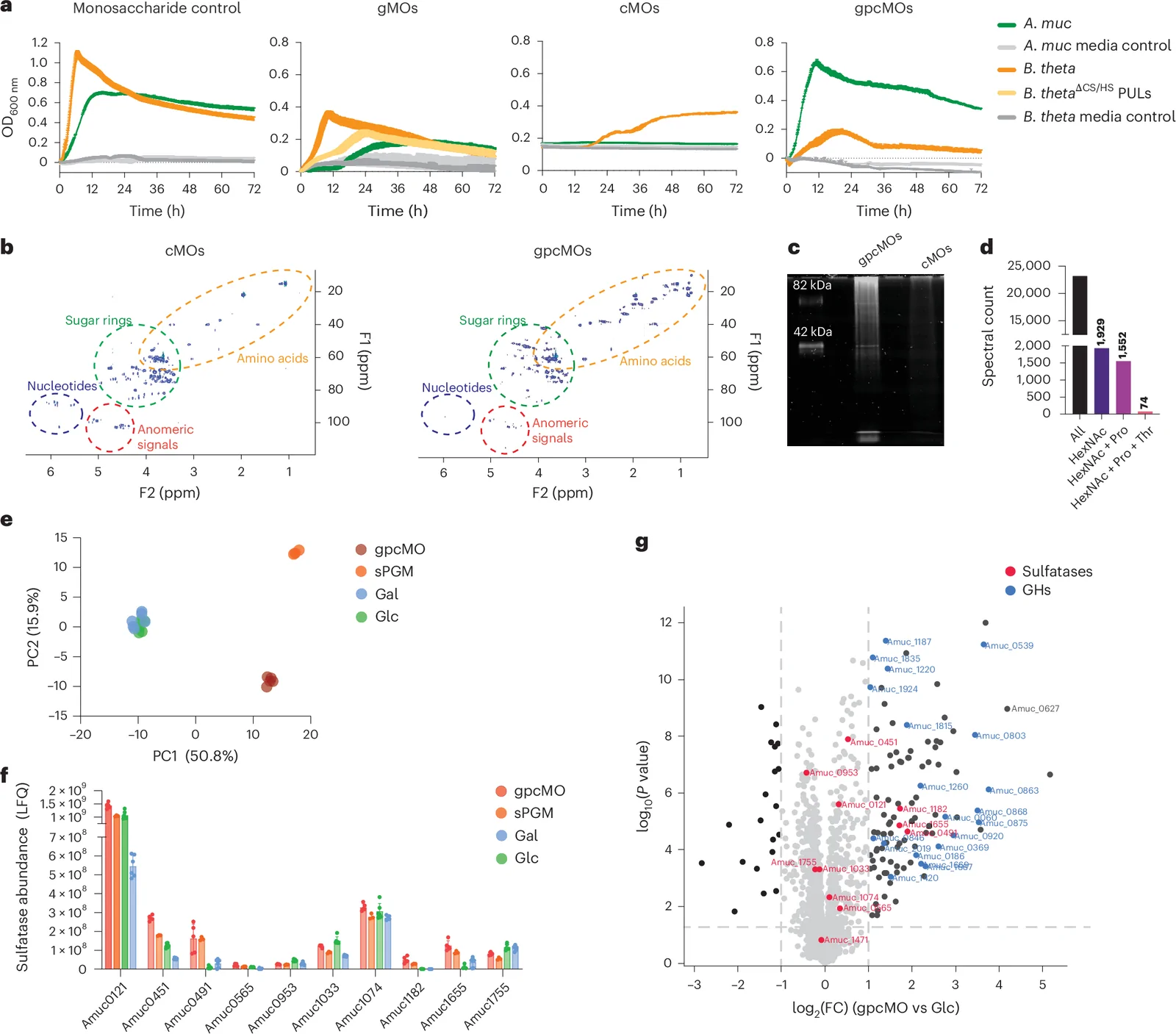
Growth profiles and protein expression of *A. muciniphila* on variable mucin structures. **a**, Growth profiles of *A. muciniphila* on low-sulfation and high-sulfation mucin O-glycans and glycopeptides and glycoproteins. All substrate concentrations were 5 mg ml^−1^ except GlcNAc, which was used at 10 mg ml^−1^ for *A. muciniphila* while glucose was used for *B. thetaiotaomicron* in the monosaccharide controls. **b**, NMR profiles of porcine cMOs and porcine colonic glycopeptides and glycoproteins. **c**, Glycoprotein gel staining of PCM oligosaccharides (cMOs) and porcine colonic glycopeptides and glycoproteins (gpcMOs). The gel is representative of three independent experiments. **d**, Spectral counts of glycopeptides and glycan-containing spectra identified in the porcine colonic glycoprotein fraction by mass spectrometry. HexNAc, hexosamine. **e**, Principal component analysis of global proteomic data. **f**, Total abundance of *A. muciniphila* sulfatases across proteomic samples from *A. muciniphila* grown on various carbon sources. LFQ, label-free quantification. Data were derived from five biological replicates and presented as mean ± s.d. **g**, Volcano plot showing protein abundance in *A. muciniphila* grown on gpcMOs compared with glucose. Differentially expressed proteins (fold change ≥ 2, *P* < 0.05) are indicated with dashed lines. *n* = 5 or 6. *P* values were determined using two-sided *t*-test; FDR = 0.05. Source data

**Extended Data Fig. 1:**
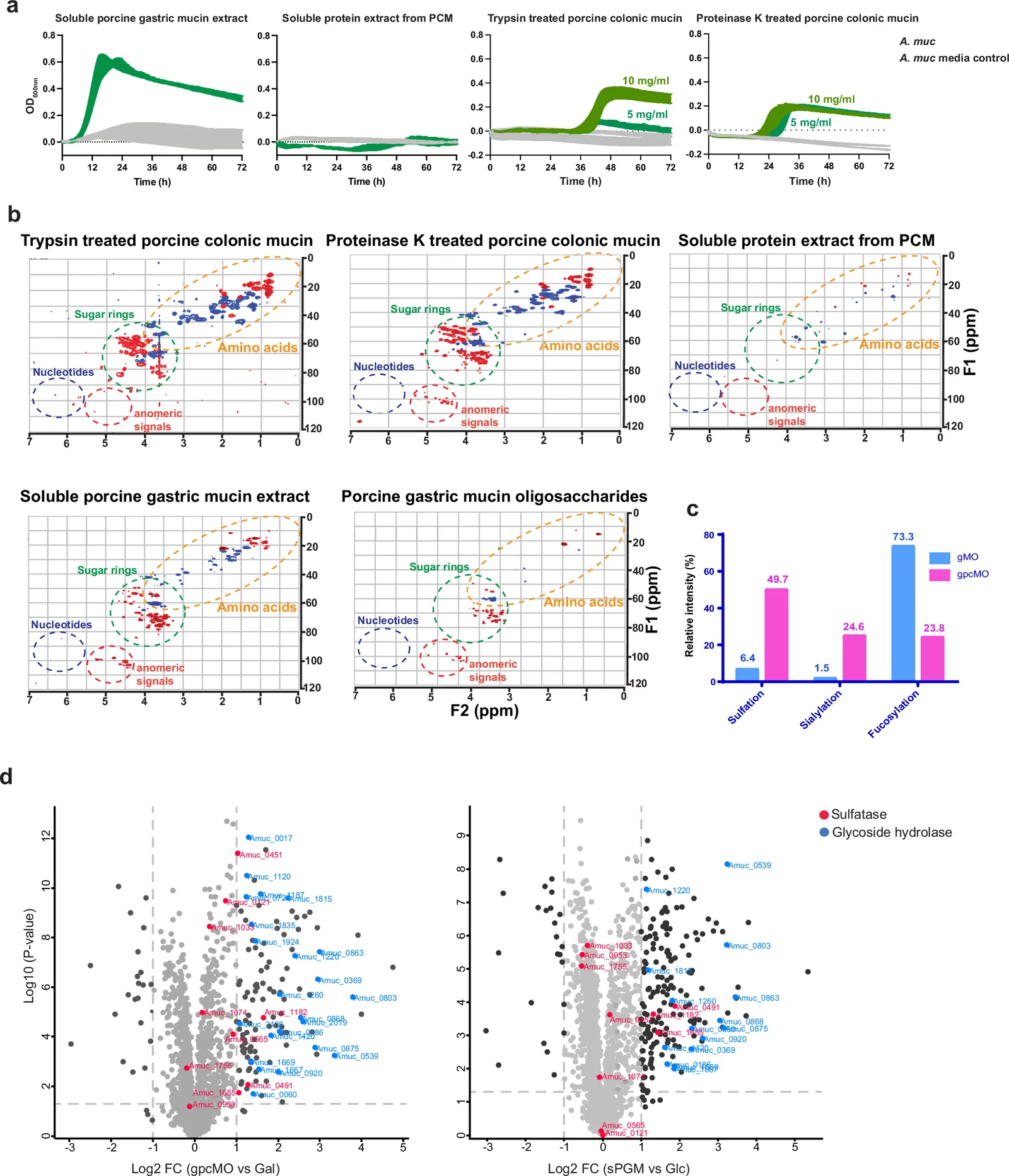
Growth profiles and protein expression of *A. muciniphila* on variable mucin structures. **a**. Growth profiles of *A. muciniphila* on low sulfation and high sulfation soluble mucins. All substrate concentrations are 5 mg/ml unless stated. **b**. HSQC 2-D NMR profiles of various mucin or mucin derived samples examining their relative protein to glycan content. **c**. Bar chart showing the percentages of sulfated and unsulfated glycans detected by mass spectrometry in gMO and gpcMO samples. The data were taken from supplementary dataset 2 and the numbers in the bars indicate the number of individual glycans identified **d**. Volcano plots showing protein abundance from A. muciniphila grown on gpcMOs compared to galactose and *A. muciniphila* grown on sPGM compared to glucose. Differentially expressed proteins (fold-change ≥ 2, P value < 0.05) are indicated with dashed lines. Sulfatases marked with red, glycoside hydrolases marked with blue. n = 3-6. P-value determined from two-sided t-test, FDR = 0.05. Source data

We initially established growth profiles of *A. muciniphila* on soluble porcine gastric mucin (sPGM) and gastric mucin oligosaccharides (gMOs) released from sPGM glycoprotein. *A. muciniphila* showed robust growth on sPGM, while growth on gMOs was much weaker (Fig. 1a and Extended Data Fig. 1a). By contrast, *B. thetaiotaomicron* and a mutant unable to use contaminating glycosaminoglycans (*B*. *thetaiotaomicron*^ΔCS/HS^) grew robustly on gMOs (Fig. 1a). *A. muciniphila* was unable to grow on glycosaminoglycans or glycosaminoglycan breakdown products (Supplementary Fig. 1). We next examined growth on a soluble protein fraction extracted from porcine colonic mucin (PCM) containing only protein (~5.0% MUC2 fragments and ~95% cellular protein from cell lysis; Supplementary Dataset 1) and cMOs. *A. muciniphila* was incapable of growth on these substrates, while *B. thetaiotaomicron* growth was supported by cMOs^4^ (Fig. 1a and Extended Data Fig. 1a). After the preparation of a new batch of cMOs, we observed strong growth for *A. muciniphila*, while *B. thetaiotaomicron* was also able to use this substrate, but to a lower degree (Fig. 1a). We hypothesized that differences in available glycans between the two substrate batches were responsible for the differences in growth profile. Comparison of the substrates by nuclear magnetic resonance spectroscopy (NMR) (Fig. 1b and Extended Data Fig. 1b), glycoprotein gel staining (Fig. 1c) and mass spectrometry (Fig. 1d, Extended Data Fig. 1c and Supplementary Dataset 2) revealed that the second batch of cMOs was enriched in glycopeptides and glycoproteins and their NMR profile was more similar to that of sPGM. For this reason, cMO batch 2 is termed glycopeptide and glycoprotein colonic mucin oligosaccharides (gpcMOs). Following this, we treated PCM with the proteases trypsin and proteinase K to generate soluble mucin glycoproteins. These hydrolysed substrates showed similar NMR profiles to gpcMOs and sPGM (Extended Data Fig. 1b), but supported growth of *A. muciniphila* to lower levels (Extended Data Fig. 1a); growth on trypsin-treated material required a double substrate concentration for robust growth. These data reveal that *A. muciniphila* targets glycopeptide and glycoprotein forms of mucin and that these are needed for robust growth on gastric mucins, but are essential for growth on colonic mucins. By contrast, *B. thetaiotaomicron* could grow on gMOs, cMOs and gpcMOs (Fig. 1a). This indicates that *A. muciniphila* targets specific glycopeptides or glycoproteins of a particular size and/or motif that were serendipitously captured in our gpcMO preparation, and these cannot be fully recapitulated by trypsin or proteinase K treatment.

To determine whether the increased sulfation present in colonic mucin drove increased expression of *A. muciniphila* carbohydrate sulfatases, we performed proteomic analysis of *A. muciniphila* grown on d-glucose (Glc), Gal, sPGM and gpcMOs. Although the samples cluster differently from each other (Fig. 1e), all 10 *A. muciniphila* sulfatases were detected under all conditions (Fig. 1f). When comparing gpcMOs with Glc samples, only three sulfatases (Amuc0491, Amuc1655 and Amuc1182) were more upregulated (Fig. 1g). Overall, the different gastric and colonic mucin substrates did not lead to large differences in sulfatase expression (Extended Data Fig. 1d and Supplementary Dataset 3).

### *A. muciniphila* sulfatases desulfate both model and colonic oligosaccharide substrates

**Fig. 2:**
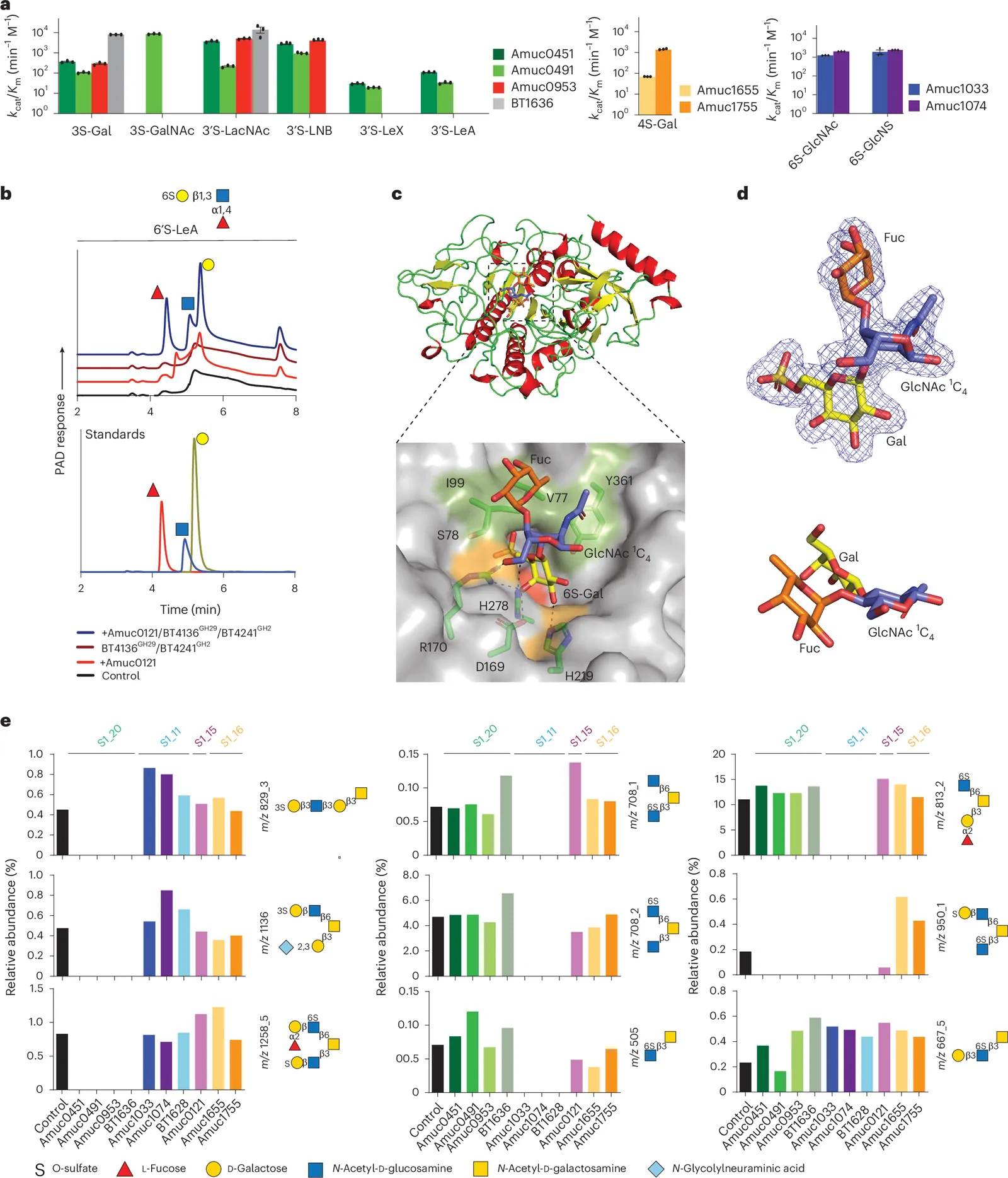
Substrate preference and activity of S1 *A. muciniphila* sulfatases. **a**, Kinetic activity for sulfatases for which an activity could be identified. Assays were carried out in an appropriate buffer for the pH optimum for each respective enzyme with 1 μM of a fluorescently labelled substrate, 150 mM NaCl and 5 mM CaCl_2_. LeX, Lewis X; LeA, Lewis A. Data were derived from three technical replicates and presented as mean ± s.e.m. **b**, HPAEC analysis showing that Amuc0121^6S-Gal^ is essential to the breakdown of O6 Gal sulfated Lewis A structures. BT4136 is a GH29 fucosidase capable of acting on α1,3/4 linkage, while BT4241 is a GH2 enzyme targeting β1,3 glycosidic linkages; these enzymes were at a final concentration of 1 μM. The final concentration of Amuc0121^6S-Gal^ was 67 μM. Assays were carried out in 10 mM MOPS, pH 7.0, with 150 mM NaCl at 37 °C overnight. **c**, Cartoon representation of the Amuc0121^6S-Gal^ crystal structure with 6′S-Lewis A and a zoomed-in surface representation with key residues highlighted beneath. In orange is the galactose recognition triad while green highlights the hydrophobic patch that accommodates Fuc (l-fucose). **d**, The weighted 2mFobs-DFc electron density map, contoured at 1*σ*, for the 6′S-Lewis A substrate from **e**. GlcNAc adopts a strained ^1^C_4_ conformation. Below this is a relaxed Lewis A substrate with GlcNAc in a ^4^C_1_ conformation from Protein Data Bank (PDB): 6OR4. **e**, Relative abundance of processed glycan species generated by the activity of *A. muciniphila* sulfatases on cMOs, as assessed by mass spectrometry. Source data

**Extended Data Fig. 2:**
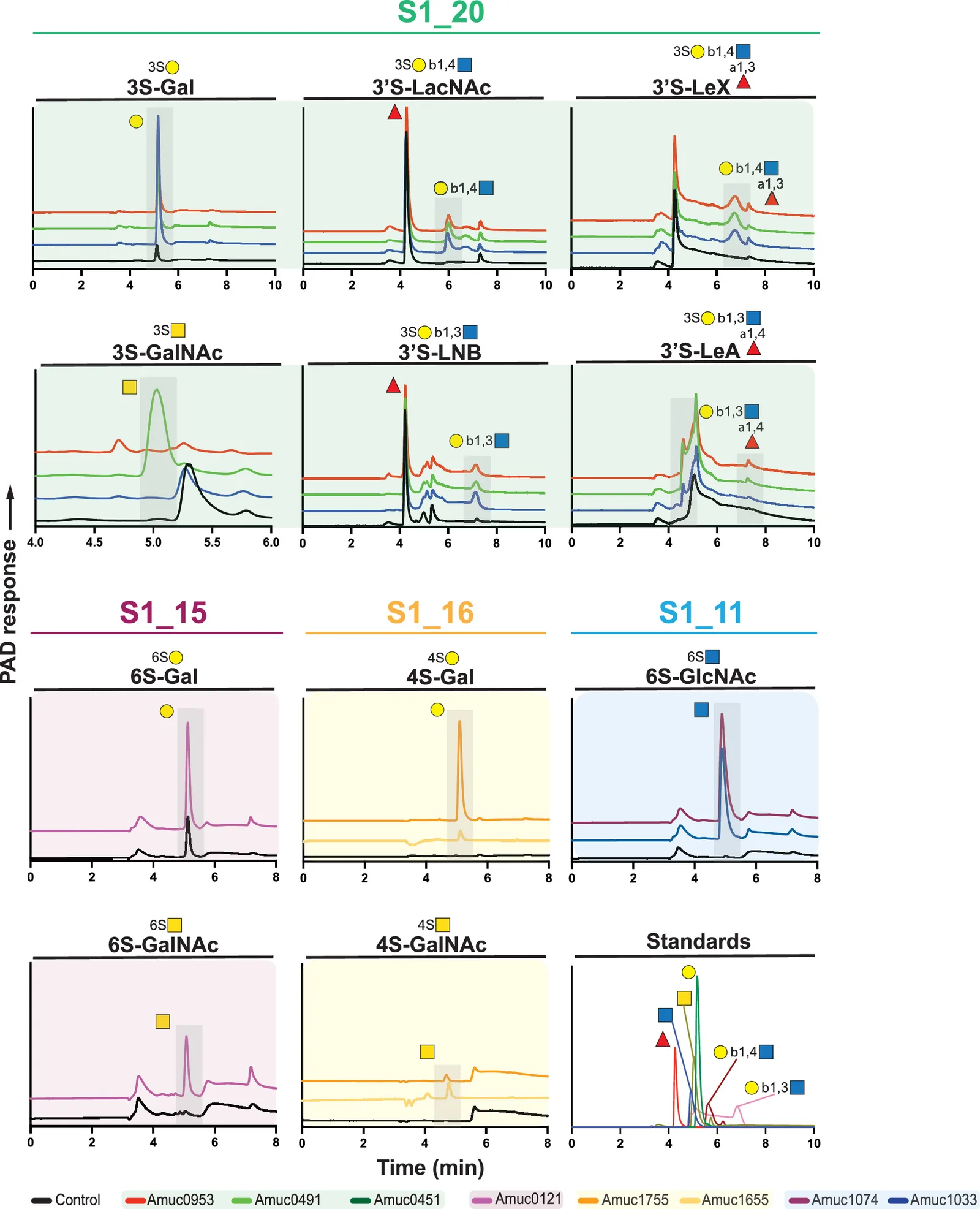
Activity of *A. muciniphila* sulfatases against model sulfated oligosaccharides. High performance anion exchange chromatography (HPAEC) traces showing the activity of S1 *A. muciniphila* sulfatases on model substrates representing sulfated linkages found in colonic mucin. HPAEC traces are from qualitative overnight assays using 5 mM substrate and 5 μM enzyme carried out a 37 °C in 5 mM MOPS pH 7.0 with 150 mM NaCl and 5 mM CaCl_2_. Source data

To understand the biochemistry of how *A. muciniphila* desulfates mucin O-glycans, we expressed all 10 S1 sulfatases recombinantly and detected activities for 8 of the 10 enzymes (Fig. 2a and Extended Data Fig. 2). Only Amuc0565 and Amuc1182, from subfamilies S1_4 and S1_19, showed no activity against any substrates tested. This is in line with a recently published qualitative study^31^. To define the true substrate specificity of each sulfatase, we determined the catalytic efficiency using fluorescently labelled substrates (Supplementary Fig. 2 and Supplementary Table 1) as well as on model and mucin-derived glycans (Fig. 2a,b and Extended Data Fig. 2).

*A. muciniphila* contains three S1_20 sulfatases, Amuc0451^3S-LacNAc/LNB^, Amuc0491^3S-Gal/GalNAc^ and Amuc0953^3S-LacNAc/LNB^, which desulfate O3 sulfated d-galactose (3S-Gal)-containing substrates (Fig. 2a and Extended Data Fig. 2) although only Amuc0491^3S-Gal/GalNAc^ was capable of desulfating O3 sulfated*N*-acetyl-d-galactosamine (3S-GalNAc) (Fig. 2a and Extended Data Fig. 2). Amuc0451^3S-LacNAc/LNB^ and Amuc0953^3S-LacNAc//LNB^ desulfated 3S-Gal, but were tenfold more active on O3 sulfated lactosamine (3′S-LacNAc) and O3 sulfated lacto-*N*-biose (3′S-LNB), and weakly active on 3′S-Lewis X and 3′S-Lewis A (Fig. 2a).

**Extended Data Fig. 3:**
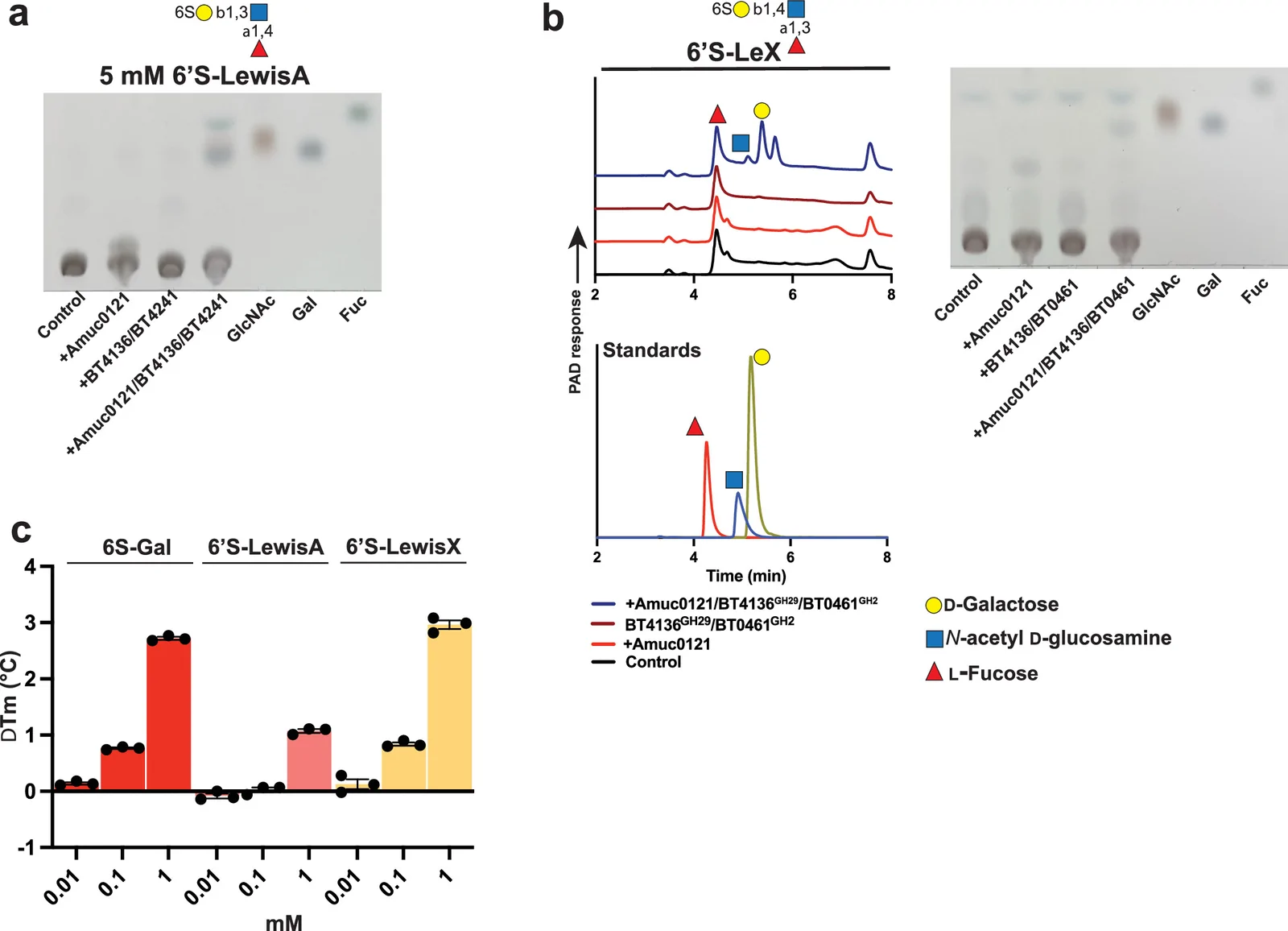
Activity and affinity of Amuc0121^6S-Gal^ for Lewis antigens. **a**. Thin-layer chromatography (TLC) demonstrating Amuc0121^6S-Gal^ is essential to breakdown *O*6 Gal sulfated LewisA structures. BT4136 is a GH29 fucosidase capable of acting on α1,3/4 linkage whilst BT4241 is a GH2 enzyme preferentially targeting β1,3, glycosidic linkages; enzymes were at a final concentration of 1 μM. The final concentration of Amuc0121^6S-Gal^ was 67 μM. Assays were carried out in 10 mM HEPES, pH 7.0. with 150 mM NaCl at 37 °C overnight. **b**. left and right panels are high performance anion exchange chromatography (HPAEC) and TLC data, respectively the breakdown of 6’S-LewisX by 67 μM Amuc0121^6S-Gal^ alone, or in combination with the fucosidase BT4136^GH29^ and the galactosidase BT0461^GH2^ a GH2 enzyme preferentially targeting β1,4, glycosidic linkages; enzymes were at a final concentration of 1 μM. Assays were carried out a 37 °C in 5 mM MOPS pH 7.0 with 150 mM NaCl. Samples were diluted 1:100 in water for HPAEC analysis. **c**. Thermostability shift assays of 5 μM Amuc0121^6S-Gal^ against 6S-Gal, 6’S-LewisA, and 6’S-LewisX. Assays were carried out in 100 mM HEPES pH 7.0, or BTP pH 7.0, with 150 mM NaCl. Data are from 3 technical replicates with the standard error of the mean shown. Source data

**Extended Data Fig. 4:**
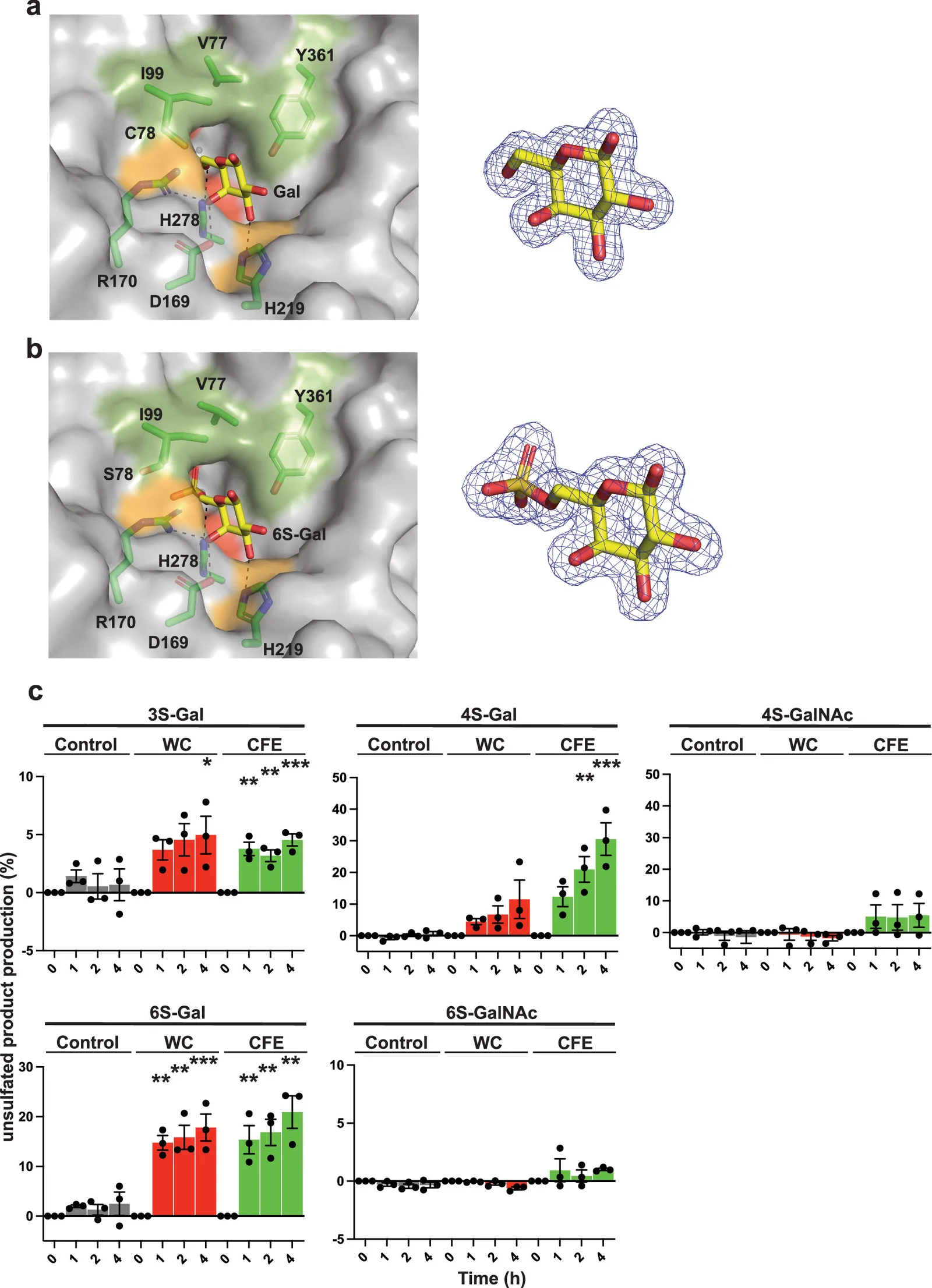
Crystal structures and activity of Amuc0121^6S-Gal^ and cellular localisation assays of *N*-acetyl-D-glucosamine grown *A. muciniphila* cells. **a**, **b**. Surface representation of the Amuc0121^6S-Gal^ crystal structure in complex with D-galactose and *O*6 sulfated D-galactose, respectively. Key residues are shown as sticks beneath. In orange is the galactose recognition triad whilst green highlights the hydrophobic patch that accommodates Fuc (L-fucose); 6S-Gal is O6 sulfated D-galactose while GlcNAc is *N*-acetyl-D-glucosamine. In each case, the weighted 2mFobs-DFc electron density map, contoured at 1 σ, is shown on the right. **c**. HPAEC-based cell assays with wildtype and transposon mutant sulfatases. Cells were grown with *N*-acetyl-D-glucosamine (GlcNAc) as their carbon source, to mid-exponential phase before being left in aerobic conditions for ~30 mins to arrest growth. These were either left as whole cells (WC) or prepared as cell free extracts (CFE), and 1 μM of a fluorescent substrates added to the media and sampled periodically. Experiments are biological triplicates presented as mean values +/- SEM. Statistical significance of time points versus control was calculated using a multiple comparison one way ANOVA; a single asterisk indicates a P value < 0.05, two <0.005, three <0.0005, and four <0.0001. Nonzero subtracted data is in supplementary Fig. 4c. Source data

A single S1 sulfatase, Amuc0121^6S-Gal/GalNAc^, from S1_15 was found to desulfate both O6 sulfated d-galactose (6S-Gal) and O6 sulfated GalNAc (6S-GalNAc) (Extended Data Fig. 2), as well as the trisaccharides 6′S-Lewis A and 6′S-Lewis X (Fig. 2b and Extended Data Fig. 3a,b), and is the only enzyme known to break down these structures. Thermal shifts for 6S-Gal and 6′S-Lewis X were similar while those for 6′S-Lewis A were lower, indicative of a weaker interaction (Extended Data Fig. 3c). Crystal structures of Amuc0121^6S-Gal/GalNAc^ in complex with Gal, 6S-Gal and 6′S-Lewis A reveal that Gal is bound identically in all three with O1 pointing into solvent (Fig. 2c and Extended Data Fig. 4a,b). Unexpectedly, the binding of 6′S-Lewis A requires the GlcNAc to adopt an atypical, strained, ^1^C_4_ chair conformation instead of the lower-energy ^4^C_1_ (Fig. 2d). This allows the Fuc to sit above a hydrophobic patch created by the conserved residues Val77, Ile99 and Tyr361 (ref. ^27^), while the O6 of GlcNAc interacts with the conserved residue Arg170 that is part of the Gal recognition triad within roughly half of S1_15 members^27^; a similar binding mode is likely to be adopted by 6′S-Lewis X (Supplementary Fig. 3). No kinetic measurement could be accurately made for the Amuc0121^6S-Gal/GalNAc^ probably owing to lower installation of the catalytic formylglycine nucleophile by *Escherichia coli* during recombinant expression, reported previously for the S1_15 subfamily^27^.

The two S1_16 sulfatases, Amuc1655^4S-Gal^ and Amuc1755^4S-Gal^, were shown to have specificity for O4 sulfated d-galactose (4S-Gal) with Amuc1755^4S-Gal^ appearing to be ~10-fold more efficient than Amuc1655^4S-Gal^ (Fig. 2a). However, both enzymes show very weak desulfation of O4 sulfated (4S-GalNAc) (Extended Data Fig. 2). The final two characterized enzymes were Amuc1033^6S-GlcNAc^ and Amuc1074^6S-GlcNAc^. The enzymes belong to S1_11 and were able to desulfate either 6S-GlcNAc or O6 sulfated *N*-sulfo-d-glucosamine BODIPY-labelled substrates with equal efficiency (Fig. 2a).

Having determined activities on model substrates, we next incubated these enzymes with cMOs and observed the effect on the relative abundance of each glycan by mass spectrometry (Fig. 2e and Supplementary Dataset 4). Only the 3S-Gal/GalNAc and 6S-GlcNAc targeting sulfatases were active on these cMOs. No difference in activity was observed between the three S1_20 or the two S1_11 enzymes, and all the five sulfatases were only active on terminal sulfated linkages (Fig. 2e). These results indicated that *A. muciniphila* encodes sulfatases to remove the main sulfated linkages found in complex cMOs and uses the same S1 subfamilies as reported for *B. thetaiotaomicron*^4^.

### *A. muciniphila* restricts GlcNAc desulfation to the periplasm

**Fig. 3:**
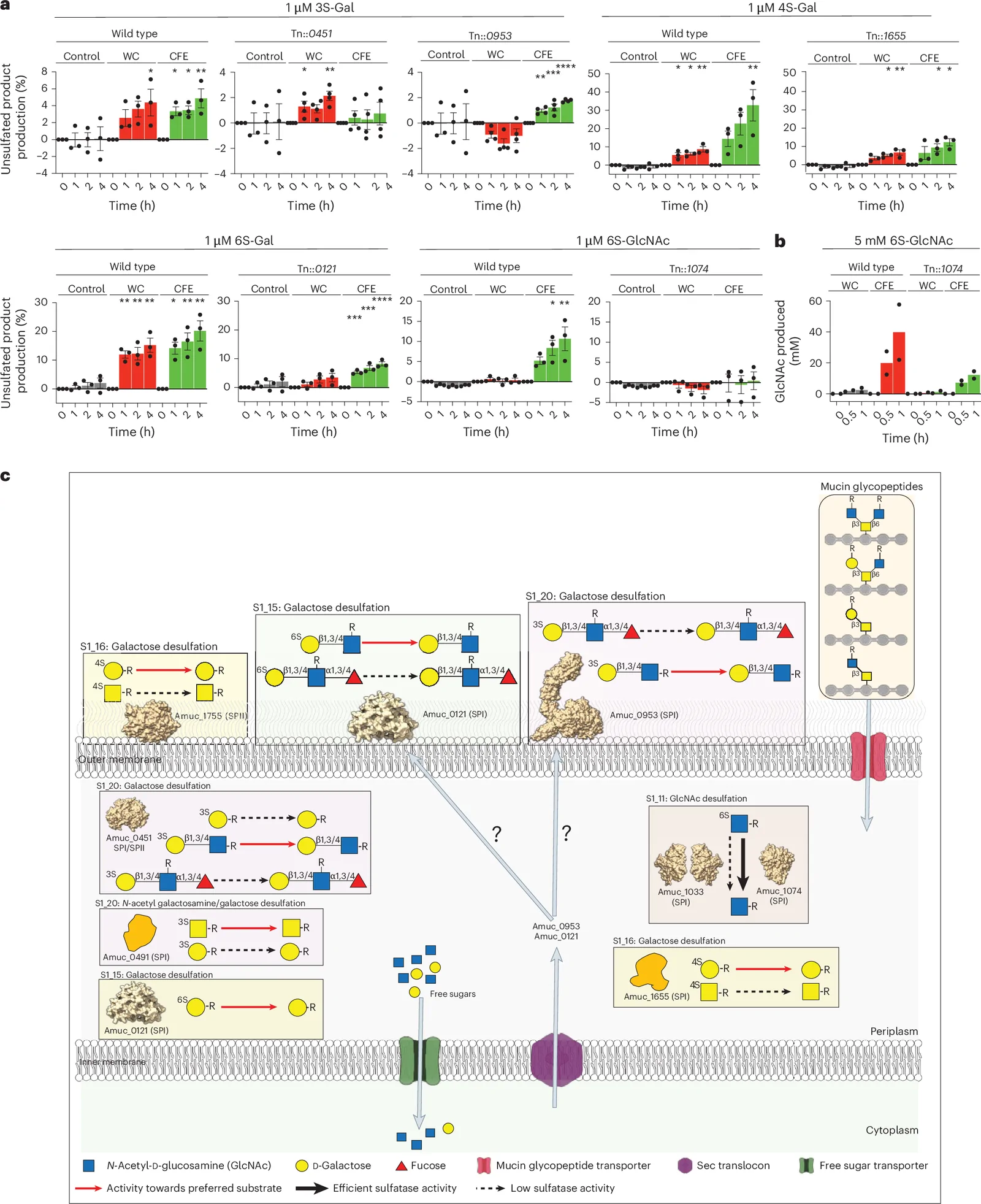
Cell-based assays and sulfatase cellular location. **a**, HPAEC-based cell assays with wild-type and transposon mutant sulfatases. Cells were grown, with sPGM as their carbon source, to mid-exponential phase before being left in aerobic conditions for ~30 min to arrest growth. These were either left as whole cells (WC) or prepared as CFEs, and 1 μM of a fluorescent substrate was added to the media and sampled periodically. Experiments were biological triplicates except for tn::*0451* and tn::*0953* which are biological quadruplicates presented as mean ± s.e.m. Control samples were cells that had been heat deactivated at 95 °C for 10 min. Statistical significance of time points versus control was calculated using multiple-comparison one-way ANOVA; *P < 0.05; **P < 0.005; ***P < 0.0005; ****P < 0.0001. Non-zero subtracted data are available in Supplementary Fig. 4a. **b**, HPAEC-based cell assays with wild-type and transposon mutant sulfatases. Cells were grown to mid-exponential phase before being left in aerobic conditions for ~30 min to arrest growth. These were washed with PBS and either left as WCs or prepared as CFEs before adding 5 mM 6S-GlcNAc. These experiments were carried out as biological duplicates. Non-zero subtracted data are available in Supplementary Fig. 4b. **c**, A schematic model of sulfatase cellular location and the substrates targeted. Source data

To reveal the cellular localization of *A. muciniphila* sulfatases, we performed assays using whole cells and cell-free extracts (CFEs) using wild-type bacteria and sulfatase transposon mutants grown on GlcNAc and sPGM. Initially, we attempted to centrifuge the cells and wash them with phosphate-buffered saline (PBS), but this resulted in substantial cell lysis (Supplementary Table 2). To avoid lysis, we used a high-performance anion exchange chromatography (HPAEC) method to detect fluorescent substrates that were sampled directly from the culture media under aerobic conditions (Fig. 3a–c, Extended Data Fig. 4c and Supplementary Fig. 4).

Assays with wild-type *A. muciniphila* with intact whole cells and CFE indicated that O3 desulfation of galactose occurs both extra- and intracellularly (Fig. 3a,c and Extended Data Fig. 4c). Assays with the *amuc0953::*Tn mutant reveal loss of extracellular activity but retention of activity with CFEs. The reciprocal was observed for the *amuc0451*::Tn mutant. These data indicated that Amuc0953^3S-LacNAc/LNB^ is responsible for the extracellular activity and Amuc0451^3S-LacNAc/LNB^ for the intracellular activity. Amuc0953^3S-LacNAc/LNB^ possesses an signal peptidase I (SPI) signal peptide that drives localization to the periplasm; thus, an unknown mechanism must translocate the protein to the extracellular space.

Degradation of 4S-Gal was observed from whole cells, which increased with CFE, indicating the presence of 4S-Gal sulfatase activity both extra- and intracellularly (Fig. 3a and Extended Data Fig. 4c). The *amuc1655*::Tn mutant showed decreased activity from the CFE indicating that Amuc1655^4S-Gal^ is periplasmic. The remaining activity observed for whole cells suggests that Amuc1755^4S-Gal^ is located at the cell surface and is consistent with it possessing an SPII signal peptide enabling it to be membrane anchored (Fig. 3a).

Desulfation of 6S-Gal was observed for whole cells and CFEs, with this activity being largely lost with the transposon mutant *amuc0121*::Tn (Fig. 3a) indicating that Amuc0121^6S-Gal/GalNAc^ is localized to the cell surface and the periplasm. The open active site of Amuc0121^6S-Gal/GalNAc^ observed in the crystal structure (Fig. 3a and Extended Data Fig. 4a–c) would facilitate accommodating more complex glycans found at the cell surface.

Finally, 6S-GlcNAc activity is exclusively observed with CFEs, but this activity was lost with the *amuc1074*::Tn mutant indicating that Amuc1074^6S-GlcNac^ is located in the periplasm (Fig. 3a and Extended Data Fig. 4c). No 6S-GlcNAc activity was observed for GlcNAc-grown cells indicating that high GlcNAc concentrations inhibit Amuc1074^6S-GlcNac^ (Extended Data Fig. 4c). Assays repeated using 5 mM 6S-GlcNAc, instead of 1 µM, showed that CFEs from the *amuc1074*::Tn mutant are able to release GlcNAc at these higher concentrations (Fig. 3b). This suggests that the second 6S-GlcNAc sulfatase, Amuc1033^6S-GlcNAc^, is also located in the periplasm. Overall, these results revealed that O3, O4 and O6 desulfation of Gal occurs both extra- and intracellularly while the desulfation of 6S-GlcNAc occurs exclusively in the periplasm.

### Amuc1074 is the main 6S-GlcNAc sulfatase in the periplasm

**Fig. 4:**
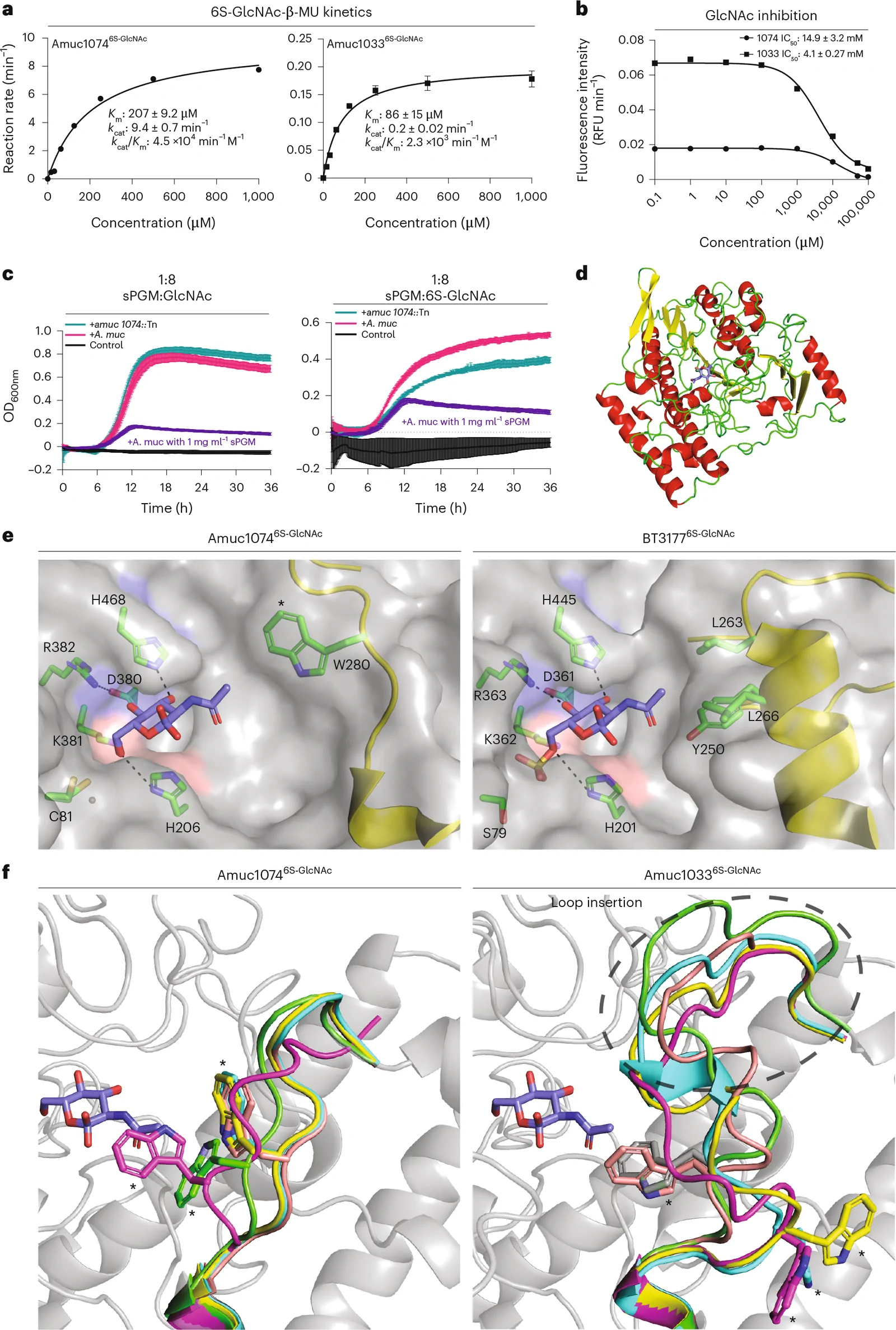
*A. muciniphila* has a low- and high-activity periplasmic S1_11 sulfatase. **a**, Michaelis–Menten kinetics of Amuc1074^6S-GlcNAc^ and Amuc1033^6S-GlcNAc^ versus 6S-GlcNAc-MU. Data points are derived from technical triplicates presented as mean ± s.e.m. **b**, IC_50_ curves measuring the ability of GlcNAc to inhibit Amuc1074^6S-GlcNAc^ and Amuc1033^6S-GlcNAc^. Data points are derived from technical triplicates presented as mean ± s.e.m. RFU, relative fluorescence units. **c**, Growth assays of wild-type *A. muciniphila* and *amuc1074*::Tn on GlcNAc, 6S-GlcNAc and 1:8 ratios of sPGM:GlcNAc and sPGM:6S-GlcNAc. The ratios are 1 mg ml^−1^ of sPGM and 8 mg ml^−1^ of the respective monosaccharide. The growth supported by 1 mg ml^−1^ of sPGM is shown for reference **d,** Crystal structure of showing the overall tertiary fold of Amuc1074^6S-GlcNAc^; α-helices, β-strands and loops are coloured red, yellow and green, respectively. **e**, Crystal structure of the glycan-binding site of Amuc1074^6S-GlcNAc^, solved to 1.38 Å resolution, with GlcNAc compared with the *B. thetaiotaomicron* enzyme BT3177^6S-GlcNAc^ (PDB: 7P24). A hydrophobic interaction with the N-acetyl group is key for high GlcNAc recognition. **f**, AF2 models of Amuc1074^6S-GlcNAc^ and Amuc1033^6S-GlcNAc^ showing the predicted flexibility of the respective N-acetyl recognition loops. An asterisk marks the position of the key Trp residue that interacts with the N-acetyl group of GlcNAc, which has also been added to the Amuc1074^6S-GlcNAc^ crystal structure in **d**. The differing loop colours indicate the five different predictions of the loop confirmation from the AF2 prediction. Source data

The two S1_11 enzymes, dimeric Amuc1033^6S-GlcNAc^ and monomeric Amuc1074^6S-GlcNAc^ (Supplementary Fig. 5a), desulfate 6S-GlcNAc-BODIPY with similar catalytic efficiencies (Fig. 2a). This fluorescent label has a six-atom linker and undergoes mutarotation between α and β anomers. However, when we used 6S-GlcNAc β linked to methylumbelliferone (6S-GlcNAc-MU), which more closely mimics a natural glycosidic linkage, we observed ~25-fold lower *k*_cat_/*K*_m_ (ratio of the turnover number (*k*_ca__t_) to the Michaelis constant (*K*_m_)) for Amuc1033^6S-GlcNAc^ (Fig. 4a). Both enzymes had similar affinities for both substrate and product when 6S-GlcNAc-MU was the substrate (Fig. 4a,b). The much lower catalytic efficiency observed for Amuc1033^6S-GlcNAc/GlcNS^ is entirely driven by its ~50-fold lower *k*_cat_ (Fig. 4a).

The lower efficiency and expression (Figs. 1f and 4a) of Amuc1033^6S-GlcNAc^ were born out in growth assays in which 6S-GlcNAc is the main carbon source. The *amuc1074*::Tn mutant, in which Amuc1033^6S-GlcNAc^ is the main 6S-GlcNAc periplasmic sulfatase, is unable to fully metabolize 6S-GlcNAc with a lower growth rate and maximum optical density compared with the wild type (Fig. 4c). No phenotype is observed when GlcNAc is the main carbon source.

**Extended Data Fig. 5:**
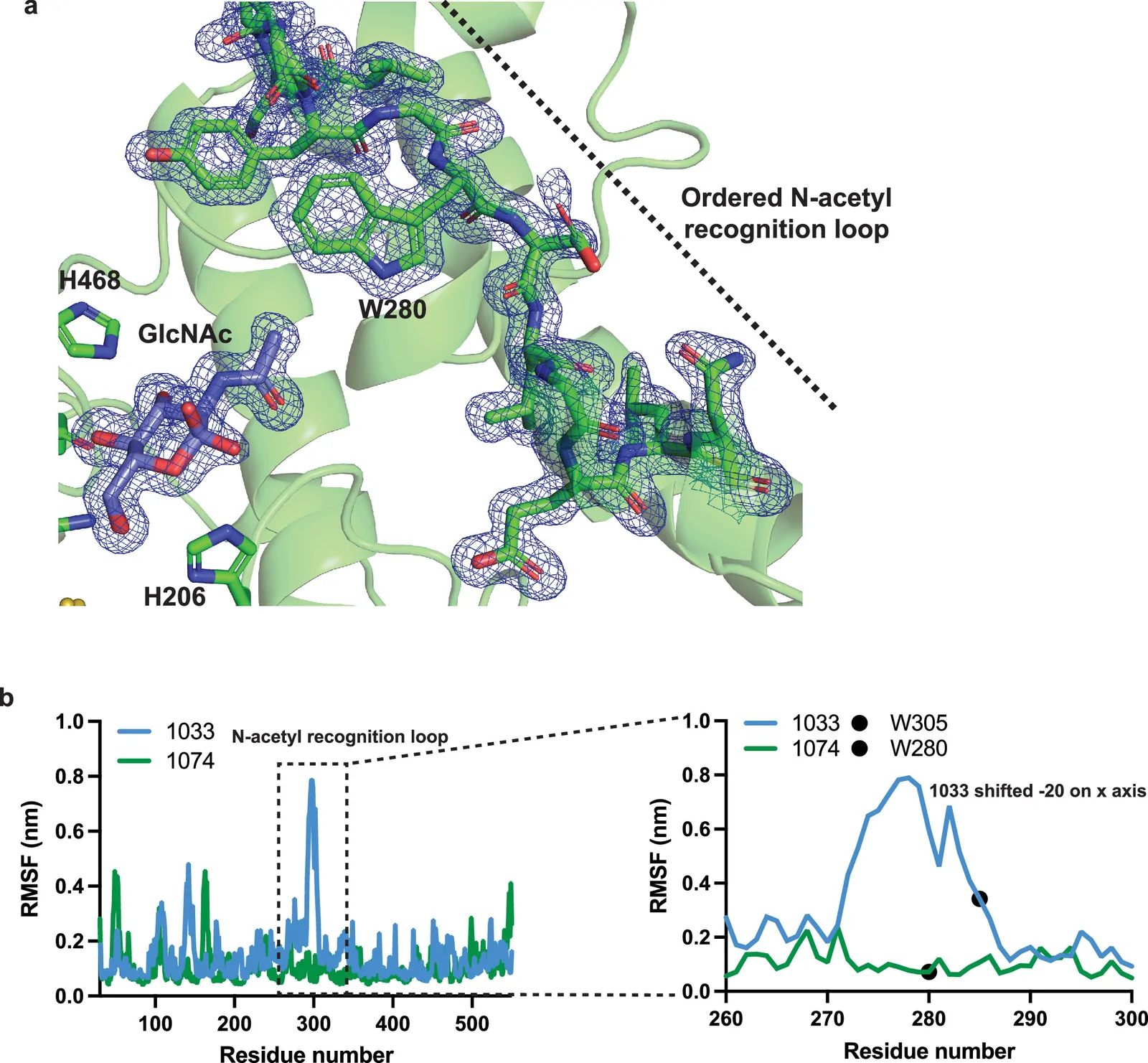
Structure and dynamics of the S1_11 enzymes *N*-acetyl recognition loop structure. **a**. Cartoon and stick representation of the *N*-acetyl recognition loop of Amuc1074^6S-GlcNAc^ in complex with GlcNAc; the weighted 2mFobs-DFc map has been contoured at 1 σ. **b**. Molecular dynamic simulations of unliganded Amuc1033^6S-GlcNAc^ and Amuc1074^6S-GlcNAc^ showing the R.M.S.F. values per residue with a zoomed-in graph of the N-acetyl recognition loop region. Source data

**Extended Data Fig. 6:**
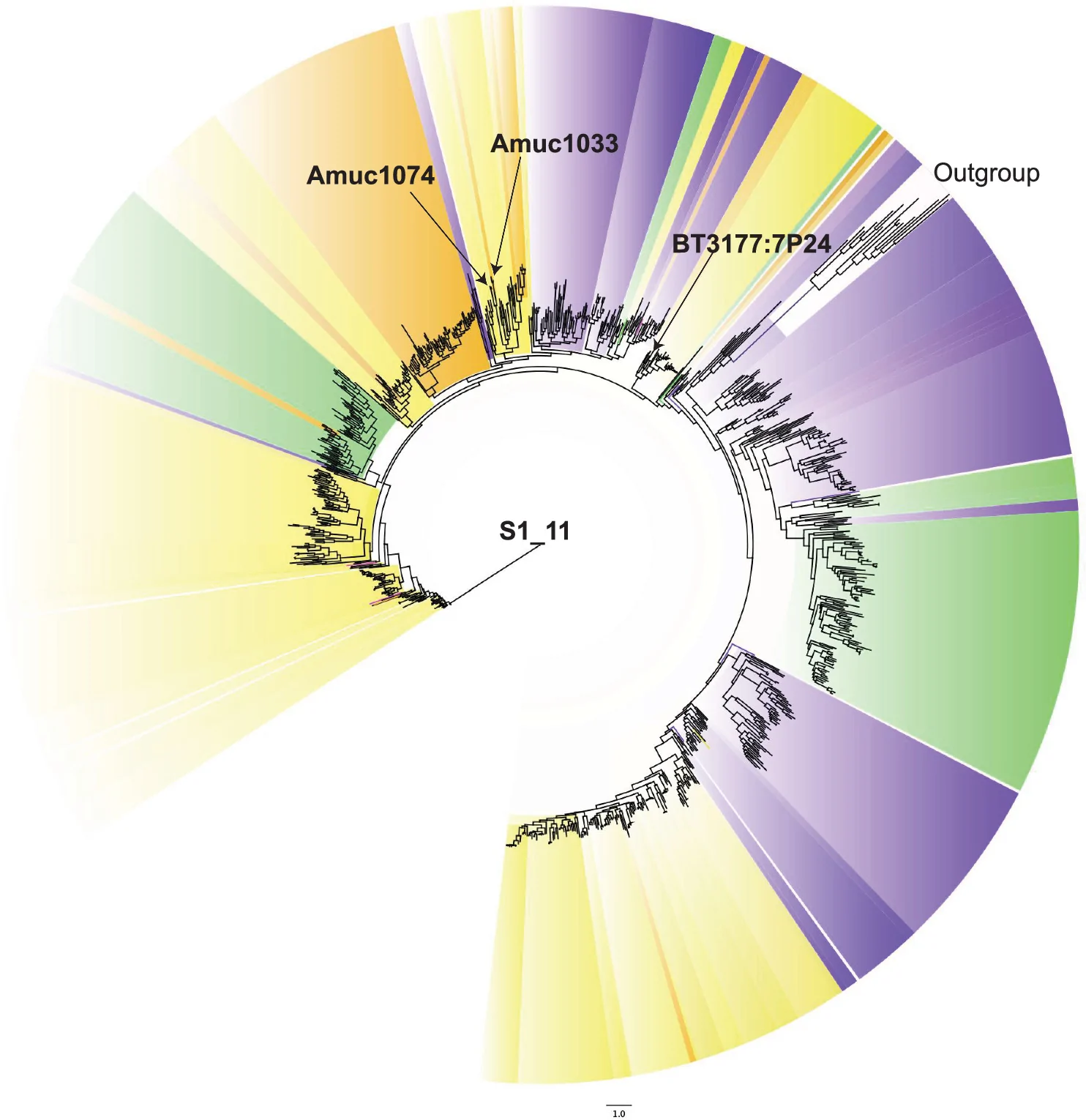
Phylogenetic tree of the S1_11 subfamily. Phylogenetic tree of 970 selected, most representative, sequences belonging to S1_11. The colours distinguish the sequences according to different lengths of the *N*-acetyl recognition loop, which in addition, is highly variable in amino acid composition. Absence of loops and those with fewer than 5 amino acids are coloured in purple, those having 5 to 11 amino acids are coloured in green. Sequences having loops with lengths ranging from 12 to 19 and hydrophobic patches similar to Amuc1074 are coloured in yellow and those having loops longer than 19 amino acids (in general 20 to 25) are coloured in orange. Since the hydrophobic character (Trp in Amuc1074 and Amuc1033) is key for its interaction with the *N*-acetyl group of GlcNAc, some isolated sequences having loops without hydrophobic character are highlighted by pink lines.

The structure of Amuc1074^6S-GlcNAc^ in complex with GlcNAc reveals that it has similar critical recognition features to *B. thetaiotaomicron* enzyme BT3177^6S-GlcNAc/GlcNS^ (Fig. 4d,e and Extended Data Fig. 5a). The N-acetyl recognition loop is highly variable in amino acid composition and is absent in half of the S1_11 subfamily (Extended Data Fig. 6), but a hydrophobic character is key for its interaction and Amuc1074^6S-GlcNAc^ and Amuc1033^6S-GlcNAc^ both deploy a Trp residue to interact with the N-acetyl group. The difference in efficiency between the two enzymes is due to Amuc1033^6S-GlcNAc^ containing a loop insertion before the N-acetyl recognition loop creating greater flexibility, shown by Alphafold 2 (AF2) modelling and molecular dynamic simulations (Fig. 4f and Extended Data Fig. 5b). This leads to a substantially reduced *k*_cat_, but not *K*_m_, indicating reduced transition state stabilization. The presence of Trp in the N-acetyl recognition with an extended loop is rare in S1_11 (Extended Data Fig. 6). *A. muciniphila* has a strong preference for GlcNAc as a carbon source^32^ (Supplementary Fig. 6), and requiring efficient periplasmic 6S-GlcNAc desulfation is in line with this.

### *A. muciniphila* S1_16 sulfatases have specificity for O4 sulfated d-galactose

**Fig. 5:**
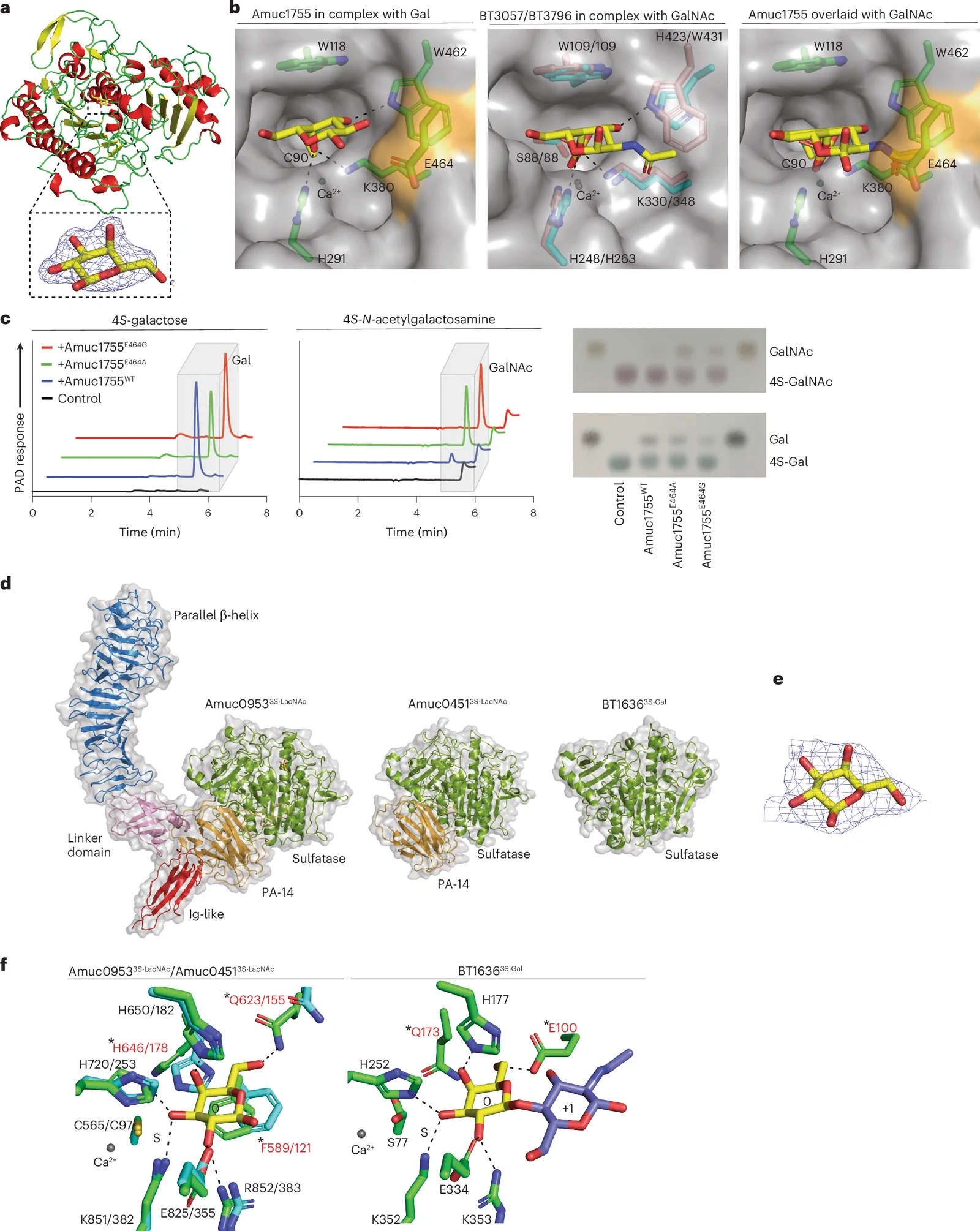
Structural features underpinning specificity in *A. muciniphila* S1_16 and S1_20 sulfatases. **a**, Cartoon representation of the structure of Amuc1755^4S-Gal^ in complex with d-galactose with the 2mFobs-DFc electron density map of d-galactose shown below contorted to 1*σ*. **b**, Crystal structure of the glycan-binding site of Amuc1755^4S-Gal^ compared with *B. thetaiotaomicron* S1_16 sulfatases that target 4S-Gal and 4S-GalNAc. **c**, HPAEC (left) and TLC analysis (right) of wild-type and mutant forms of Amuc1755^4S-Gal^ targeting 4S-Gal and 4S-GalNAc. It can be seen that E464 is critical for driving 4S-Gal specificity. **d**, Cartoon representation with a transparent surface of *A. muciniphila* and *B. thetaiotaomicron* S1_20 homologues. **e**, 2mFobs-DFc electron density map of d-galactose from the Amuc0953^3S-Lac/LNB^ complex contorted to 1*σ*. **f**, Stick representation of the glycan-binding sites of Amuc0953^3S-LacNac/LNB^, Amuc0451^3S-LacNac/LNB^ and BT1636^3S-Gal^. The galactose in the left panel is from the Amuc0953^3S-LacNac/LNB^ structure and Amuc0451^3S-LacNac/LNB^ has been superimposed. Source data

**Extended Data Fig. 7:**
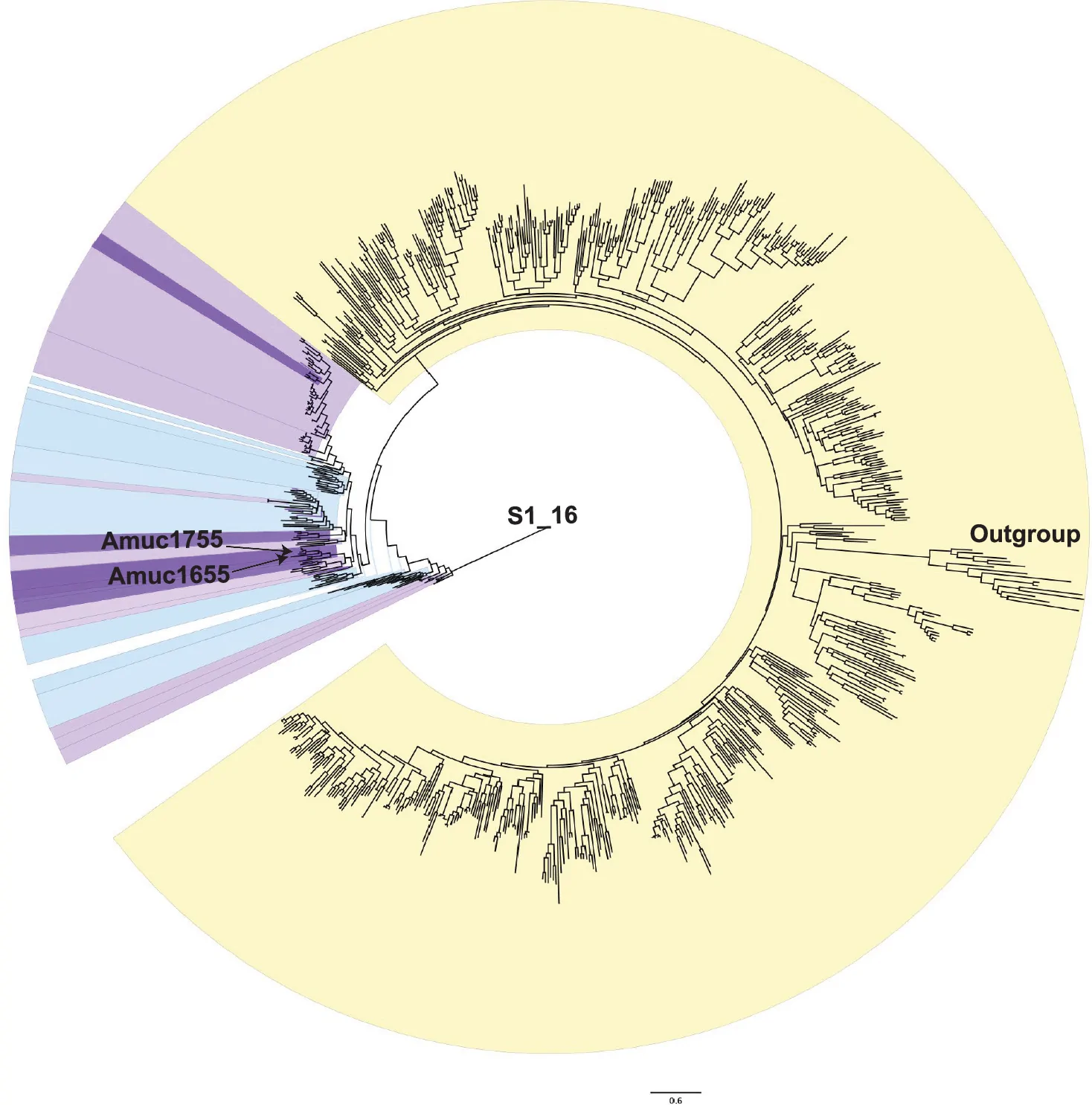
Phylogenetic tree of the S1_16 subfamily. Phylogenetic tree of 815 selected, most representative, sequences belonging to S1_16. Human and animal microbiome-associated bacteria are coloured in light purple (mainly gut, faeces, and oral cavity), the dark purple are exclusively gut bacteria and are those sequences carrying the W_GEX stretch containing E464 (in half of the sequences X is R or K, in one quarter is G, and all other sequences either S,T,Q,L or A); light blue are aqueous environmental bacteria (that is waste water, rivulet, mangrove, Chinese sea sediment, etc). Notably, three sequences among these aquatic environments possess the W_GEX pattern (purple dots). All other sequences, coloured in yellow, are bacteria isolated from various environmental, mainly soil samples.

Amuc1655^4S-Gal^ and Amuc1755^4S-Gal^ are monomeric (Fig. 5a and Supplementary Fig. 5a) and show a strong preference for 4S-Gal over 4S-GalNAc (Figs. 2a and 5c), in contrast to the characterized *B. thetaiotaomicron* S1_16 enzymes, which have equal activity on both^27^. The structure of Amuc1755^4S-Gal^ revealed that it had the key specificity residues identified in microbiota S1_16 enzymes for 4S-Gal/GalNAc recognition (Fig. 5b). Trp118 stacks against the alpha face of the sugar, and Trp462 interacts with O3 via the secondary amine of its indole ring. A key difference is the presence of Glu464, which resides on a loop in the motif W_GEX and interacts with O2 and limits the accommodation of an N-acetyl group, thus driving specificity for 4S-Gal (Fig. 5b); this residue is also present in Amuc1655^4S-Gal^. Analysis of S1_16 sequence alignments reveals that the W_GEX motif is rare and mainly restricted to the microbiota; only 25 of 800 analysed sequences contained this motif (Extended Data Fig. 7). Mutation of Glu464 to Ala and Gly enabled the Amuc1755^4S-Gal^ to work on 4S-GalNAc, but reduced activity on 4S-Gal (Fig. 5c and Supplementary Table 1). The low activity of the wild-type enzymes on 4S-GalNAc (Fig. 5c) suggests limited flexibility of the W_GEX loop.

### *A. muciniphila* multidomain S1_20 sulfatases targeting 3S-LacNAc

**Extended Data Fig. 8:**
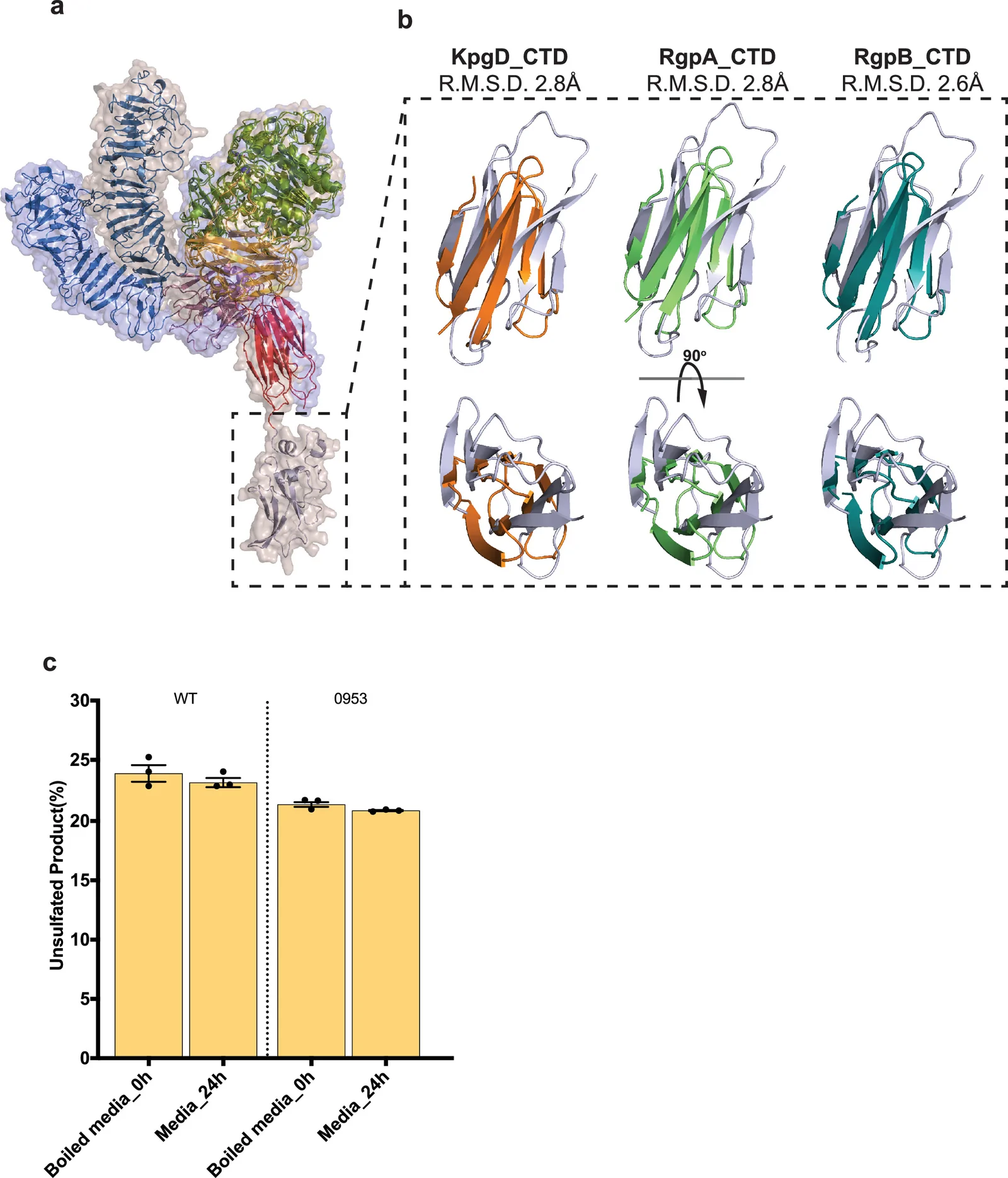
Comparison of the Amuc0953^3S-LacNAc/LNB^ crystal structure with its AF3 model, and 3S-Gal activity in the media. **a**. Overlay of the crystal structure of Amuc0953^3S-LacNAc/LNB^ and its AF3 predicted model **b**. Comparison of the C-terminal domain (CTD) of Amuc0953^3S-LacNAc/LNB^ with the CTD from type IX secretion systems. **c**. Filtered media from *A. muciniphila* cells grown on soluble porcine gastric mucin to mid-exponential phase was tested for its ability to desulfate 3S-Gal. Data are derived from 3 biological replicates with the standard error of the mean shown as error bars. Source data

Amuc0451^3S-LacNAc/LNB^ and Amuc0953^3S-LacNAc/LNB^ are large, monomeric, multidomain sulfatases (Fig. 5d) that show distinct unfolding stages in their melt curves (Supplementary Fig. 5a,b). To understand the significance of their size differences, and specificity, we solved crystal structures of both (Fig. 5d,e). Amuc0451^3S-LacNAc/LNB^ comprises the core S1, α/β/α fold, but with an abutted Ig-like PA14 domain at its C terminus. Amuc0953^3S-LacNAc/LNB^ also has an Ig-like PA14 domain C-terminal abutted to the core sulfatase domain, but has two further, smaller, domains; one of which is an Ig-like domain and the other was unresolved. At the N terminus, Amuc0953^3S-LacNAc/LNB^ possesses a large parallel β-helix domain connected to the core sulfatase domain via a small β-sheet domain with a single α-helix (Fig. 5d). AF3 structure prediction of Amuc0953^3S-LacNAc/LNB^ indicates that the final C-terminal domain (CTD) bears resemblance to the CTD recognized by Bacteroidota type IX secretion systems^33^. A comparable system may therefore exist in *A. muciniphila* enabling translocation of Amuc0953^3S-LacNAc/LNB^ to the cell surface (Extended Data Fig. 8a,b and Supplementary Table 3). No 3S-Gal activity could be detected in the media indicating that Amuc0953^3S-LacNAc/LNB^ is membrane bound at the cell surface (Extended Data Fig. 8c).

**Extended Data Fig. 9:**
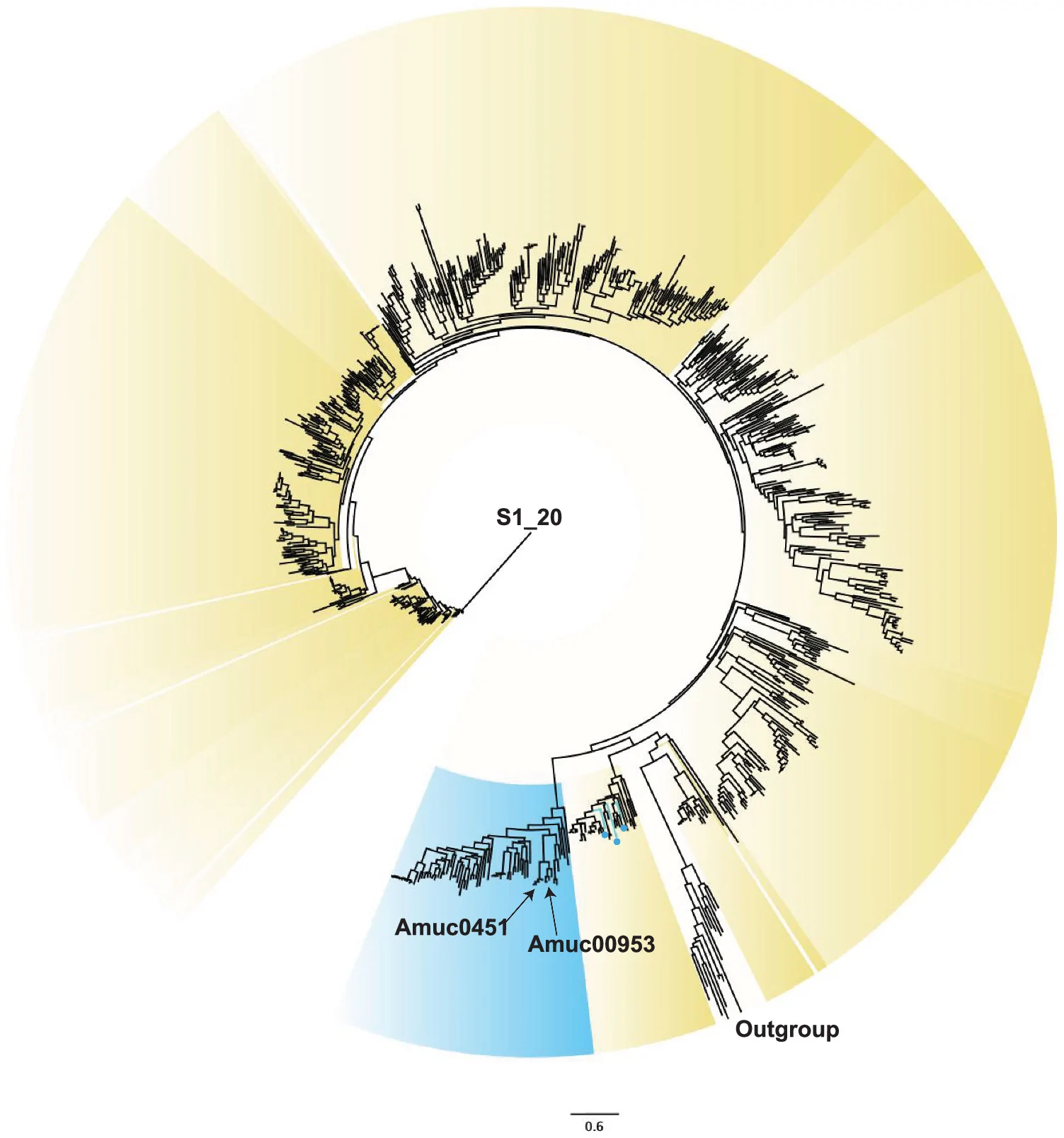
Phylogenetic tree of the S1_20 subfamily. Phylogenetic tree of 848 selected, most representative, sequences belonging to S1_20. The sequences carrying the concomitant Gln/His & Glu/Phe mutations are highlighted. Three sequences from *Streptomyces sp*. among the neighbouring group, all belonging to the Actinomycetota phylum, also contain the Gln/His & Glu/Phe mutations and are marked by blue lines and dots. The sequences of the branch highlighted in blue all belong either to the Bacteroidota, Planctomycetota or Verrucomicrobiota phyla.

Amuc0451^3S-LacNAc/LNB^ and Amuc0953^3S-LacNAc/LNB^ have identical binding site sequences and are similar to that of the critical *B. thetaiotaomicron* enzyme BT1636^3S-Gal^ (Fig. 5f). Histidine coordinates the Gal axial O4, while O2 is coordinated by Glu and Arg, and O6 by a Gln. Amuc0451^3S-LacNAc/LNB^ and Amuc0953^3S-LacNAc/LNB^ both have a Phe in the glycan-binding site that stacks against the Gal sugar ring, replacing Glu100 in BT1636^3S-Gal^. The addition of an interacting Phe residue is accompanied by a Gln to His (replacing Q173 in BT1636^3S-Gal^) covariant mutation, with the His lying perpendicular to Phe, which occurs only in ~9% of S1_20 sequences that cluster together (Extended Data Fig. 9).

### The N-terminal domain of Amuc0953 binds colonic mucin

Parallel β-helix domains are common for CAZymes targeting plant glycans^34^ and glycosaminoglycans^35^, as well as carbohydrate-binding module family (CBM) 89 which binds xylan^36^. However, no enzymatic activity for the N-terminal domain (NTD) of Amuc0953 could be observed against pectins, xylans, glycosaminoglycans or porcine gastic mucin, nor binding to xylan (Supplementary Fig. 7a,b). Therefore, we hypothesized that the Amuc0953 N-terminal parallel β-helix domain could be a mucin-binding domain.

**Fig. 6:**
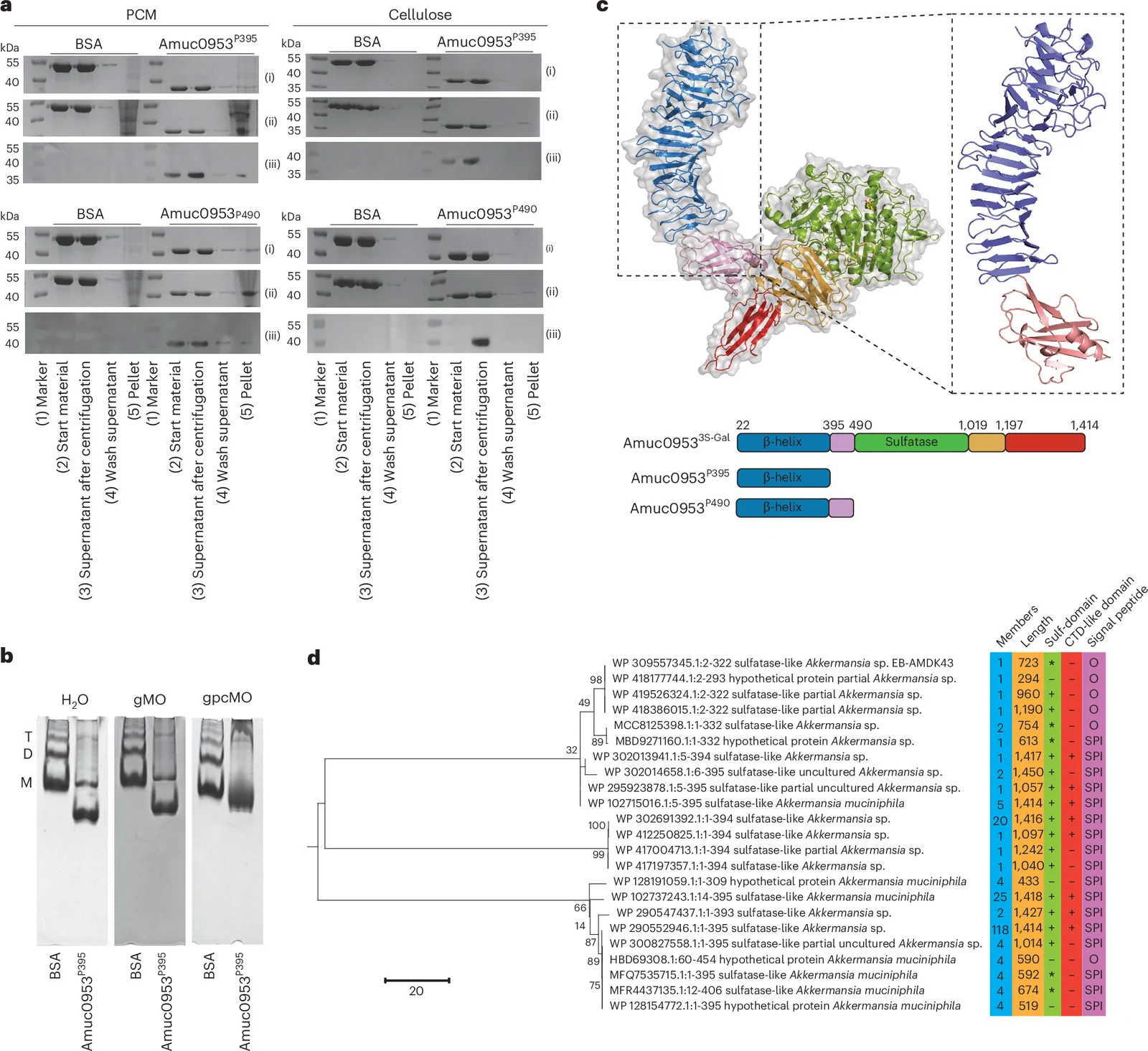
The N terminus of Amuc0953^3S-LacNAc/LNB^ is a mucin-binding module. **a**, Pull-down assays showing that Amuc0953^P395^ and Amuc0953^P490^ bind PCM. (i) SDS–PAGE with 10 μl of pull-down sample loaded; (ii) as in (i), but lane 4 has been concentrated 10-fold, (iii) western blot of (i) using an anti-His antibody: (1) marker, (2) start material: 10 mg ml^−1^ glycan plus 10 μM protein, (3) supernatant after centrifugation, (4) supernatant from wash; resuspension of pellet in PBS then centrifugation, (5) pellet; resuspension of pellet in PBS then boiled. Pull-down gels shown are representative gels from two independent experiments. **b**, Affinity gel electrophoresis of Amuc0953^P395^, run using 7.5% acrylamide gels with, and without, a 0.08% glycan source in the gel. A total of 10 μg of protein was loaded with BSA run as a non-interacting control. M, D and T indicate the monomeric (~66 kDa), dimeric (~132 kDa) and tetrameric (~264 kDa) forms of BSA, respectively. Gels are from a single experiment. **c**, Cartoon representations with a transparent surface of Amuc0953^3S-Lac/LNB^ with the structure of Amuc0953^P490^, extending to P395, identified in blue and the linker domain, extending to amino acid Pro490, shown in salmon. **d**, Unrooted maximum-likelihood tree constructed in MEGA X based on the alignment of amino acid sequences from BLAST using the ClusteredNR database. Branch lengths are proportional to the number of substitutions per site. The blue box indicates the number of members per clustered sequence, and the orange box indicates the sequence length. The green box indicates the presence of the S1_20 sulfatase-like domain in AlphaFold3 models generated from the sequence clusters, and the red box corresponds to the presence of a CTD-like structure. The purple box indicates whether there is a signal peptide present within the sequence as determined by Signal 6.0; ‘SPI’ indicates translocation through the Sec system to the periplasm, while ‘O’ indicates a result returned as ‘other’. Plus and minus symbols indicate whether a domain is present or absent, and an asterisk is indicative of the partial or incomplete sulfatase domain. Sequence names and identifiers have been truncated for clarity. Source data

We generated two constructs terminating at Pro395 (Amuc0953^P395^) and Pro490 (Amuc0953^P490^) and conducted pull-down assays using PCM. Both constructs were pulled down by these colonic mucins, while bovine serum albumin (BSA) was not, and neither protein interacted with cellulose (Fig. 6a). Affinity gel electrophoresis of Amuc0953^P395^ showed a marked interaction with gpcMOs and not gMOs, relative to the no-glycan control (Fig. 6b). Amuc0953^P490^ was successfully crystallized to 2.0 Å (Fig. 6c), and this model was used to search for structural matches in the PDB using Foldseek and PDBeFold. No significant matches were found using default values, but the top ‘next best’ matches from PDBeFold were two adhesin proteins from *Candida glabrata* (PDB: 7O9Q, 7O9P), major tropism determinant P1 (PDB: 2IOU), PBMDCECB_09513_CBM89 (PDB: 7JVI) and ABC transporter complex NosDFYL (PDB: 7OSH). Comparison with TM-align and JCE gave root mean square deviations of 3.95–5.21 Å, over ~180–200 aligned Cα residues providing no evidence of an evolutionary relationship (Supplementary Fig. 8).

A Blastp^37^ and PSI-Blast^38^ search returned only 206 sequences, of which 201 had a minimum identity of 40% with a 70% query coverage (Fig. 6d). All sequences were from the genus *Akkermansia* and most were from *A. muciniphila* strains, suggesting that it is a rare sequence restricted largely to *Akkermansia*. Over 90% of these sequences are associated with a sulfatase domain. Collectively, these data indicate that the NTD of Amuc0953^3S-LacNAc/LNB^ is a mucin-binding domain within the *Akkermansia* genus.

### Growth of carbohydrate sulfatase transposon mutants on mucin substrates

**Extended Data Fig. 10:**
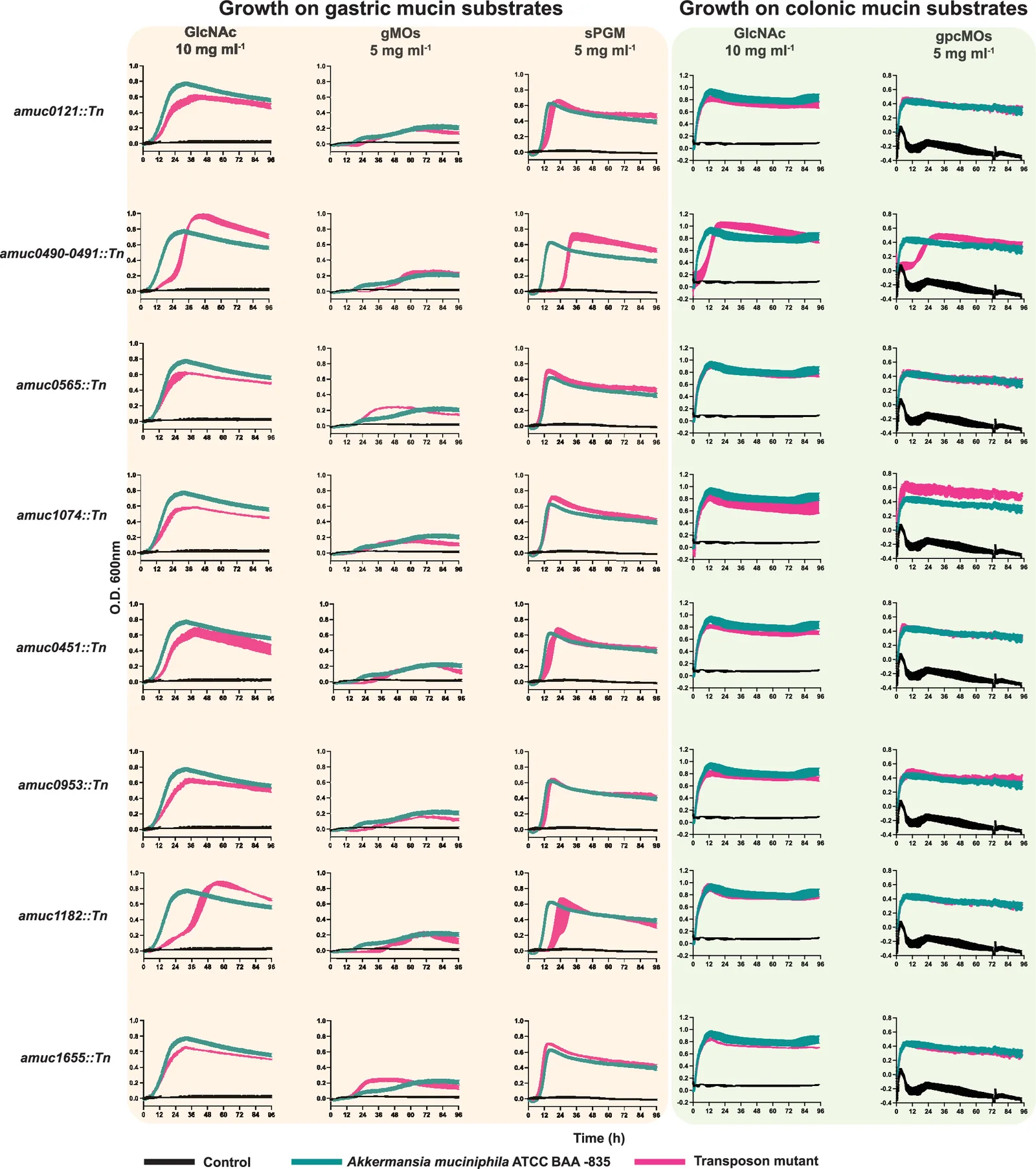
Growth of transposon mutants on mucin substrates. Bacterial growth curves of *Akkermansia muciniphila* ATCC BAA-835 and sulfatase transposon mutants on gastric and colonic mucin derived substrates. All growth curves were technical triplicates performed in CMM as a base media supplemented with 5 mg ml^−1^ of the appropriate carbon source as listed. GlcNAc = *N*-acetyl D-glucosamine, gMOs = gastric mucin oligosaccharides, sPGM = soluble porcine gastric mucin, and gpcMOs = glycoprotein porcine colonic mucin oligosaccharides. Source data

Having determined the catalytic specificities and cellular location of 8 of the 10 carbohydrate sulfatases, we examined the effect of the 8 transposon mutants (*amuc0121*::Tn, *amuc0451*::Tn, *amuc0565*::Tn, *amuc0953*::Tn, *amuc1074*::Tn, *amuc1182*::Tn, *amuc1655*::Tn and *amuc0490-491*::Tn) on the ability of *A. muciniphila* to use mucin substrates as a sole carbon source. Minor growth and variable differences were observed for all mutants grown on GlcNAc and gMOs (Extended Data Fig. 10). On sPGM and gpcMOs, most mutants showed no growth differences compared with the wild type except *amuc0490-491*::Tn and *amuc1182*::Tn. The mutant *amuc1182*::Tn had an increased lag phase only on sPGM, while the *amuc0490-491*::Tn mutant showed increased lag on all substrates including GlcNAc. The Tn insertion in *amuc0490-491*::Tn spans both genes, and the defect observed here is likely to be linked to *amuc0490* (a putative inner membrane protein of unknown function) and is a global defect not specific to mucin or the sulfatase Amuc0490^3S-Gal/GalNAc^. No activity for Amuc1182^S1_19^ could be detected, but the phenotype shown by *amuc1182*::Tn suggests that the protein could have a role in sPGM metabolism (Extended Data Fig. 10).

## Discussion

*A. muciniphila* can use gMOs and mucins, but fails to grow on cMOs. Recently, however, it has been shown that *A. muciniphila* can grow on trypsin-treated colonic mucin, suggesting that this bacterium requires the protein backbone to use these complex glycans^29^. Our growth data validate this finding, but indicate that *A. muciniphila* requires glycopeptides or glycoproteins of a specific size and/or motif to support strong growth on colonic mucin. This indicates that *A. muciniphila* is unable to complete the digestion of this trypsin-treated material to its preferred substrate and may require microbiota-driven processing to make them accessible. A recent study showed that *A. muciniphila* transports large mucin portions into its periplasm where they are degraded in a ‘mucinosome’ complex^39^. Our data are consistent with the idea of the transport of glycoproteins and glycopeptides across the membrane and periplasmic degradation in which mucin O-glycans are broken down to monosaccharides.

The sulfation of colonic mucin presents a steric hindrance to microbiota glycoside hydrolases. Although sulfoglycosidases from the GH20 family can cleave 6S-GlcNAc^40^, this sulfated monosaccharide must still be desulfated before being used in central metabolism. Desulfation of O3, O4 and O6 of Gal occurs at both the cell surface and periplasm in *A. muciniphila*, while desulfation of 6S-GlcNAc is performed exclusively in the periplasm. The surface Gal desulfation could render these residues accessible to microbiota galactosidases while maintaining 6S-GlcNAc as a bottleneck. In *B. thetaiotaomicron*, the cell surface S1_20 sulfatase, BT1636^3S-Gal^, is essential for efficient cMO utilization. By contrast, neither of the two homologues in *A. muciniphila* were found to be essential for *A. muciniphila* growth on gpcMOs. Although key enzymes located at the cell surface have been described as essential to the utilization of glycans by *Bacteroides*, it remains unclear whether *A. muciniphila* also has such critical enzymes.

One of the most interesting features of the cell surface Amuc0953^3S-LacNAc/LNB^ is the NTD, which is a mucin-binding domain that is exclusively found in the *Akkermansia* genus. These may fulfil a similar role to surface glycan-binding proteins observed in *Bacteroides* species for glycan capture^41^, increasing effective substrate concentration for the enzyme and helping Amuc0953^3S-LacNAc/LNB^ desulfate terminal 3S-Gal moieties.

The apparent rare specificity for Gal over GalNAc shown by the S1_16 enzymes suggests that 4S-Gal, detected in salivary mucins^42^, which transit the colon as part of the digestive process, is a motif targeted by *A. muciniphila*. The specificity of the *A. muciniphila* S1_16 sulfatases may provide it a competitive edge on this substrate. The S1_11 enzymes in *A. muciniphila* that target 6S-GlcNAc have clearly distinct activities driven by differences in the flexibility of a key N-acetyl loop. The reason why *A. muciniphila* has an apparently low-efficiency 6S-GlcNAc sulfatase is unclear, but it is possible that this enzyme targets larger 6S-GlcNAc-containing glycans, or glycopeptides.

For both gastric and colonic mucin, *A. muciniphila* requires a glycoprotein and glycopeptide signature that is not required by other Gram-negative mucin degraders such as *B. thetaiotaomicron*, indicating a significant difference in acquisition modes. Furthermore, Gal desulfation occurs both outside and inside the cell, while desulfation of GlcNAc is exclusively periplasmic, and a more selfish pursuit. This is consistent with GlcNAc being a favoured monosaccharide carbon source^32^. Overall, this illustrates distinct colonic mucin metabolism strategies, involving carbohydrate sulfatases, by *A. muciniphila* and *B. thetaiotaomicron* and begins to shed light on their differing mucin metabolism during symbiosis and dysbiosis.

## Methods

### Chemicals and reagents

All sulfated carbohydrates were purchased from Dextra, except O3 sulfated d-galactose that was purchased from Toronto Research Chemicals. Porcine gastric mucin type III (M1778-100G) was obtained from Sigma.

### Recombinant protein production

Genes were amplified by PCR using the appropriate primers (Supplementary Table 4) and cloned into pETite with *N*-His_6_ using the the Expresso T7 cloning and Expression System kit (Lucigen). Recombinant genes were expressed in *E. coli* strains TUNER (DE3) (Novagen) and cultured to mid-exponential phase in LB supplemented with 50 μg ml^−1^ kanamycin at 37 °C and 180 rpm. Cells were then cooled to 16 °C, and recombinant gene expression was induced by the addition of 0.1 mM or 0.2 mM isopropyl β-d-1-thiogalactopyranoside (IPTG); cells were cultured for another 16 h at 16 °C and 180 rpm. The cells were then centrifuged at 5,000*g* and resuspended in 20 mM 4-(2-hydroxyethyl)-1-piperazineethanesulfonic acid (HEPES), pH 7.4, with 500 mM NaCl before being sonicated on ice. Recombinant protein was then purified by immobilized metal ion affinity chromatography (IMAC) using a cobalt-based matrix (Talon, Clontech) and, after a wash with resuspension buffer, eluted with a step gradient of 10 mM, 50 mM and 100 mM imidazole in resuspension buffer. Proteins were then analysed by SDS–PAGE gel for purity and appropriately pure fractions dialysed into 10 mM HEPES, pH 7.0, with 150 mM NaCl. Proteins being carried forward for structural experiments were concentrated in centrifugal concentrators with a molecular mass cut-off of 30 kDa, and loaded onto a 16/60 S200 Superdex size-exclusion column (Cytiva). Fractions from this were then subjected to SDS–PAGE analysis, and fractions judged to be >95% pure were pooled and concentrated in centrifugal concentrators with a molecular mass cut-off of 30 kDa. Protein concentrations were determined by measuring absorbance at 280 nm using the molar extinction coefficient calculated by ProtParam on the ExPasy server (https://web.expasy.org/protparam/).

### Site-directed mutagenesis

Site-directed mutagenesis was conducted using a modified PCR-based QuikChange protocol. Appropriate primer pairs were designed with the mutation in the middle region and overhangs overlapping the gene sequence of 12 to 20 nucleotides (Supplementary Table 5). The plasmid was amplified by PCR using CloneAmp HiFi PCR Premix (Takara Bio) or Phusion High-fidelity DNA polymerase (Thermo Scientific). Alternatively, mutants were generated using primers that generated blunt-ended products with the mutation at the 5′ terminus that were ligated together. A 0.8% agarose gel was run to visualize the PCRs, and successful reactions were digested with Dpn I. The digestion reaction (2–5 μl) was transformed into 100 μl of Top10 super-competent cells. After recovery, the cells were plated onto LB agar plates containing kanamycin (50 μg ml^−1^) and grown overnight at 37 °C. Colonies were picked and grown in LB supplemented with 50 μg ml^−1^ kanamycin, and the plasmid DNA was isolated and sequenced to confirm the correct sequence with the mutation.

### Bacterial growth assays

*A. muciniphila* ATCC BAA-835 was grown in 5 ml of chopped meat medium (Supplementary Table 6) supplemented with an appropriate carbon source. After recovery, cells were subcultured twice in chopped meat medium with 10 mg ml^−1^ GlcNAc before the experiment was set up. *B. thetaiotaomicron* VPI-5482 was recovered in brain–heart infusion media supplemented with haematin. The next day, cultures were inoculated in minimal media (Supplementary Table 7) with an appropriate carbon source for the experiment. Media glycan solutions were made as 2× stocks and mixed to make 1× solutions for growth experiments. Growth was carried out at 37 °C inside a Don Whitley A85 anaerobic workstation filled with a mixture of 10% CO_2_, 10% hydrogen, and 80% nitrogen.

### Enzymatic assays analysed by liquid chromatography with electrospray ionization tandem mass spectrometry

Assays were performed with 1 μM of recombinant enzymes and 0.5% colonic mucin glycans in 10 mM MES, pH 6.5, with 5 mM CaCl_2_ for 16 h at 37 °C. The resultant glycans were purified by passing through graphitized carbon particles (Thermo Scientific) packed on top of a C18 ZipTip (Millipore). Samples were eluted with 65% acetonitrile in 0.5% trifluoroacetic acid (v/v), dried and stored at −20 °C until liquid chromatography with electrospray ionization tandem mass spectrometry (LC–ESI MS/MS) analysis. Released glycans were resuspended in 15 μl of Milli-Q water and analysed by LC–ESI MS/MS using a 10 cm × 250 µm ID column, packed with 5 µm porous graphitized carbon particles (Hypercarb, Thermo-Hypersil). Glycans were eluted using a linear gradient of 0–40% acetonitrile in 10 mM NH_4_HCO_3_ over 40 min at a flow rate of 6 µl min^−1^. The eluted O-glycans were detected using an LTQ mass spectrometer (Thermo Scientific) in negative-ion mode with an electrospray voltage of 3.5 kV, a capillary voltage of −33.0 V and a capillary temperature of 300 °C. Air was used as a sheath gas. A full scan (m/z 380–2,000, 2 microscans, maximum 100 ms, target value of 30,000) was performed, followed by data-dependent MS2 scans (2 microscans, maximum 100 ms, target value of 10,000) with normalized collision energy of 35%, isolation window of 2.5 units, activation *ρ* = 0.25 and activation time of 30 ms. The threshold for MS2 was set to 300 counts. The data were processed using Xcalibur software (version 2.0.7, Thermo Scientific). Glycans were identified from their MS/MS spectra by manual annotation as previously described^4^. Raw data were uploaded on Glycopost (https://glycopost.glycosmos.org/preview/3719534668b934377a9c6, password: 7654). The peak area (the area under the curve, AUC) of each glycan structure was calculated using Progenesis QI software (Nonlinear Dynamics, Waters). The AUC of each structure was normalized to the total AUC and expressed as a percentage.

### Size-exclusion coupled light scattering determination of molecular weight

Molecular masses were determined using an Agilent Multi-Detector System calibrated with bovine serum albumin. Proteins were separated by size-exclusion chromatography using an Agilent BioSEC Advance 300 Å, 4.6 × 300 mm column equilibrated with 20 mM tris(hydroxymethyl)aminomethane-HCl, pH 7.4, and 150 mM NaCl buffer. Light scattering data were collected at 90° and the refractive index used to calculate absolute molecular mass.

### Differential scanning fluorimetry

Thermal stability shift assays (TSAs) were performed using a Stratagene Mx3005P Real-Time PCR machine (Agilent Technologies) and SYPRO-Orange dye, at a 1:1,000 dilution, (emission maximum, 570 nm; Invitrogen) with thermal ramping between 20 °C and 95 °C in 0.3 °C-step intervals every 15 s per data point to induce denaturation. The melting temperature (*T*_m_) corresponding to the midpoint for the protein unfolding transition was calculated by fitting the sigmoidal melt curve to the Boltzmann equation using GraphPad Prism, with *R*^2^ values ≥0.99, as previously described^43^. Data points after the fluorescence intensity maximum were excluded from the fitting. Changes in the unfolding transition temperature compared with the control curve (Δ*T*_m_) were calculated for each ligand. A positive Δ*T*_m_ value indicates that the ligand stabilizes the protein from thermal denaturation and confirms binding to the protein. All thermal shift assay experiments were conducted using a final protein concentration of 5 μM in 100 mM Bis-Tris-Propane or HEPES, pH 7.0, and 150 mM NaCl, supplemented with the appropriate ligand concentration.

### X-ray crystallography experiments

Sparse matrix screens were set up in 96-well sitting drop TTP Labtech plates (400-nl drops) using an SPT mosquito crystallization robot, or in sitting drop Intelli-Plates (400-nl drops) using an Arts Robbins gryphon robot. All proteins were in 10 mM HEPES, pH 7.5, and 150 mM NaCl. Amuc0451 crystallized in 20% PEG 3350; 0.1 M Bis-Tris-Propane, pH 7.5; and 0.2 M sodium–potassium phosphate at 40 mg ml^−1^ with 100 mM Gal. Amuc0953 crystallized in 20% PEG 3350 and 0.2 M sodium iodide at 60 mg ml^−1^ with 100 mM Gal. Amuc1074 crystallized in the Morpheus screen condition E9; 37.5% precipitant mix 4; 0.1 M buffer system 2, pH 7.5; and 0.12 ethylene glycol mix at 25 mg ml^−1^ with 100 mM GlcNAc. Amuc1755 crystallized in 1.6 M sodium citrate at 2.75 mg ml^−1^ with 100 mM Gal. Amuc0121 at 36 mg ml^−1^ with 100 mM Gal crystallized in the Morpheus condition H12; 37.5% precipitant mix 4; 0.1 M buffer system 3, pH 8.5; and 0.1 M amino acid mix. Amuc0121 at 36 mg ml^−1^ with 10 mM 6S-Gal crystallized in Morpheus condition D12; 37.5% precipitant mix 4; 0.1 M buffer system 3, pH 8.5; and 0.1 M alcohol mix. Amuc0121 at 36 mg ml^−1^ with 1 mM 6S-Lewis A crystallized in Morpheus condition B1; 30% precipitant mix 1; 0.1 M buffer system 1, pH 6.5; and 0.09 M halogen mix. These crystals were soaked for 24 h with 5 mM 6S-Lewis A that had been dissolved in the crystallization condition. Amuc0953^P490^ was crystallized in 2.0 M ammonium sulfate and 0.1 M sodium acetate 4.6 at 20 mg ml^−1^; crystals were soaked with 10 mg ml^−1^ gpcMOs overnight before fishing; no cryoprotection was used. Crystals from Morpheus conditions do not need additional cryoprotection while all other crystals were cryo-cooled with the addition of 20% PEG 400, ethylene glycol or glycerol, except Amuc1755, which was cryo-protected with 4.5 M sodium citrate. Data were collected at Diamond Light Source (Oxford) on beamlines I0-3, I0-4 and I24 at 100 K. The data were integrated and scaled with auto processing software at Diamond via xia2 or autoPROC with STARANISO pipelines and merged with Aimless^44,45^. For the *R*_free_ set, 5% of observations were randomly selected and not subject to refinement as to monitor model quality during refinement. The phase problem was solved by molecular replacement using the program Phaser or Molrep with a model generated through the AF2 colab or the AF3 server. Models then underwent recursive cycles of model building in Coot^46^ and refinement cycles in Refmac5^47^. The models were validated using Coot^46^ and MolProbity^48^. Carbohydrates were made using Jigand^49^. Structural figures were made using Pymol (The PyMOL Molecular Graphics System, Version 2.0 Schrodinger, LLC), and all other programs used were from the CCP4 suite^50^. The data processing and refinement statistics are reported in Supplementary Tables 8 and 9).

### Molecular dynamic simulations

All-atom molecular dynamic simulations were performed in GROMACS 2023 using the Charmm36m force field^51^. The explicit solvent systems were set up using CHARMM-GUI (refs. ^52,53^). Amuc1033 (AlphaFold2 (ref. ^54^)) and Amuc1074 (PDB: 9S8Y) were solvated in water containing 150 mM NaCl, and the protonation states of titratable residues were set at pH 7.5. Each system was energy minimized and equilibrated with position restraints at 310.15 K temperature and 1 bar pressure for 5 ns. Systems were then subjected to an unrestrained production run for 100 ns each. The temperature and pressure were maintained by the V-rescale thermostat^55^ and C-rescale barostat^56^. Analysis was performed by in-built Gromacs analysis tools, UCSF Chimera and GraphPad Prism.

### Thin-layer chromatography

End-point assays were analysed by thin-layer chromatography (TLC) by spotting 2 μl of sample onto aluminium-backed silica plates and resolved in a butanol:acetic acid:water (2:1:1) solvent or formic acid:butanol:water (4:8:1) system. The plates were dried, and the sugars were visualized using diphenylamine stain (1 ml of 37.5% HCl, 2 ml of aniline, 10 ml of 85% H_3_PO_3_, 100 ml of ethyl acetate and 2 g diphenylamine) and heated at 450 °C for 2–5 min with a heat gun.

### Glycan labelling

Sulfated saccharide samples were labelled according to a modification of the method, reporting that the formation of *N*-glycosyl amines for 4,6-*O*-benzilidene protected d-gluopyranose monosaccharides with aromatic amines^57^. In brief, lyophilized sugar (1 mg) was dissolved in anhydrous methanol (0.50 ml, Sigma-Aldrich) in a 1.5-ml screw-top PTFE microcentrifuge tube and BODIPY-FL hydrazide (4,4-difluoro-5,7-dimethyl-4-bora-3a,4a-diaza-*s*-indacene-3-propionic acid, hydrazide, 0.1 mg, Thermo Fisher, *λ*_ex./em._ 493/503, extinction coefficient (*ε*) 80,000 M^−1^ cm^−1^) was added and the mixture vortexed (1 min), then reacted (65 °C, 48 h) in darkness. The products were then cooled, and a portion purified by TLC on silica-coated aluminium plates (silica gel 60, Sigma-Aldrich, Millipore) resolved with butanol:acetic acid:water (2:1:1). Products were then separated from unreacted material down a silica column equilibrated with ethyl acetate with the unlabelled material, but not the labelled sulfated saccharides, able to run down the column. The labelled product is then eluted off the silica column with methanol and dried (rotary evaporator) to afford the fluorescent, coloured product (bright green in aqueous solution).

### Microfluidic desulfation assays

Sulfated carbohydrates labelled with BODIPY-FL, which has a maximal emission absorbance of ∼503 nm, can be detected via LED-induced fluorescence^43^. Non-radioactive microfluidic mobility shift carbohydrate sulfation assays were optimized in solution with a 12-sipper chip coated with CR8 reagent and a PerkinElmer EZ Reader II system using ethylenediaminetetraacetic acid (EDTA)-based separation buffer and real-time kinetic evaluation of substrate desulfation. Pressure and voltage settings were adjusted manually (1.8 psi; upstream voltage, 2,250 V; downstream voltage, 500 V) to afford optimal separation with a sample (sip) time of 0.2 s. Individual desulfation assays were carried out at 28 °C and assembled in a 384-well plate in a volume of 80 μl in the presence of substrate concentrations between 0.5 μM and 20 μM with 100 mM Bis-Tris-Propane, MES or Tris, depending on the pH required, 150 mM NaCl, 0.02% (v/v) Brij-35 and 5 mM CaCl_2_. The activity of sulfatase enzymes was quantified in ‘kinetic mode’ by monitoring the amount of unsulfated glycan generated over the assay time, relative to the control assay with no enzyme, with sulfate loss limited to ∼20% to prevent substrate depletion and to ensure assay linearity. *k*_cat_/*K*_m_ values, using the equation *V*_0_ = (*k*_cat_/*K*_m_)[*E*][*S*], were determined by linear regression analysis with GraphPad Prism software. Substrate concentrations were halved and doubled to assess linearity of the reaction rates to ensure substrate concentrations were <*K*_m_.

### Half-maximal inhibitory concentration assays

Amuc1033^6S-GlcNAc^ (2 μM) and Amuc1074^6S-GlcNAc^ (50 nM) were assayed against a dilution series of GlcNac (100 nM to 100 mM), using 1 μM BODIPY-labelled 6S-GlcNac as a fluorescent substrate. Progress of the reactions was measured by Thermo Scientific/Dionex ICS-6000 HPAEC with a Vanquish Fluorescence detector, with separation of sulfated substrate and unsulfated product over a Dionex CarboPac PA-200 3 × 50 mm BioLC guard column at a flow rate of 1 ml min^−1^ for 3 min. The mobile phase consisted of 100 mM sodium hydroxide with 0.2 M sodium acetate; from 0 to 0.25 min, the sodium acetate concentration was increased to 1 M, held until 2 min, after which the column was re-equilibrated back into 100 mM sodium hydroxide with 0.2 M sodium acetate for the remainder of the run. Sampling was carried out eight times across a 24-h period (Thermo Scientific/Dionex AS-AP autosampler), and relative peak area was assessed using Chromeleon 7. GraphPad Prism was used for subsequent data processing and for calculating half-maximal inhibitory concentration (IC_50_) values.

### Linked assays using 6*S*-GlcNAc β-d-methylumbelliferone

Kinetic parameters of Amuc1033 and Amuc1074 were monitored via a continuous linked assay using the substrate 4-methylumbelliferyl-GlcNAc6S (ChemCruz, sc-223640A). Desulfation of the substrate to 4-methylumbelliferyl-GlcNAc allows it to be hydrolysed by BT_3868^GH20^, releasing the fluorophore 4-methylumbelliferone (ex: 355 nm; em: 460 nm). BT_3868^GH20^-mediated hydrolysis is an indirect indicator of desulfation, monitored at 460 nm. A total of 10 substrate concentrations were tested between 0 and 10 mM in technical triplicate. Enzyme concentrations were 1 μM and 0.1 μM for Amuc1033 and Amuc1074, respectively. All assays were carried out in 10 mM HEPES, pH 7.5, and 150 mM NaCl. Data acquisition was performed using FLUOstar Omega (BMG LABTECH). The specific fluorescence intensity of 4-methylumbelliferone was calculated using a standard curve. 4-Methylumbelliferone release was plotted as a function of substrate concentration for the determination of *K*_m_ and *k*_cat_. Data analysis was performed using GraphPad Prism.

### High-performance anion-exchange chromatography

To monitor the liberation sugars from sulfated mono-, di- and trisaccharides, the enzyme variants at 5 μM were mixed 1:1 with 5–10 mM of the substrate and incubated overnight at 37 °C. These reaction mixtures were diluted 1:100 in 10 mM MOPs and 150 mM NaCl, pH 7, and analysed by HPAEC coupled to pulsed amperometric detection (PAD). HPAEC was performed using a Dionex CarboPac PA200 analytical (3 × 250 mm) and guard column (3 × 50 mm; Thermo Scientific). Samples were run for 20 min, with an isocratic flow of 0.1 M NaOH, before being run into 100% 1 M sodium acetate for 10 min, followed by 5 min of 0.5 M NaOH, before a final equilibration step of 0.1 M NaOH for 10 min. The flow rate was 0.5 ml min^−1^.

### Cell-based enzyme localization assays via HPAEC with fluorescent detection

Whole-cell assays were performed using *A. muciniphila* grown in *N*-acetylglucosamine (BG83, Dextra) and sPGM to an OD of 0.3–0.4. Cells were combined in a 1:1 ratio with 1 μM BODIPY-labelled sulfated monosaccharides. For the determination of relative localization of the sulfatases, both intact cells and CFEs were used. Control cells were heat deactivated at 95 °C for 10 min. Sulfatase activity was monitored via BODIPY fluorescence (ex: 502 nm; em: 522 nm) through periodic sampling over 4 h at 37 °C. All runs were carried out in a Dionex AS-AP HPLC system (Thermo Scientific) with a Dionex CarboPac PA200 BioLC 3 × 50 mm guard column (Thermo Scientific) equilibrated with 100 mM sodium hydroxide (S/4940/17, Fisher Scientific) and 0.2 M sodium acetate (S/2046/50, Fisher Scientific). Sulfated and desulfated monosaccharides were separated using a step gradient of sodium acetate (0.2–1 M) over 1.75 ml with a total run time of 3 min. Enzymatic activity was determined by plotting the relative peak area of the desulfated product as a function of time. Monosaccharides tested were 3S-Gal (TRC-G155295, LGC), 4S-Gal (G0012, Dextra), 6S-Gal (G0011, Dextra), 4S-GalNAc (G1054, Dextra) and 6S-GlcNAc (G1004, Dextra). All experiments were carried out in at least three biological replicates. For whole-cell assays to test the activity of Amuc1033, wild-type and *amuc1074*::Tn mutant cells were exposed to 5 mM of 6S-GlcNAc, and the experiments were conducted in biological duplicates. For all assays, an unpaired two-tailed *t*-test was used to determine statistical significance. Data analysis was performed using GraphPad Prism (v10.5.0).

### Two-colour fluorescence assay for bacterial viability

The LIVE/DEAD BacLight bacterial viability kit (Thermo Fisher Scientific) was used to determine the number of viable and nonviable *A. muciniphila* cells after incubation for 4 h in chopped meat broth or PBS pH 7.4. All bacterial cell-containing samples used in the viability assay had approximately the same cell density (OD_600_ of 0.3–0.4). A freshly prepared dye solution containing 10 µM SYTO-9 (dye stains all cells in the population) and 4 µM propidium iodide (dye stains only cells with a comprised membrane) was prepared in either chopped meat broth or PBS depending on the experimental condition. Equal volumes (50 µl) of this dye solution and the cell suspension were mixed, then incubated in the dark at room temperature (20 °C) for 15 min on a rotary wheel (12 rpm, 80° incline relative to bench top). After staining, the cell suspension was mixed again, and an aliquot (2 µl) of the stained cells was immediately pipetted onto a 1 cm × 1 cm agarose pad and covered with a clean number 1.5 coverslip (22 mm × 22 mm, Scientific Laboratory Supplies). Pads were prepared using low-melting-point UltraPure agarose (Invitrogen) and unsupplemented M9 medium (48 mM Na_2_HPO_4_, 22 mM KH_2_PO_4_, 8.6 mM NaCl, pH 7.2) according to a published protocol^58^. The cells were imaged by epifluorescence microscopy using an Axioskop 40 upright microscope (Zeiss) equipped with a ×100 objective (Achroplan NA 1.25, Zeiss), a CoolSNAP EZ camera (Photometrics), and excitation and emission filter sets for the dyes (SYTO-9: Zeiss filter set 10; propidium iodide: Zeiss filter set 43). If the cell density was low in a microscopy sample, the corresponding stained cell suspension was centrifuged (5,000*g* for 5 min at 20 °C) and a portion of the supernatant (~90 µl) was carefully removed. The cell pellet was resuspended in the remaining supernatant (~10 µl), and a fresh agarose pad-mounted sample was prepared for microscopy as above. Image stacks (35 ms exposure, 100 frames, no interval delay) were collected using MicroManager (version 1.4.16)^59^. The image stacks were averaged in FIJI (version 2.14)^60^, and the number of stained cells in each fluorescence channel was manually counted. The calculated number of viable cells for each condition was equal to the number of SYTO-9-stained cells minus the number of propidium iodine-stained cells. Three biological replicates (*n* = 3) were analysed for each experimental condition.

### Colonic mucin extraction

Frozen porcine colons stretching ~1 m in length from the anus were thawed, cut open and washed with tap water before having the mucus scraped using the back edge of a blunt instrument. The scraped material was then either frozen or carried forwards into an extraction buffer, containing 6 M GuHCl, 5 mM EDTA and 0.01 M sodium dihydrogen phosphate at pH 6.5 that was 5–10 times the volume versus the mass of material, and homogenized with a blunt instrument. The material was stirred slowly for 1–2 h at room temperature before centrifugation at 15,000*g* for 30 min, the supernatant discarded and fresh, cold, extraction buffer added to the insoluble pellet. This process was repeated at least five times or until the last two extractions were clear. The extracted material was then solubilized in 0.5 l of reduction buffer, containing 6 M GuHCl, 5 mM EDTA and 0.1 M Tris at pH 8.0 with 25 mM DTT left of ~5 h at 37 °C. To this, 62.5 mM iodoacetamide was added and the mixture stirred slowly at room temperature in the dark overnight. The material was then centrifuged at 10,000*g* for 30 min at 4 °C and the insoluble material discarded. The supernatant containing solubilized mucins was extensively dialysed against MQ water at least six times before being lyophilized and the weight calculated.

### Protease treatment of colonic mucin

PCM (400–500 mg) was mixed with 30 ml of filtered Milli-Q water and incubated overnight at 37 °C with shaking at 120 rpm. The suspension was centrifuged (30,000*g*, 15 min), and the supernatant was discarded. The pellet was resuspended in 30 ml reaction buffer (50 mM Tris–HCl, pH 8.0; 1 mM CaCl_2_). Next, 100 μg of proteinase K–trypsin was added and incubated overnight at 37 °C with shaking at 120 rpm. Digested material was centrifuged (30,000*g*, 15 min) and the pellet was discarded. The supernatant was heated at 95 °C for 10 min for enzyme deactivation then dialysed extensively against water and finally filtered. The solution was then freeze-dried, and the final concentration was adjusted as per requirement. The PCM digests were kept at −20 °C.

### Release of O-glycans by β-elimination

Mucin (25 g) was added to 1 l of 100 mM Tris, pH 7.0, and the mixture was autoclaved and allowed to cool to 65 °C before 100 mg of proteinase K was added and incubated overnight at 65 °C. Insoluble material was removed by centrifugation at 10,000*g* for 30 min before adding 1 M NaBH_4_ and 0.4 M NaOH and left overnight at 65 °C. The mixture was then neutralized to pH 7.0 using HCl before centrifugation at 10,000*g* for 30 min and filtering through a 0.2-μm filter. The material was extensively dialysed (1 kDa cut-off) against water before being lyophilized and the weight calculated.

### NMR analysis

NMR spectra were recorded using a Bruker NEO 700-MHz spectrometer or a Bruker Avance II+ or 800-MHz Avance NEO spectrometer. Both were fitted with a 5-mm TCI nitrogen cryoprobe. Sample volumes were 600 μl in D_2_O and data were recorded at 298k. For experiments on the Bruker NEO 700-MHz spectrometer: 1D proton spectra were acquired with 16 scans, both with and without presaturation solvent suppression (Bruker sequence ZGPR). A 2D ^1^H–^13^C HSQC was also acquired (Bruker sequence hsqcedetgpsisp2.4). The sPGM spectra were collected with 8 scans and 1,024 increments in the indirect dimension. In the case of SPCM and GMO, 32 scans and 512 increments were used. For experiments on the Bruker Avance II+ or 800-MHz Avance NEO spectrometer: 1D proton spectra and 2D HSQC NMR experiments were performed on fractions solubilized in 700 μl D_2_O (5-mm NMR tubes). Chemical shifts were quoted relative to DSS (0 ppm) using zpgr (1D ^1^H) and hsqcetgpsisp (HSQC) pulse programmes. Spectra were processed using Bruker TopSpin 3.4 and 4.0 software.

### Glycoprotein gel staining

Glycoprotein gel staining was performed using the Pro-Q Emerald 300 Glycoprotein Gel and Blot Stain Kit (Molecular Probes, P21857). In brief, mucin and mucin oligosaccharides were separated in a 12.5% SDS–PAGE gel. After the run, the gel was subjected to fixation and a wash. The gel was then exposed to an oxidizing solution, followed by three washes. Finally, the gel was exposed to the staining buffer for 90 min, followed by 3 additional washes, and imaged at 300 nm using a UV transilluminator.

### Isothermal titration calorimetry

Isothermal titration calorimetry (ITC) was performed using a MicroCal Auto-iTC200 (Malvern). The protein sample (50 μM) was stirred at 750 rpm, and 400 μl of sample was added to the loading plate, alongside 120 μl of titrant. An initial injection (0.5 μl) of titrant was added before an additional 19 injections (2 μl). Titrations were carried out in 50 mM Tris–HCl buffer, pH 8.0, at 25 °C. Titrant-to-buffer, buffer-to-protein and buffer-to-buffer control experiments were performed. Data analysis was performed using MicroCal PEAQ-ITC Analysis software (Malvern).

### Pull-down binding assays

Protein (10 μM) was mixed with 10 mg ml^−1^ polysaccharide and incubated for 30 min in 10 mM HEPES, pH 7.0, and 150 mM NaCl. Samples were then centrifuged at 17,000*g*, the supernatant removed and the pellet washed and resuspended in 1 ml of buffer. Samples were then centrifuged at 17,000*g*, the supernatant removed and the pellet resuspended in either 1 ml or 0.1 ml of buffer as needed. A 10-μl sample was taken at each stage, and this ran on a 12.5% Tris–glycine SDS–PAGE gel and developed with Coomassie blue stain. Samples were also subjected to western blotting by being transferred to a nitrocellulose membrane, 0.45 μM. Membranes were initially blocked with 5% milk powder suspended in PBS for 1 h before being washed three times with PBS and 0.1% tween for 5 min each. Membranes were then blotted with a mouse anti-His antibody (Thermo Fisher MA1-21315), 1:5,000 dilution, in PBS with 5% milk powder overnight at 4 °C. Membranes were then washed three times with PBS and 0.1% tween for 5 min each and blotted with rabbit anti-mouse conjugated to HRP (Thermo Fisher 31450) and developed with Clarity Western ECL Substrate (BioRad 1705060).

### Affinity gel electrophoresis

Tris–glycine gels were cast containing 7.5% acrylamide using acrylamide–bisacrylamide solution (37.5:1), 9.4 mM Tris and 94 mM glycine, and supplemented with either water or 0.08% glycan as appropriate. The final concentration of the running buffer used was 25 mM Tris with 250 mM glycine; the pH of this solution was not adjusted and was made as a 10× stock. A total of 10 μg of protein was loaded per well, including a BSA non-interacting control on all gels using a loading dye of 20% glycerol and 0.05% bromophenol blue. Gels were run at 10 mA per gel for a period of up to 90 min.

### Phylogenetic and sequence analyses

Phylogenetic analyses were performed as in a previous study^4^. A representative number of sequences within each subfamily covering the taxonomic diversity was used: (1) for S1_11, 955 sequences were selected from the SulfAtlas database and 411 positions were used for phylogeny, (2) for S1_16, 800 sequences were selected from SulfAtlas and 342 positions were used for phylogeny and (3) for S1_20, 848 sequences present in SulfAtlas were selected, and 364 positions were used for phylogeny. In each case, the sequences were aligned by MAFFT v.7 (ref. ^61^) using the L-INS-i algorithm. The multiple sequence alignments were visualized by Jalview software v.11.0 (ref. ^62^), non-aligned regions were removed and the above-listed respective numbers of positions were used for the phylogeny. Phylogeny was made using RAxML v. 8.2.4 (ref. ^63^). The phylogenetic trees were built as previously described^4^. Subsequently, the aligned sequences were analysed using Jalview and figures created with FigTree v1.4.4 (ref. ^64^).

### Proteomic analyses

The microbial cell pellets were dissolved in 100 μl of lysis buffer (2% sodium dodecyl sulfate (SDS), 5 mM EDTA, 0.2 M HEPES, pH 8.0) and shaken at 2,000 rpm at room temperature for 5 min, followed by ultrasonication for 4 min (30-s on–off pulses, 40% amplitude, Fisherbrand Model 505, Thermo Fisher Scientific). Cell lysates were quantified with bicinchoninic acid (BCA) assay (Pierce BCA Protein Assay Kit). A total of 25 μg of protein was transferred to a low-binding tube, and volume was normalized to 100 μl with lysis buffer. Proteins were reduced and alkylated by the addition of 5 mM tris(2-carboxyethyl)phosphine (TCEP) and 20 mM iodoacetamide (IAA) followed by incubation at 60 °C for 45 min (covered from light). The proteins were digested with trypsin and LysC (Pierce Trypsin/LysC Protease Mix, MS grade) on magnetic beads (1:1 mix of Sera-Mag SpeedBead carboxylate-modified [E3] and [E7] magnetic particles, Cytiva) according to single-pot, solid-phase-enhanced sample-preparation (SP3) protocol^65^. Peptide yield was measured with a microvolume spectrophotometer (Nano Drop 2000; Thermo Fisher Scientific) at 280 nm wavelength. The samples were acidified with trifluoroacetic acid (TFA) to a final concentration of 0.5%, and 15 μg of peptides was cleaned and stored on C18-StageTip filters at −20 °C before analysis^66^. Peptides were analysed by LC–MS/MS with an EASY-nLC 1200 system (Thermo Fisher Scientific) coupled to a Q-Exactive HF-X mass spectrometer (Thermo Fisher Scientific) through a nanoelectrospray ion source. An in-house column (200 mm × 0.075 mm inner diameter) packed with Reprosil-Pur C18-AQ 3-μm particles (Dr. Maisch) was used for peptide separation, using the following gradient: 5–30% B over 120 min, 30–45% B over 15 min and 45–100% B over 5 min and held for an additional 10 min on 100% B, at a flow rate of 250 nl min^−1^ (A: 0.1% FA, B: 80% ACN, 0.1% FA). The mass spectrometer was operated at 250 °C capillary temperature and 2.0 kV spray voltage. Full mass spectra were acquired over a mass range from *m*/*z* 400 to 1,600 with resolution of 60,000 (*m*/*z* 200) after accumulation of ions to a 3 × 10^6^ target value based on predictive automatic gain control (AGC) from the previous full scan. The 12 most intense peaks with a charge state ≥2 were fragmented in the higher-energy collisional dissociation (HCD) collision cell (normalized collision energy of 27%), and the tandem mass spectrum was acquired with a resolution of 15,000, with an AGC target value of 1 × 10^5^. Dynamic exclusion was set to 30 s, and the maximum allowed ion accumulation times were 20 ms for full MS scans and 50 ms for tandem mass spectra. Protein sequences for *A. muciniphila* ATCC BAA-835 were downloaded from the National Center for Biotechnlogy Information (NCBI) GenBank database (downloaded 21 April 2025) and supplemented with an in-house database containing human and mouse mucin sequences (https://www.medkem.gu.se/mucinbiology/databases/)^67^. The MS/MS spectra were analysed with MaxQuant (v2.6.5.0)^68^. Searches were performed using trypsin as an enzyme and a maximum of two missed cleavages. Precursor tolerance of 20 ppm was used in the first search for recalibration, followed by 4.5 ppm for the main search and 20 ppm for fragment ions. Carbamidomethylation of cysteine was set as a fixed modification, and methionine oxidation and protein N-terminal acetylation were set as variable modifications. The required false discovery rate (FDR) was set to 1% at both peptide and protein levels, and the minimum required peptide length was set to seven amino acids. The protein identification and quantification data were further analysed with Perseus (version 2.0.11)^69^. The same mass spectrometry method as described above, with the exception of a shortened 40-min gradient, was used to assess the presence of glycans and glycopeptides in the porcine colonic glycoprotein fraction. Mass spectrometry data were converted to peaklists using MSConvert, and spectra containing the following oxonium or immonium fragment ions: HexNAx 138.055, proline 70.065 and threonine 74.006 ± 0.001 Da were extracted with a custom python script. The mass spectrometry proteomics data have been deposited to the ProteomeXchange Consortium via the PRIDE partner repository^70^.

### Reporting summary

Further information on research design is available in the Nature Portfolio Reporting Summary linked to this article.

## Supplementary information

Supplementary Information Supplementary Figs. 1–8.

Reporting Summary

Peer Review File

Supplementary Table Supplementary Tables 1–9.

Supplementary Data 1 Proteomic analysis of water-soluble PCM extract.

Supplementary Data 2 Mass spectrometry anlysis of gMOs and gpcMOs.

Supplementary Data 3 Proteomic analysis of *A. muciniphila* grown on varied monosaccharide and mucin substrates.

Supplementary Data 4 Mass spectrometry analysis of sulfatase-treated cMO samples.

## Source data

Source Data Fig. 1 Raw and plotted growth curve data and uncropped gel.

Source Data Fig. 2 Raw and plotted enzyme kinetic and HPAEC data.

Source Data Fig. 3 Raw and plotted whole-cell assay HPAEC data.

Source Data Fig. 4 Raw and plotted growth, kinetic and HPAEC data.

Source Data Fig. 5 HPAEC data and uncropped TLC images.

Source Data Fig. 6 Uncropped gels and western blots.

Source Data Extended Data Fig. 1 Raw and plotted growth curve data.

Source Data Extended Data Fig. 2 Raw and plotted HPAEC data.

Source Data Extended Data Fig. 3 HPAEC data, TLC images and DSF data.

Source Data Extended Data Fig. 4 Raw and plotted whole-cell assay HPAEC data.

Source Data Extended Data Fig. 5 Molecular dynamics data.

Source Data Extended Data Fig. 8 HPAEC data for activity in the media.

Source Data Extended Data Fig. 10 Raw and plotted growth curve data.

## Data Availability

The crystal structure datasets generated have been deposited in the PDB under the following accession numbers: 9S7X, 9S85, 9S88, 9S8A, 9SF6, 9S8X, 9S8Y and 9S8F. Proteomic datasets have been deposited via PRIDE: PXD078370. Glycomics raw files were uploaded to Glycopost (https://glycopost.glycosmos.org/entry/GPST000622.0). Information on all other data and materials are available in the Article and Supplementary Information. Source data are provided with this paper.

## Code availability

Code generated for filtering mass specterometry data to isolate and extract only the spectra that contain a specific target ion is available via GitHub at https://github.com/SjoerdvanderPost/MS_peaklist_ion_specific_parser.
