# Publisher Correction: Unique adaptations in sulfatases underpin colonic mucin degradation by *Akkermansia muciniphila*

**DOI:** 10.1038/s41564-026-02504-2

**Published:** 2026-09-09

**Authors:** Debajit Dey, Naba D. Salman, Charles W. E. Tomlinson, Chunsheng Jin, Grete Raba, Sadie R. Schaus, Marcus Nilsson, Zak McIver, Reyme Herman, Adam Simpkin, Matthew Davy, Daniel J. Rigden, Mirjam Czjzek, Dominic P. Byrne, Mihai Oltean, Alexander Case, Christoph G. Baumann, Gareth S. A. Wright, Sjoerd van der Post, Edwin A. Yates, Eric C. Martens, Lauren Davey, Ana S. Luis, Alan Cartmell

**Affiliations:** 1 Department of Biology, University of York, York, UK; 2 York Structural Biology Laboratory (YSBL), Department of Chemistry, University of York, York, UK; 3 York Biomedical Research Institute (YBRI), University of York, York, UK; 4 Institute of Biomedicine, Department of Medical Biochemistry and Cell Biology, University of Gothenburg, Gothenburg, Sweden; 5 Proteomics Core Facility at Sahlgrenska Academy, University of Gothenburg, Gothenburg, Sweden; 6 Department of Chemistry and Biotechnology, Tallinn University of Technology, Tallinn, Estonia; 7 Department of Microbiology and Immunology, University of Michigan, Ann Arbor, MI USA; 8 Department of Biochemistry, Cell and Systems Biology, Institute of Systems, Molecular and Integrative Biology, University of Liverpool, Liverpool, UK; 9 Integrative Biology of Marine Models, UMR 8227, Station Biologique de Roscoff, CNRS, Sorbonne Université, Univ Paris 06, Roscoff, France; 10 Department of Transplantation Surgery, Institute for Clinical Sciences, Sahlgrenska Academy, University of Gothenburg, Gothenburg, Sweden; 11 Transplant Institute, Sahlgrenska University Hospital, Gothenburg, Sweden; 12 School of Life Sciences, University of Essex, Colchester, UK; 13 Department of Biochemistry and Microbiology, University of Victoria, Victoria, British Columbia Canada; 14 SciLifeLab, University of Gothenburg, Gothenburg, Sweden

**Keywords:** Glycobiology, Microbiome, X-ray crystallography, Hydrolases

Correction to: *Nature Microbiology* https://doi.org/10.1038/s41564-026-02424-1, published online 27 July 2026.

In the version of the article initially published, in Fig. 4a, the units for the “*K*_m_” values were “mm” but should have been “µm”. Additionally, Adam Simpkin was originally listed with the wrong affiliation and is now listed with the Department of Biochemistry, Cell and Systems Biology, Institute of Systems, Molecular and Integrative Biology, University of Liverpool, Liverpool, UK. These corrections have been made to the HTML and PDF versions of the article.

