# Supplementary material for: Unique adaptations in sulfatases underpin colonic mucin degradation by Akkermansia muciniphila
Source: Nat Microbiol. 2026 Jul 27;11(9):2525–42. doi: 10.1038/s41564-026-02424-1 (PMC13524802; doi:10.1038/s41564-026-02424-1)
Supplement: Supplementary file 1 [file 41564_2026_2424_MOESM1_ESM.pdf]

nature microbiology

Supplementary information https://doi.org/10.1038/s41564-026-02424-1

# Unique adaptations in sulfatases underpin colonic mucin degradation by *Akkermansia muciniphila*

In the format provided by the authors and unedited

**SUPPLEMENTARY INFORMATION**

# Unique adaptations in sulfatases underpin colonic mucin degradation by *Akkermansia muciniphila*

Debajit Dey[1,2,3$], Naba D. Salman[4$], Charles W.E. Tomlinson[1,2,3], Chunsheng Jin[4,5], Grete Raba[4,6], Sadie R. Schaus[7], Marcus Nilsson[4], Zak McIver[1,2,3], Reyme Herman[1,2,3], Adam Simpkin[6], Matthew Davy[2], Daniel J Rigden[8], Mirjam Czjzek[9], Dominic P Byrne[8], Mihai Oltean[10,11], Alexander Case[1], Christoph G. Baumann[1], Gareth SA Wright[12], Sjoerd van der Post[4], Edwin A. Yates[8], Eric C. Martens[7], Lauren Davey[13], Ana S. Luis[4,14]*, and Alan Cartmell[1,2,3]*

[1]Department of Biology, University of York, Heslington, York, YO10 5DD, UK

[2]Department of Chemistry, York Structural Biology Laboratory (YSBL), University of York, Wentworth Way, York, YO10 5DD, UK

[3]York Biomedical Research Institute (YBRI), University of York, Wentworth Way, York, YO10 5DD, UK

[4]Department of Medical Biochemistry and Cell Biology, Institute of Biomedicine, University of Gothenburg, 40530, Gothenburg, Sweden

[5]Proteomics Core Facility at Sahlgrenska Academy, University of Gothenburg, 40530, Gothenburg, Sweden

[6]Department of Chemistry and Biotechnology, Tallinn University of Technology, 12618, Tallinn, Estonia

[7]Department of Microbiology and Immunology, University of Michigan, Ann Arbor, MI 48109, USA

[8]Department of Biochemistry, Cell and Systems Biology, Institute of Systems, Molecular and Integrative Biology, University of Liverpool, Liverpool L69 7ZB, UK

[9]Sorbonne Université, Univ Paris 06, CNRS, UMR 8227, Integrative Biology of Marine Models, Station Biologique de Roscoff, CS 90074, Roscoff, Bretagne, France.

[10]Department of Transplantation Surgery, Institute for Clinical Sciences, Sahlgrenska Academy, University of Gothenburg, 41345, Gothenburg, Sweden

[11]Transplant Institute, Sahlgrenska University Hospital, 41345, Gothenburg, Sweden

[12]School of Life Sciences, University of Essex, Wivenhoe Park, Colchester, CO4 3SQ, UK

[13]Department of Biochemistry and Microbiology, University of Victoria, Victoria, British Columbia, Canada

[14]SciLifeLab, University of Gothenburg, 41390 Gothenburg, Sweden

[$]These authors contributed equally to the work

*To whom correspondence should be addressed:
alan.cartmell@york.ac.uk and ana.luis@medkem.gu.se

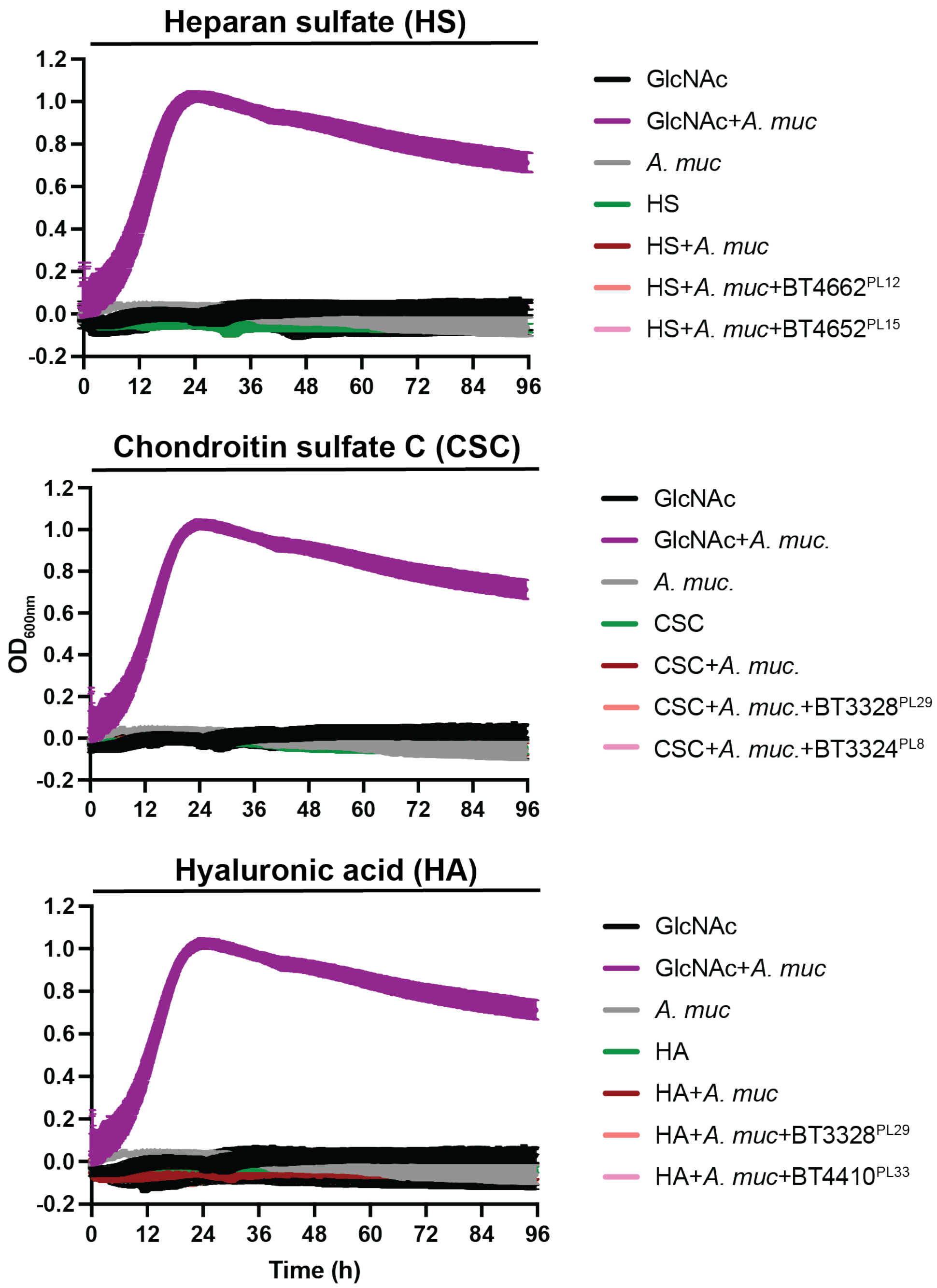


**Supplementary figure 1. *A. muciniphila* fails to grow on intact glycosaminoglycans or their breakdown products.**

*A. muciniphila* was grown on intact glycosaminoglycans, and glycosaminoglycans with *B. theta.* polysaccharide lyases supplemented into the media at a concentration of 1 μM. For heparan sulfate, BT4662$^{PL12}$ is the surface *endo*-acting enzyme from *B. theta*. that produces large fragments, whilst BT4652$^{PL15}$ is an *exo*-acting periplasmic enzyme producing exclusively disaccharide end products. For chondroitin sulfate C, BT3328$^{PL29}$ is the surface *endo*-acting enzyme from *B. theta*. that produces large fragments, whilst BT3324$^{PL8}$ is an *exo*-acting periplasmic enzyme producing exclusively disaccharide end products. For hyaluronic acid, BT3328$^{PL29}$ is the surface *endo*-acting enzyme from *B. theta*. that produces large fragments, whilst BT4410$^{PL33}$ is a processive *endo*-acting periplasmic enzyme producing disaccharide end products. All growths were technical triplicates performed in a 96 well format.

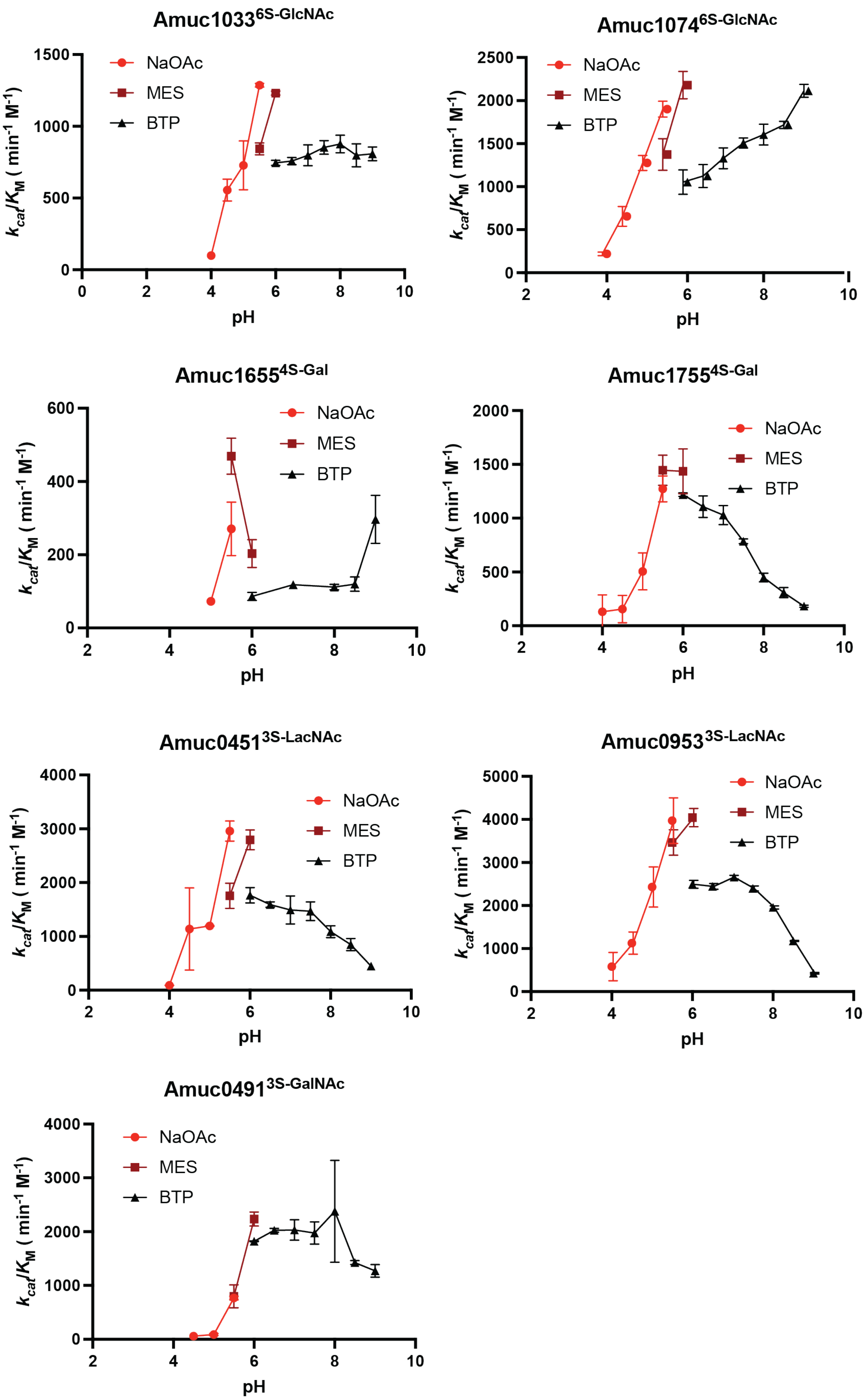


**Supplementary figure 2. The pH optima of *A. muciniphila* carbohydrate sulfatases.**

Graphs showing the pH optima for *A. muciniphila* sulfatases. All reactions were performed using 100 mM of the appropriate buffer supplemented with 150 mM NaCl and 5 mM $CaCl_2$. A substrate concentration of 1 µM was used and enzyme concentrations of between 0.1 – 2 µM were deployed depending on the enzyme.

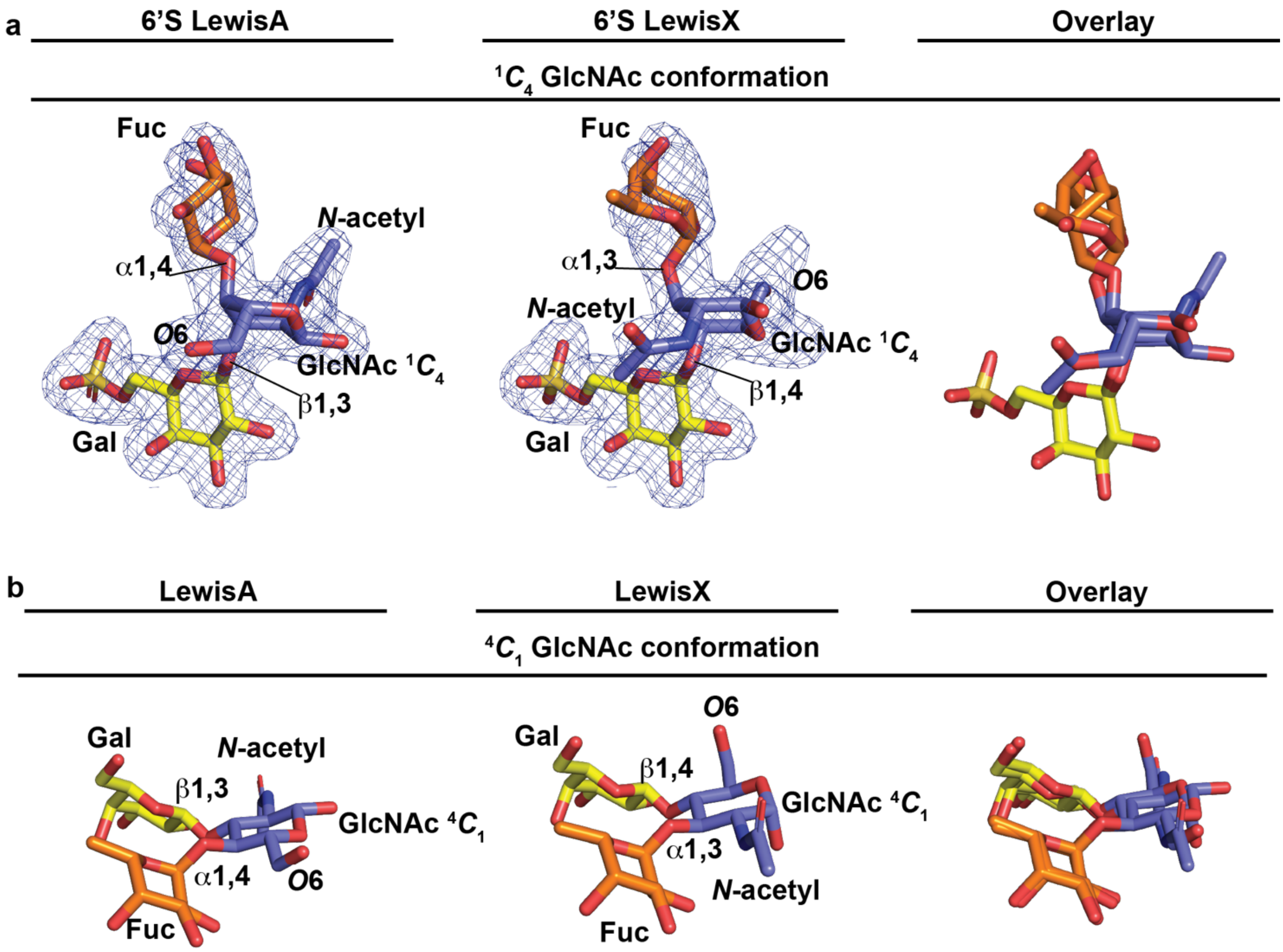


**Supplementary figure 3. Conformations of 6'S-Lewis and Lewis antigens.**

**a.** left, 6'S-LewisA from the Amuc0121$^{6S\text{-}Gal}$ modelled into the weighted 2mFobs-DFc electron density map, contoured at 1 σ, for the 6'S-LewisA substrate. It can be seen clearly that GlcNAc adopts a strained $^1C_4$ conformation. Middle, 6'S-LewisX modelled into the electron density map for 6'S-LewisA. It can be seen that the sugar broadly fits the electron density in the same conformation as observed for 6'S-LewisA with the GlcNAc flipped due to the difference in glycosidic linkages. Right, an overlay of 6'S-LewisA and 6'S-LewisX that have been modelled in the 2mFobs-DFc electron density map of the Amuc0121$^{6S\text{-}Gal}$-6'S-LewisA complex. **b.** left, LewisA substrate with the GlcNAc in a $^4C_1$ conformation from spGH29 fucosidase (PDB: 6OR4). Middle, LewisX substrate with the GlcNAc in a $^4C_1$ conformation from spGH29 fucosidase (PDB: 6ORF). Right, an overlay of LewisA and LewisX from PDBs 6OR4 and 6ORF showing that both adopt similar conformations when bound to spGH29.

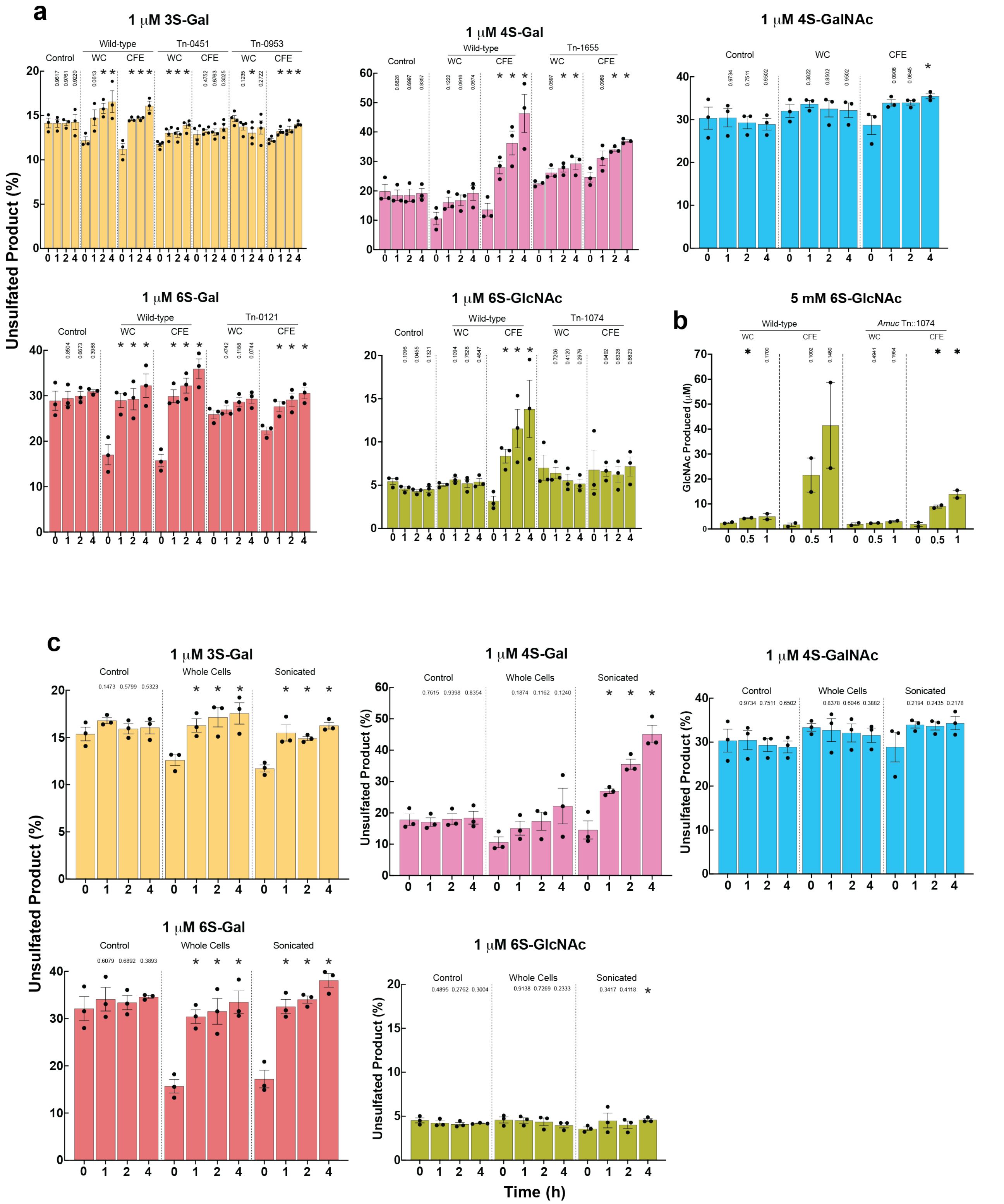


**Supplementary figure 4. Non-baseline corrected whole cell assay data.**

**a.** HPAEC based cell assays with wildtype and transposon mutant sulfatases. Cells were grown, with sPGM as their carbon source, to mid-exponential phase before being left in aerobic conditions for ~30 mins to arrest growth. These were either left as whole cells or prepared as CFE and 1 μM of a fluorescent substrates added to the media and sampled periodically. Experiments were at least biological triplicates. Control samples were cells that had been heat-deactivated at 95℃ for 10 minutes. Statistical significance of time points versus control was calculated using an unpaired Student's t-test; an asterisk indicates a value <0.05, otherwise the value is stated. **b.** HPAEC based

cell assays with wildtype and transposon mutant sulfatases. Cells were grown to mid-exponential phase before being left in aerobic conditions for ~30 mins to arrest growth. These were washed with PBS and either left as whole cells or sonicated before adding 5 mM 6S-GlcNAc. These experiments were carried out as biological duplicates. Statistical significance of time points versus control was calculated using an unpaired students t-test; an asterisk indicates a value <0.05, otherwise the value is stated.**c.** HPAEC-based cell assays with wildtype and transposon mutant sulfatases. Cells were grown with *N*-acetyl-D-glucosamine (GlcNAc) as their carbon source, to mid-exponential phase before being left in aerobic conditions for ~30 mins to arrest growth. These were either left as whole cells or sonicated, and 1 μM of a fluorescent substrates added to the media and sampled periodically. Data points are from biological triplicates with standard error of the mean shown as error bars. Statistical significance of time points versus control was calculated using an unpaired one way students t-test; an asterisk indicates a value <0.05, otherwise the value is stated.

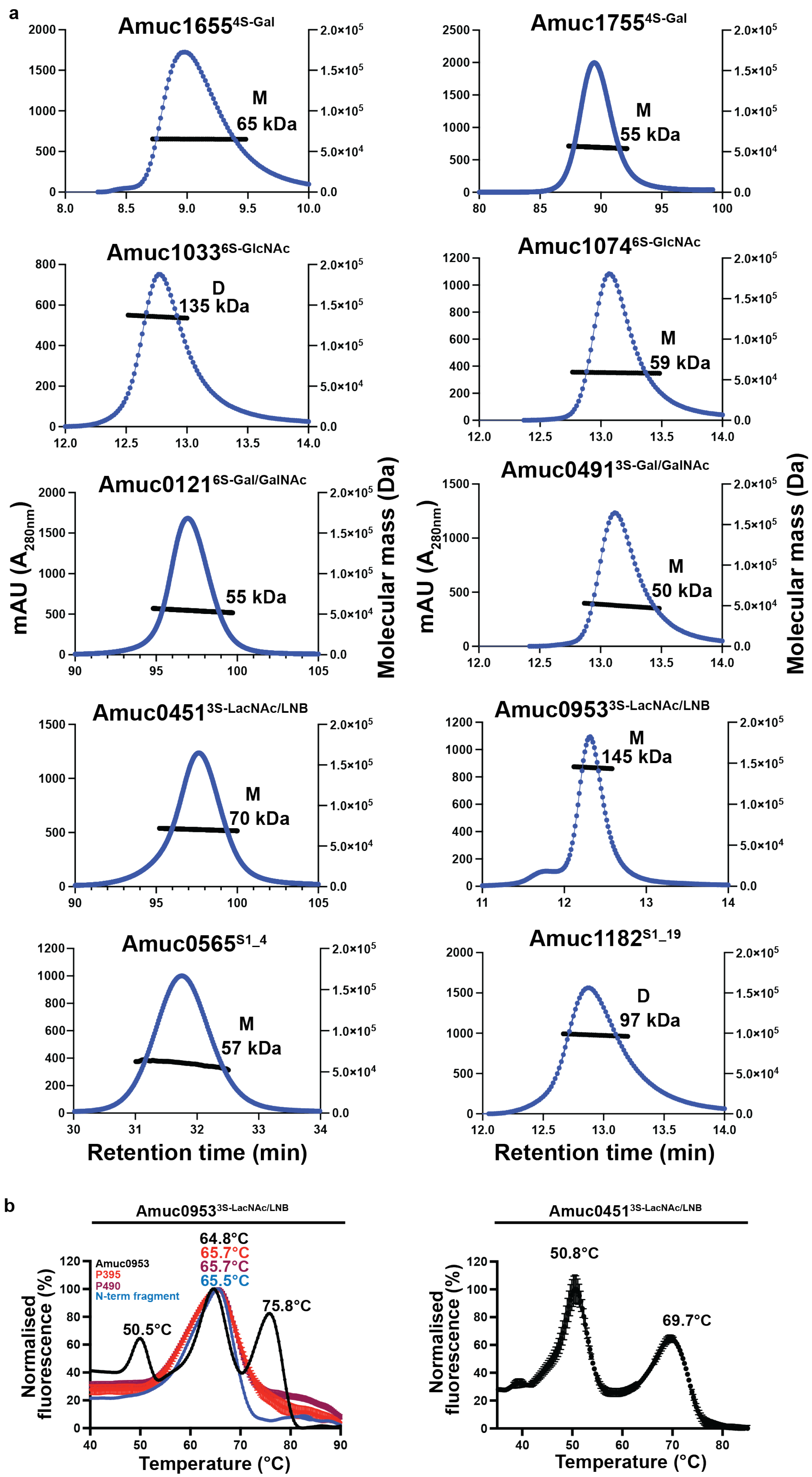

a
Amuc1655 4S-Gal
M
65 kDa
Amuc1755 4S-Gal
M
55 kDa
Amuc1033 6S-GlcNAc
D
135 kDa
Amuc1074 6S-GlcNAc
M
59 kDa
Amuc0121 6S-Gal/GalNAc
55 kDa
Amuc0491 3S-Gal/GalNAc
M
50 kDa
Amuc0451 3S-LacNAc/LNB
M
70 kDa
Amuc0953 3S-LacNAc/LNB
M
145 kDa
Amuc0565 S1_4
M
57 kDa
Amuc1182 S1_19
D
97 kDa
mAU (A280nm)
Molecular mass (Da)
Retention time (min)
b
Amuc0953 3S-LacNAc/LNB
Amuc0953
P395
P490
N-term fragment
64.8°C
65.7°C
65.7°C
65.5°C
50.5°C
75.8°C
Normalised fluorescence (%)
Temperature (°C)
Amuc0451 3S-LacNAc/LNB
50.8°C
69.7°C

**Supplementary figure 5. Size determination and biophysical properties of *A. muciniphila* S1 sulfatases**

**a.** Size-exclusion chromatography coupled to light scattering profiles for *A. muciniphila* S1 sulfatases. In each case 5 mg/ml protein was utilised. **b.** Differential scanning fluorimetry profiles for Amuc0953$^{\text{3S-LacNAc/LNB}}$ and Amuc0953$^{\text{3S-LacNAc/LNB}}$ showing multiple, distinct unfolding events.

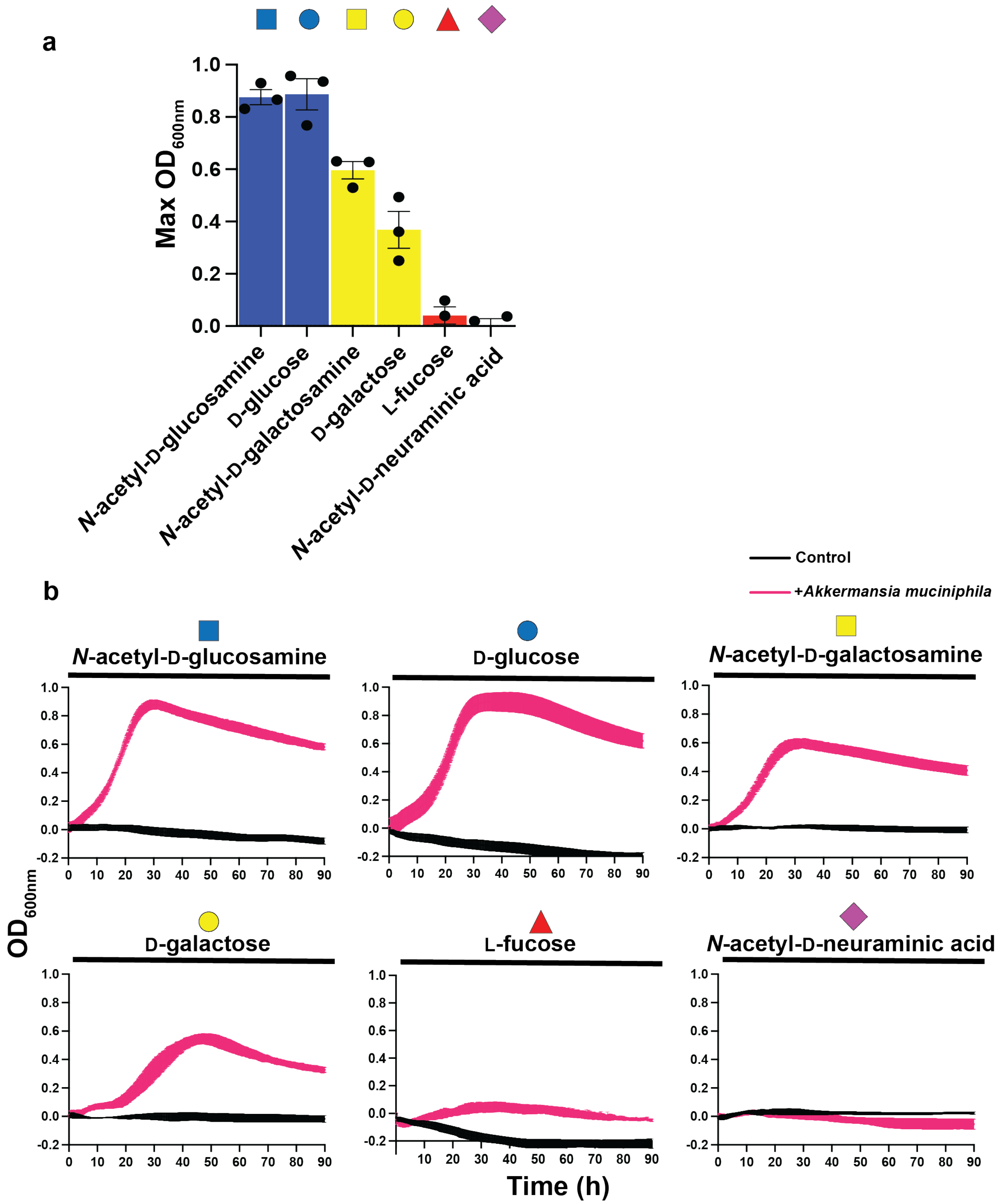


**Supplementary figure 6. Growth of *A. muciniphila* on monosaccharide growth substrates.**
**a.** Bar chart showing the maximum $OD_{600nm}$ each of the tested monosaccharides can allow *A. muciniphila* to reach **b.** Individual growth curves of *A. muciniphila* grown on monosaccharide substrates. All monosaccharides were at 10 mg/ml and the data were technical triplicates.

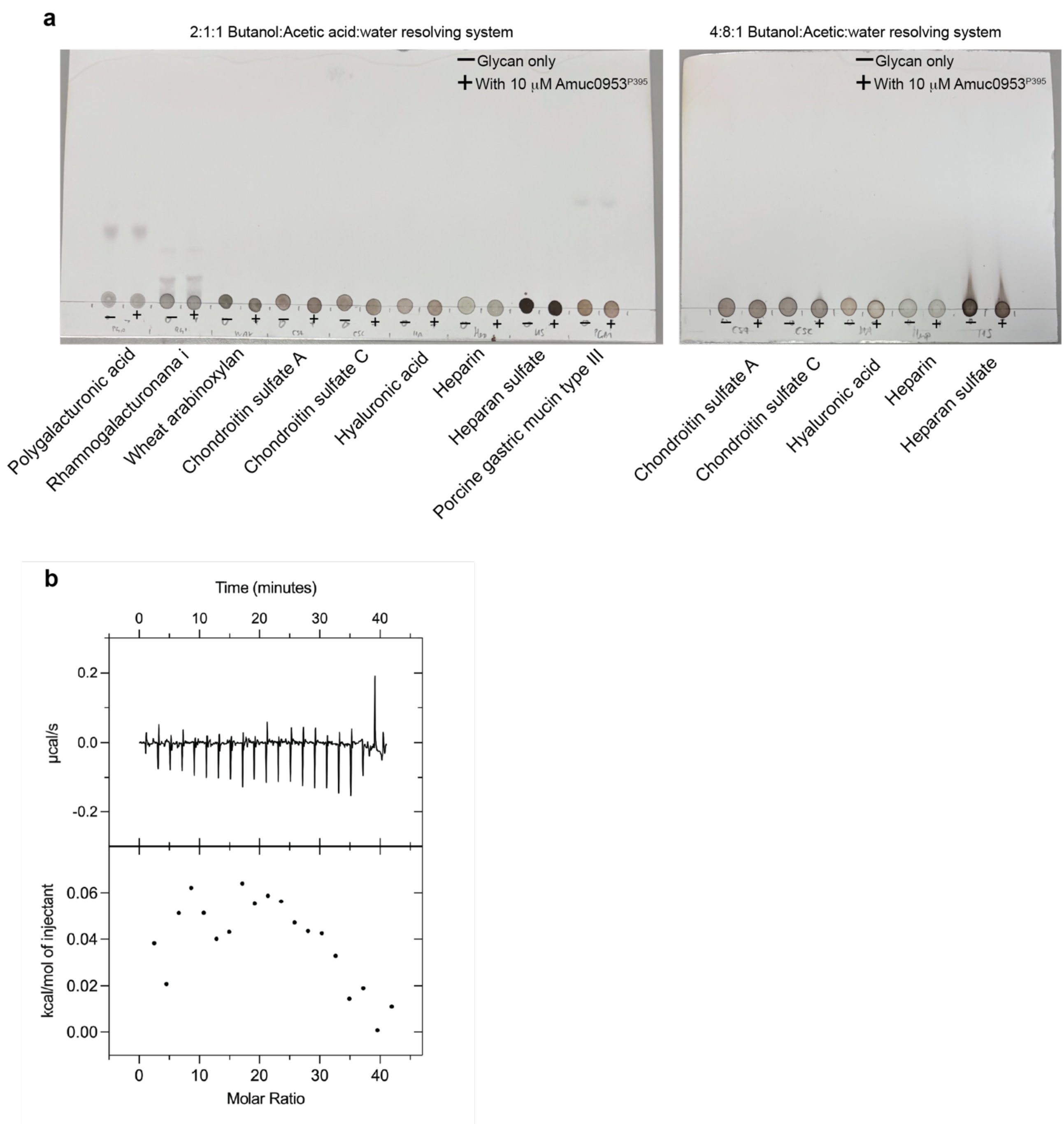


**Supplementary figure 7. Testing of Amuc0953$^{P395}$ for catalytic and binding activity against selected host glycans, and comparison to CBM89.**

**a.** Thin-layer chromatography of various polysaccharides incubated with Amuc0953$^{P395}$. For highly charged and sulfated glycans, a second solvent system was used to ensure that any oligosaccharide products produced had the ability to migrate. All reactions were in 10 mM HEPES pH 7.0, with 150 mM NaCl, and 10 μM Amuc0953$^{P395}$. **b.** Isothermal titration calorimetry of 50 μM Amuc0953$^{P395}$ against 5 mg/ml wheat arabinoxylan.

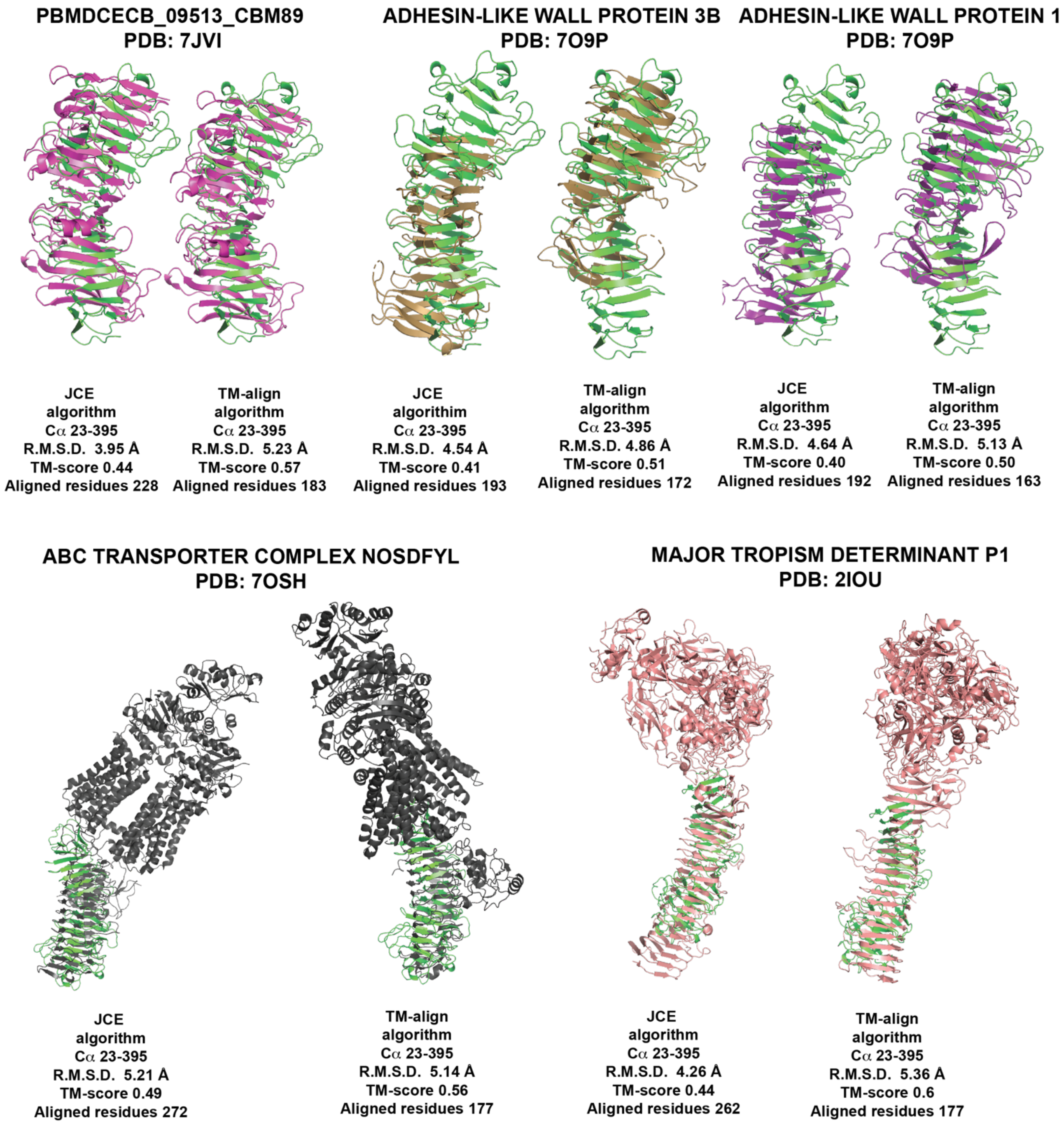


**Supplementary figure 8. Comparison of Amuc0953P395 with the closest structural matches in the PDB database as identified by PDBeFold**

Structural overlay of Amuc0953$^{P395}$, in green, with Adhesin proteins 1 and 3B from Candida glabrata (PDB: 7O9Q, 7O9P), Major tropism determinant P1 (PDB: 2IOU), PBMDCECB_09513_CBM89 (PDB: 7JVI), and ABC transporter complex nosdfyl (PDB: 7OSH) using the JCE and TM-align algorithms.
