# Supplementary material for: Unique adaptations in sulfatases underpin colonic mucin degradation by Akkermansia muciniphila
Source: Nat Microbiol. 2026 Jul 27;11(9):2525–42. doi: 10.1038/s41564-026-02424-1 (PMC13524802; doi:10.1038/s41564-026-02424-1)
Supplement: Supplementary file 2 [file 41564_2026_2424_MOESM2_ESM.pdf]

nature portfolio

Corresponding author(s): Alan Cartmell, Ana S Luis

Last updated by author(s): 30/03/26

Reporting Summary

Nature Portfolio wishes to improve the reproducibility of the work that we publish. This form provides structure for consistency and transparency in reporting. For further information on Nature Portfolio policies, see our Editorial Policies and the Editorial Policy Checklist.

Statistics

For all statistical analyses, confirm that the following items are present in the figure legend, table legend, main text, or Methods section.

n/a	Confirmed	
☐	☒	The exact sample size (*n*) for each experimental group/condition, given as a discrete number and unit of measurement
☒	☐	A statement on whether measurements were taken from distinct samples or whether the same sample was measured repeatedly
☐	☒	The statistical test(s) used AND whether they are one- or two-sided<br>*Only common tests should be described solely by name; describe more complex techniques in the Methods section.*
☒	☐	A description of all covariates tested
☒	☐	A description of any assumptions or corrections, such as tests of normality and adjustment for multiple comparisons
☐	☒	A full description of the statistical parameters including central tendency (e.g. means) or other basic estimates (e.g. regression coefficient) AND variation (e.g. standard deviation) or associated estimates of uncertainty (e.g. confidence intervals)
☐	☒	For null hypothesis testing, the test statistic (e.g. *F*, *t*, *r*) with confidence intervals, effect sizes, degrees of freedom and *P* value noted<br>*Give P values as exact values whenever suitable.*
☒	☐	For Bayesian analysis, information on the choice of priors and Markov chain Monte Carlo settings
☒	☐	For hierarchical and complex designs, identification of the appropriate level for tests and full reporting of outcomes
☒	☐	Estimates of effect sizes (e.g. Cohen's *d*, Pearson's *r*), indicating how they were calculated

Our web collection on statistics for biologists contains articles on many of the points above.

Software and code

Policy information about availability of computer code

Data collection	Chromeleon chromatography data software v7.3, Alphafold2 collab, SignalP6.0, All-atom molecular dynamics simulations were performed in GROMACS 2023 using the Charmm36m forcefield. MAFFT was used for sequence alignments. ITC data analysis was performed using MicroCal PEAQ-ITC Analysis software (Malvern). Glycan MS Data acquisition and processing were conducted with Xcalibur software (version 2.0.7). Glycans were identified from their MS/MS spectra by manual annotation and validated with available structures stored in the UniCarb-DB database (2020-01 version)
Data analysis	Protein sequences for Akkermansia muciniphila ATCC BAA-835 were downloaded from the NCBI GenBank database (downloaded 2025.04.21) and supplemented with an in-house database containing human and mouse mucin sequences (https://www.medkem.gu.se/mucinbiology/databases/). The MS/MS spectra were analysed with MaxQuant (v2.6.5.0), CPP4i2 crystallographic suite was used for X-ray data analysis and structural solution and refinement. Xia2 automated data pipelines were used for indexing and scaling X-ray data. NMR Spectra were processed using Bruker TopSpin 3.4 and 4.0 software.Structural Figures were made using Pymol (The PyMOL Molecular graphics system, Version 2.0 Schrodinger, LLC.) Graphpad Prism to produce graphs and quantify kinetic data. JalView was used to visualise aligned sequence data and Figtree v1.4.4. to make trees.

For manuscripts utilizing custom algorithms or software that are central to the research but not yet described in published literature, software must be made available to editors and reviewers. We strongly encourage code deposition in a community repository (e.g. GitHub). See the Nature Portfolio guidelines for submitting code & software for further information.

Data

Policy information about availability of data

All manuscripts must include a data availability statement. This statement should provide the following information, where applicable:

- Accession codes, unique identifiers, or web links for publicly available datasets
- A description of any restrictions on data availability
- For clinical datasets or third party data, please ensure that the statement adheres to our policy

The crystal structure datasets generated have been deposited in the in the Protein Data Bank (PDB) under the following accession numbers: 9S7X, 9S85, 9S88, 9S8A, 9SF6, 9S8X, 9S8Y, and 9S8F. Proteomic datasets have been deposited via PRIDE. Glycomics raw files were uploaded to Glycopost (https://glycopost.glycosmos.org/entry/GPST000622). Information on all other data and materials are contained within the main manuscript and Supplemental Information.

Research involving human participants, their data, or biological material

Policy information about studies with human participants or human data. See also policy information about sex, gender (identity/presentation), and sexual orientation and race, ethnicity and racism.

Reporting on sex and gender	N/A
Reporting on race, ethnicity, or other socially relevant groupings	N/A
Population characteristics	N/A
Recruitment	N/A
Ethics oversight	N/A

Note that full information on the approval of the study protocol must also be provided in the manuscript.

Field-specific reporting

Please select the one below that is the best fit for your research. If you are not sure, read the appropriate sections before making your selection.

☒ Life sciences ☐ Behavioural & social sciences ☐ Ecological, evolutionary & environmental sciences

For a reference copy of the document with all sections, see nature.com/documents/nr-reporting-summary-flat.pdf

Life sciences study design

All studies must disclose on these points even when the disclosure is negative.

Sample size	Sample size calculations were not needed. All experiments bacterial and enzymology experiments were carried in triplicate with positive and negative controls as appropriate. X-ray crystallogrpahic experiments require a single high
Data exclusions	None
Replication	All data was conducted in at least triplicate and no issues with reproducibility were observed. The exclusion to this is X-ray crystallographic experiments were a solved structure is from a dataset derived from an appropiately diffracting crystal typically the one yielding the highest resolution data.
Randomization	Randomization was not necessary for the experimental work in this study. Negative and positive controls were included in experimental setups were appropiate.
Blinding	Not necessary for the work. For examples the researcher must know the enzyme and substrate they are working on to calculate concentrations, rates, and mass calculations. Same is true for crystallography and MD simulations. For the construction of phylogenetic trees the data must be known.

Reporting for specific materials, systems and methods

We require information from authors about some types of materials, experimental systems and methods used in many studies. Here, indicate whether each material, system or method listed is relevant to your study. If you are not sure if a list item applies to your research, read the appropriate section before selecting a response.

Materials & experimental systems

n/a	Involved in the study	
☐	☒	Antibodies
☒	☐	Eukaryotic cell lines
☒	☐	Palaeontology and archaeology
☒	☐	Animals and other organisms
☒	☐	Clinical data
☒	☐	Dual use research of concern
☒	☐	Plants

Methods

n/a	Involved in the study	
☒	☐	ChIP-seq
☒	☐	Flow cytometry
☒	☐	MRI-based neuroimaging

Antibodies

Antibodies used

Membranes were then blotted with a mouse anti-his antibody (ThermoFisher MA1-21315), 1:5000 dilution, in PBS with 5 % milk powder overnight at 4ºC. Membranes were the washed 3 times with PBS and 0.1 % tween for 5 minutes each and blotted with rabbit anti mouse conjugated to HRP (ThermoFisher 31450), and developed with Clarity™ Western ECL Substrate (BioRad 1705060).

Validation

https://www.thermofisher.com/uk/en/home/life-science/antibodies/invitrogen-antibody-validation.html

Plants

Seed stocks

N/A

Novel plant genotypes

N/A

Authentication

N/A
