# Supplementary material for: Unique adaptations in sulfatases underpin colonic mucin degradation by Akkermansia muciniphila
Source: Nat Microbiol. 2026 Jul 27;11(9):2525–42. doi: 10.1038/s41564-026-02424-1 (PMC13524802; doi:10.1038/s41564-026-02424-1)
Supplement: Supplementary file 3 [file 41564_2026_2424_MOESM3_ESM.pdf]

nature portfolio

Peer Review File

Unique adaptations in sulfatases underpin colonic mucin degradation by Akkermansia muciniphila

Corresponding Author: Dr Alan cartmell

Version 0:

Reviewer comments:

Reviewer #1

(Remarks to the Author)
Salman et al. have used a combination of growth assays, whole cell assays, biochemistry, structural biology, and bioinformatics to assemble a deeper understanding of the sulfatases in Akkermansia muciniphila. There is further clarity around the preferred substrates for some of these sulfatases, but transposon mutants showed little to no phenotypes when grown on mucin in comparison to the wild type. I have several questions/clarifications for the non-reproducibility of one of the substrates, growth curves, and the whole cell assays. Some of the interpretations may be going too far e.g. linking localisation to cross-feeding/competition, tn mutant lag phases. All these are detailed below and I hope they are helpful.

My interpretation is that the novel parts are 1) the 0121 structure and the activities against 6'S-Lewis A and Lewis X (although these activities are very weak?) 2) the CBM and 3) the inhibition data. However, these get a bit lost in the current narrative and you may want to elaborate on their analysis and conversation around them to bring out the parts of the work that is new information.

Narrative
• The abstract jumps around in terms of topic/focus and the main point the authors are trying to make/focus on is not clear e.g. what are the unique adaptations, it's not clear why the localisation of de-sulfation matters as a point here, "paints a contrasting picture" but how? It's a bit cryptic. Do you not want to focus on your novel parts?
• Throughout the abstract and introduction a comparison is made between the way Akkermansia and btheta deploy sulfatases – but it is not clear what the differences are or why that really matters until the discussion e.g. line 104 the differences are not clear or why different is interesting.
• The reader is drip-fed information about the Btheta enzymes throughout and then you don't say until half way through the discussion that the knockout of just one sulfatase in Btheta kills its growth on mucin whereas for Akkermansia you don't see any impact with sulfatase mutants (but don't mention the Tnseq findings?)
• I would drop the comparison with Btheta until the discussion, especially lines 104-107
• If you are going to focus on the comparison between Btheta and Akkermansia then the conservation of the different sulfatases across different strains/species needs to be assessed to strengthen why that comparison is important i.e. are these sulfatase present in many microbes and thus apply to many humans etc

Major issues
• Line 122 onwards- I think it's good that you have talked about the difference between batch preparations of cMOs, but reproducibility is a key issue here and it sounds like it would be difficult for other people to replicate your work.
• Why did the growth experiments not include the control of growths of both Akkermansia and Btheta on colonic mucin? Why go straight to oligosaccharides?
• Line 130 – how do you know that the trypsin and proteinase K have done anything? From the NMR? Wouldn't a glycopeptidase be better for this substrate?
• Did you remove the trypsin before growth? How do you know that it's not being used as a substrate? The details about the preparation of the trypsin and proteinase K treated substrates is missing from the materials and methods
• Supplementary Figure 3
o where are the standards?
o For the no enzyme controls in some of them, the substrate peak is unclear e.g. 3'S-LeX and 3'S-LeA.
o Personally, I'm not keen on the way the chromatograms have been presented with a time-shift as I think you may miss something. I appreciate there are pros and cons of different ways to present. Also, some are shifted and some not, so maybe pick one?
o What is the peak size expected for full de-sulfation? Why was this control not included?
o Do the substrates not resolve well on the HPAEC runs? It's difficult to tell if small product peaks are due to incomplete
• Why were the 6'S-LewisA assays with 0121 not put on the HPAEC?
• Fig 2b – can you label the different substrates/products on the TLC? It is difficult to interpret.

• Fig 2b and line 173 – The control in the top TLC looks degraded already. It's difficult to see the 0121 activity.
• I think it would be good to have a figure for the mass spec gMO and cMO results so the reader can visually compare the differences. Do the cMOs have a much higher sulfation than the gMO in your work?
• For figure 2f
o How do you end up with more than what you started with?
o Some details of the linkages are absent – is this because they are unknown?
o I think these panels can take a moment to interpret as the accompanying structure can either be a substrate or a product. Maybe both could be presented and you can say which structure the bar charts represent?
o For the panels where there's no control and a product appears (left, third one down/centre, third one down/right, second one down) does the mass spec also show the substrate? And vice versa for the disappearing substrates – does the mass spec show a product?
• You've made clear that kinetics is important for a deeper understanding of the biology but not compared and contrasted the results you present in Fig1a between the different enzymes and what these mean for the reader. There's a bit in the discussion, but needs to be brought forward in the text.
• Whole cell assays –
o Why did you do the experiments with cells grown on both mucin and GlcNAc?
o why do you start off at such a high % desulfated product (in the bar charts)?
o I was not sure what the control samples were until I looked in the methods, so you may want to put that in the main text or legend.
o Why do the controls sometimes have high % values similar to a positive whole cell assay and why do they sometimes have low values like the T=0. E.g. Fig 3 and Supp fig. 4c red 6S-Gal compared 6S-GlcNAc green
o In Supp fig 4c – what does the "(GlcNAc)" mean at the top of each bar chart? Is that what they were grown on?
o Why do you think you have such a small change in the 3S-Gal compared to other substrates?
o Why are you not getting complete desulfation for any substrate?
o Line 219-220 – "The amuc1655::Tn mutant retained extracellular activity but the increased activity upon cell lysis was markedly reduced." I think this could be phrased better
o Line 228 – I'm not sure that the CFE retains activity – it looks comparable to the WC to me, unless I'm missing something?
o Line 233 – inhibit the expression of the gene or inhibit the actual enzyme?... ah ok, so I read further now…I understand why you talk about inhibition, but would this still apply if you wash the cells etc AND is the enzyme expressed comparably under GlcNAc growth conditions. Shouldn't you have grown the cells on mucin, then washed, incubated with GlcNAc, then washed, then done the assay? I think the WC data-inhibition data-growth Figs 4a-c are nice experiments, but they are explained in an order that's difficult to follow. I think I understand, but it's not clear what exactly the different growth curves are and 253-257 needs to be clearer, the last sentence seems tacked on at the end.
• The novel CBM – the structure is comparable to known CAZymes, so what does the equivalent region on the CBM to their active sites look like i.e. is it possible to tell if the binding site of the CBM is likely to be in the same location as the active site of the CAZymes? Has this evolved to be a CBM?
• Line 344 – the tn mutants also had increased lag phases on control monosaccharides, so the effect is not to do with mucin as a substrate or the missing sulfatase
• Lines 365 – what does the Tnseq Davey paper suggest about the importance of GH16s/sulfatases/other CAZymes? Is there any evidence of this cross-feeding? Why would this benefit Amuc?

Minor issues
• Line 48 – only one species of Bacteroides has been characterised?
• Line 89 – include reference
• Line 97-99 – at this point the reader will not understand what is meant by processed and only the wild type growth curves revealed this, not the mutants?
• Line 111-113 – should this reference Figure 1 too?
• Figure 1 and Supp fig 1 – it would be useful to have all growth curves on the same scale to comparison
• Lines ~115 – Does Amuc also grow on GAGs? Probably should be mentioned in this contrast with the Btheta mutant
• Line 117 – PCM is not defined
• What do you think about the biphasic growth for the Btheta mutant on gMOs and the btheta wild type on CMO
• I find following the different substrates and what treatments they have had and which growths curves match the text quite difficult in the first section. Would it be worth labelling the figures in line with the text acronyms?
• Fig 2 - There's a mistake in the legend saying that 109s target beta-linkages
• For the non-NMR expert – which parts of the profiles correspond to glycopeptide? How can you the difference between glycopeptide and glycan+peptides? The mass spec only shows the released glycans?
• 0121 structure – It would be good to comment on how the accommodation of 6'S-LewisX compared to A might be?
• Supp figure 3 – Gal is a circle, not a curved-corners square
• Line 149-151 I am not sure of the context of this last sentence. You have stated that a sulfatase is upregulated under certain conditions and then said that this suggests something about protease activity? I think you need to spell this out better for the reader
• Line 159 – You say you worked out pH, but then you don't comment on that? What was optimal and what do you think about this from the point of view of the biology?
• Line 182 – You may want to explain the impact of method-of-production on kinetics better for the reader – what is FGly?
• Line 197 – the final sentence, similar to Btheta enzymes in what way? As in the subfamilies are acting on the same types of substrates?
• In the Figure 3 model – what is the difference between "weak sulfatase activity" and "inefficient or slow sulfatase activity"
• Fig 4c – the growth curves need to be put on the same scale
• Fig 4e – what are the different colours?
• Fig 5d – label different coloured parts of the structure. Bacon, ig-like, B-helix domain etc
• Line 354-356 – say which piece of data tells you this

• More detail needed in many of the legends
• Line 370-1 - info for the intro?
• Two paragraphs starting Line 388 – Aren't these the insights that you have solid data for, so they should be focussed on more?

Reviewer #2

(Remarks to the Author)
In this work, Dey and co-workers study the role of sulfatases from Akkermansia muciniphila, a mucin degrader and a major member of the human colonic microbiota. Using wild-type and sulfatase transposon mutants, the authors demonstrate that processed glycoproteins fragments, rather than colonic mucin glycoproteins, are the preferred substrates of the bacteria. The authors recombinantly expressed 10 sulfatases and detected activities for 8 of them, in line with recent reported study (doi:10.1038/s41564-024-01911-7), but using fluorescently labelled substrates as well as model and mucin derived glycans. The authors provide crystal structures of five sulfatases, including Amuc0121, Amuc0451, Amuc0953, Amuc1074 and Amuc1755, in the absence and presence of ligands. It is worth noting that Amuc0953 contains a novel CBM family that binds colonic mucin, which seems to be restricted to the genus Akkermansia. Based on cellular localization assays, the authors proposed a processing model for the reported sulfatases. The manuscript is well written, the interpretation of the experimental data supports the proposed model and markedly advances the field.

Some brief suggestion for improvement:

Please, incorporate images showing the overall fold of each Amuc0121, Amuc0451, Amuc0953, Amuc1074 and Amuc1755 structures in the corresponding main text figures for clarity.

Congratulations to the authors on this very nice manuscript.

Reviewer #3

(Remarks to the Author)
This manuscript by Dey, Salman et al presents a broad-scale survey of Akkermansia muciniphilia sulfatases, and their role in supporting microbial utilization of gut mucin substrates. Akkermansia muciniphilia in the gut is inversely correlated with health outcomes, so understanding the factors that support colonization is important in the context of microbiome and wider systemic health.

Here, the authors have conducted growth profiling of Akkermansia on different mucin preparations, finding that glycopeptide contexts are required for microbial utilization of mucins. There is also profiling of (limited) changes to sulfatase expression during growth on mucin, followed by fairly extensive structure/function characterization of different classes of mucins expressed by Akkermansia. A new CBM family is claimed for one particular sulfatase N-terminal domain (Amuc_0953). Finally, the effect of different Akkermansia transposon mutants on sulfated mucin utilization is explored.

Whilst the topic in this paper is of broad importance, my enthusiasm is lessened somewhat by (in my opinion) slightly incomplete claims in some areas. In particular, one of the important findings seems to be that the nature of colonic mucin substrates matters for utilization by Akkermansia, with peptidic mucins preferred. However, this observation not explored in much depth beyond growth curves.

A secondary comment is that the overall presentation of results that is hard to follow. As a caveat, I have expertise in carbohydrate enzyme mechanisms but not microbiome, so I may lack some contextual knowledge in the field, but presumably the manuscript does not want to be so specialized as to be inaccessible to outsiders.

Some scientific comments

1 – It is claimed that Akkermansia requires glycoprotein forms of mucin for proper growth with porcine colonic mucins (PCMs), but not porcine gastric mucins (PGMs). As I understand it, this stems from a serendipitous observation that a second batch of colonic mucin oligosaccharides (cMOs) was enriched in protein/peptides. This led the authors to try protease digests of cMO before bacterial growth, which subsequently supported Akkermansia growth to a limited extent. This observation seems important but its context and implications are not discussed in much detail.

My inference from the rest of the text is that more sulfated cMOs (vs PGMs) require breakdown to peptidic substrates to support sulfatase function before carbohydrates can be utilized by glycosidases. However, there don't seem to be further experiments to probe this. Functional studies on recombinant enzymes use model fluorescent mono/oligosaccharide substrates (Fig 2a,b) or whole cMOs (2f), but not cMOs in glycopeptide contexts, hence this observation of mucin context ends up being sidelined.

2 – Discovery of a new CBM family with Amuc0953. Again this seems important, but the evidence presented is a single pulldown carried out with PCM, with BSA as a control. I see negative PGM interaction is proposed in the Supplmental Information using TLC (Fig S12). However, TLC may not reveal interactions - PGM would also be a useful control in the pulldown experiment to gauge CBM interaction with more/less sulfated mucins. Similarly, differences in interaction between PCM and trypsin/proteinaseK PCM should be looked at, and if possible, also interactions with cMOs.

3 – Minimal differences in growth between Tn mutants and wt Akkermansia on a variety of mucin substrates certainly seems interesting. However, this section seems to lack some context e.g. growth lag observed with amuc0490-491::tn which is

attributed to amuc0490. What is amuc0490? It is not mentioned elsewhere in the paper. Presumably not a sulfatase?

More broadly, what is the link that drives differential growth on sPGM and gpcMOs in this case? Are there issues to do with transport of different mucin sources? Are there cooperative effects for growth that would only become apparent upon mutating more sulfatases? This should at least be discussed more in the relevant results section, or in the final discussion.

On a minor note, the overall presentation, annotation and interpretation of figures seems poor in places. Some examples (I have not checked exhaustively).

1 – Fig 2c – in the text, this data is supposed to support the fact that Amuc0121 has lower affinity for 6SLewisA vs 6SGal (L174). But the data shows a thermal shift assay. Whilst this experiment can correlate with ligand affinity, it is fundamentally measuring something else, so the interpretation is not sound.

2 – Fig 4a – Inner Michaelis-Menten plot is so small as to be unreadable. What is this dotted line representing?

3 – Fig 4e – lots of different coloured loops going on here. Needs annotation.

4 – Fig 6b – again, annotation poor for quite a complex figure. Would be better if figure caption text was instead used to annotate figure directly.

5 - Supp figure 12 - typo on top line

Reviewer #4

(Remarks to the Author)
This study by Dey et al. presents a "deep dive" into the function of the family of 10 sulphatases in mucin utlisation by the human gut bacterium Akkermansia muciniphila. The authors present bacterial growth and targeted gene knock-out data, enzymology, and structural biology to provide a rather full picture of where and how these enzymes act on specific mucin substructures. The work is timely and valuable, following closely on a recent study in this journal (ref. 34, 2025) on the topic of mucin degradation by A. muciniphila, in which the same sulphatases were identified and characterized preliminarily. The present study takes the analysis significantly further. This notwithstanding, many journals now rightly have "scoop protection" policies that mitigate concerns of "novelty", and I would suggest that that same courtesy should be applied here.

The study comprises a large, diverse, and high quality set of data, the conclusions from which are clearly described and well-supported. The following comments should be addressed to improve further the study and its presentation:

Major comments:
1. Lines 316-335 and related text elsewhere: My only major concern with this manuscript is the claim that the distinct N-terminal domain of Amuc0953 constitutes a new carbohydrate binding module family. The pull-down data is certainly indicative of mucin binding, but this does not unambiguously reveal whether the interactions are mediated by protein-carbohydrate interactions, protein-protein interactions, or a combination of both. The specific residues in Amuc0953 responsible for binding and the specific epitope(s) on mucin are unresolved. Crystallographic complexes and interaction studies with mucin fragments of defined glycan structure would go along way to resolving this question. Regardless, however, the observation that this domain is found essentially exclusively in Akkermansia indicates that creation of a new CAZy family would constitute a small family with little sequence diversity and limited predictive power across microbial taxa, which I believe is the raison d'etre of the CAZy classification and bioinformatic analysis through modular dissection.

The revelation of this new structure is clearly one of the key novel and exciting aspects of the manuscript. I would only suggest that the authors refrain from calling this a new CBM, and refer it is simply as a "novel mucin binding domain (or module)", e.g. in the title of Fig. 6, in line 316, etc.

It is clear that this new domain is not similar to CBM89, based on the analysis provided in Fig. S12 (n.b. check spelling of legend). However, comparison with this particular beta-helix domain family may not be the most appropriate. What are the top hits from a structure-based search of the entire Protein Data Bank? The authors should confirm that their analysis was not unduly biased by only considering that this might be an evolutionary derivative of a CBM lineage.

2. Line 85: Bakshani et al. (ref. 34) identified 12 potential sulphatases in A. muciniphila, but here it is stated that there are 10. Why the discrepancy? This should be addressed explicitly in the text

Minor comments:
1. Line 40 and lines 70-84: Given the mixed biological effects of A. muciniphila on hosts, should the first sentence of the abstract be more balanced, e.g. "...and has been implicated in health."? Should the key study by co-author Martens (Cell. 2016 Nov 17;167(5):1339-1353.e21. doi: 10.1016/j.cell.2016.10.043.) be cited in the context of lines 80-84?

2. Please use either "A. muciniphila" or "A. muc." consistently throughout. Like "A.", "muc." is also an abbreviation, so requires a period. Ditto for "theta."

3. Lines 145 and 149: Replace "significantly" with "highly". I believe the authors are referring to the x-axis in Fig. 1g, not the y-

axis. Throughout, the word "significant" should be reserved for use in the statistical sense.

4. Lines 214-216: This reads like a sentence fragment. Please revise.

5. Lines 237-239 and 370-378: Is the sulphation of 6S-GlcNAc in the periplasm biologically meaningful, or just a quirk of evolution? That is, is there a reason why the corresponding sulphatase is not delivered to the cell exterior? Do orthologues in other species or strains contain signal peptides for extracellular targeting?

6. Line 267: "loss of productive energy" Please rephrase in a form more typical for physical chemistry.

7. Lines 293-294: The use of the descriptor "BACON" should be deprecated. I understand that there is no experimental evidence to support the statement that these domains are carbohydrate binding. If otherwise, please provide references. The acronym can be suitably replaced with a corresponding InterPro or Pfam identifier with no detriment to the text. The authors may also wish to include a description of the overall fold of this domain, as done for other domains described in the surrounding text.

8. Line 301: Revise to "translocation of"

9. Line 305: Spell out "Histidine". "His" is easily mistaken for the pronoun at the start of the sentence.

10. Line 363: "the Bacteroides organism" is redundant in this sentence.

11. Line 394: Perhaps it is worth stating explicitly that salivary mucins would in fact be expected to transit the GI tract as a normal consequence of eating. As such, all members of the HGM/HCM might be anticipated to encounter this substrate.

12. Line 397-399, perhaps elsewhere: Is it possible that this protein requires 6S-GlcNAc to be presented on a polypeptide for maximum activity? I maybe missing something here, but in general (e.g. Fig. 3) substrate specificity seems to be considered only for free mono/oligosaccharides.

13. Line 401: "Gram" should be capitalised as it is a surname.

14. In the legends for Fig. 5d and Fig. 6a, please clarify which structure(s) is experimental and which is modeled (and if so, how).

End of comments.

Decision Letter:

10th November 2025

*Please ensure you delete the link to your author homepage in this e-mail if you wish to forward it to your co-authors.

Dear Dr Cartmell,

Thank you for your patience while your manuscript "The role of Akkermansia muciniphila sulfatases in colonic mucin utilisation" was under peer-review at Nature Microbiology. It has now been seen by 4 referees, whose expertise and comments you will find at the end of this email. Although they find your work of some potential interest, they have raised a number of concerns that will need to be addressed before we can consider publication of the work in Nature Microbiology.

In particular, multiple referees have highlighted that conclusions are overinterpreted from pull-down experiments. Therefore, we recommend that you perform additional biochemical experiments to support the interaction of the new mucin binding domain with glycans. We also recommend that you carry out more exploration of the sulphatases-glycosidases interplay, as suggested by the referees. In addition, please restructure and rephrase the manuscript to make it more accessible to the general audience.

Please note that for us to consider a revised manuscript, all the concerns, and not just the ones highlighted, must be addressed.

We are committed to providing a fair and constructive peer-review process. Please do not hesitate to contact us if there are specific requests from the reviewers that you believe are technically impossible or unlikely to yield a meaningful outcome.

We strongly support public availability of data. Please place the data used in your paper into a public data repository, if one exists, or alternatively, present the data as Source Data or Supplementary Information. If data can only be shared on request, please explain why in your Data Availability Statement, and also in the correspondence with your editor. For some data types, deposition in a public repository is mandatory - more information on our data deposition policies and available repositories can be found at https://www.nature.com/nature-research/editorial-policies/reporting-standards#availability-of-data.

Please include a data availability statement as a separate section after Methods but before references, under the heading "Data Availability". This section should inform readers about the availability of the data used to support the conclusions of your study. This information includes accession codes to public repositories (data banks for protein, DNA or RNA sequences, microarray, proteomics data etc…), references to source data published alongside the paper, unique identifiers such as URLs to data repository entries, or data set DOIs, and any other statement about data availability. At a minimum, you should include the following statement: "The data that support the findings of this study are available from the corresponding author upon request", mentioning any restrictions on availability. If DOIs are provided, we also strongly encourage including these in the Reference list (authors, title, publisher (repository name), identifier, year). For more guidance on how to write this section please see:

http://www.nature.com/authors/policies/data/data-availability-statements-data-citations.pdf

If revising your manuscript:

* Include a “Response to referees” document detailing, point-by-point, how you addressed each referee comment. If no action was taken to address a point, you must provide a compelling argument. This response will be sent back to the referees along with the revised manuscript.

* If you have not done so already we suggest that you begin to revise your manuscript so that it conforms to our Article format instructions at http://www.nature.com/nmicrobiol/info/final-submission. Refer also to any guidelines provided in this letter.

* Include a revised version of any required reporting checklist. It will be available to referees (and, potentially, statisticians) to aid in their evaluation if the manuscript goes back for peer review. A revised checklist is essential for re-review of the paper.

When submitting the revised version of your manuscript, please pay close attention to our href="https://www.nature.com/nature-portfolio/editorial-policies/image-integrity">Digital Image Integrity Guidelines. and to the following points below:

-- that unprocessed scans are clearly labelled and match the gels and western blots presented in figures.
-- that control panels for gels and western blots are appropriately described as loading on sample processing controls
-- all images in the paper are checked for duplication of panels and for splicing of gel lanes.

EXTENDED DATA FIGURES

When re-submitting your manuscript, please ensure that any supplementary figures and tables that are crucial to the manuscript’s conclusions are converted into Extended Data figures and tables to increase visibility of these data. Extended Data figures and tables are online-only (present in the online PDF and full-text HTML versions of the paper), peer-reviewed display items that provide essential background to the article but are not included in the main article due to space constraints. A maximum of ten Extended Data display items (figures and tables) is permitted.

Finally, please ensure that you retain unprocessed data and metadata files after publication, ideally archiving data in perpetuity, as these may be requested during the peer review and production process or after publication if any issues arise.

Please use the link below to submit a revised paper:

Link Redacted

Note: This url links to your confidential homepage and associated information about manuscripts you may have submitted or be reviewing for us. If you wish to forward this e-mail to co-authors, please delete this link to your homepage first.

Nature Microbiology is committed to improving transparency in authorship. As part of our efforts in this direction, we are now requesting that all authors identified as ‘corresponding author’ on published papers create and link their Open Researcher and Contributor Identifier (ORCID) with their account on the Manuscript Tracking System (MTS), prior to acceptance. This applies to primary research papers only. ORCID helps the scientific community achieve unambiguous attribution of all scholarly contributions. You can create and link your ORCID from the home page of the MTS by clicking on ‘Modify my Springer Nature account’. For more information please visit please visit www.springernature.com/orcid.

If you wish to submit a suitably revised manuscript we would hope to receive it within 6 months. If you cannot send it within this time, please let us know. We will be happy to consider your revision, even if a similar study has been accepted for publication at Nature Microbiology or published elsewhere (up to a maximum of 6 months).

In the meantime we hope that you find our referees' comments helpful.

Yours sincerely,

****
Reviewer Expertise:

Referee #1: Microbial mucin degradation
Referee #2: glycobiology, enzymology
Referee #3: glycobiology, structure biology
Referee #4: Biochemistry

Reviewer Comments:

Reviewer #1 (Remarks to the Author):

Salman et al. have used a combination of growth assays, whole cell assays, biochemistry, structural biology, and bioinformatics to assemble a deeper understanding of the sulfatases in Akkermansia muciniphila. There is further clarity around the preferred substrates for some of these sulfatases, but transposon mutants showed little to no phenotypes when grown on mucin in comparison to the wild type. I have several questions/clarifications for the non-reproducibility of one of the substrates, growth curves, and the whole cell assays. Some of the interpretations may be going too far e.g. linking localisation to cross-feeding/competition, tn mutant lag phases. All these are detailed below and I hope they are helpful.

My interpretation is that the novel parts are 1) the 0121 structure and the activities against 6'S-Lewis A and Lewis X (although these activities are very weak?) 2) the CBM and 3) the inhibition data. However, these get a bit lost in the current narrative and you may want to elaborate on their analysis and conversation around them to bring out the parts of the work that is new information.

Narrative
• The abstract jumps around in terms of topic/focus and the main point the authors are trying to make/focus on is not clear e.g. what are the unique adaptations, it's not clear why the localisation of de-sulfation matters as a point here, "paints a contrasting picture" but how? It's a bit cryptic. Do you not want to focus on your novel parts?
• Throughout the abstract and introduction a comparison is made between the way Akkermansia and btheta deploy sulfatases – but it is not clear what the differences are or why that really matters until the discussion e.g. line 104 the differences are not clear or why different is interesting.
• The reader is drip-fed information about the Btheta enzymes throughout and then you don't say until half way through the discussion that the knockout of just one sulfatase in Btheta kills its growth on mucin whereas for Akkermansia you don't see any impact with sulfatase mutants (but don't mention the Tnseq findings?)
• I would drop the comparison with Btheta until the discussion, especially lines 104-107
• If you are going to focus on the comparison between Btheta and Akkermansia then the conservation of the different sulfatases across different strains/species needs to be assessed to strengthen why that comparison is important i.e. are these sulfatase present in many microbes and thus apply to many humans etc

Major issues
• Line 122 onwards- I think it's good that you have talked about the difference between batch preparations of cMOs, but reproducibility is a key issue here and it sounds like it would be difficult for other people to replicate your work.
• Why did the growth experiments not include the control of growths of both Akkermansia and Btheta on colonic mucin? Why go straight to oligosaccharides?
• Line 130 – how do you know that the trypsin and proteinase K have done anything? From the NMR? Wouldn't a glycopeptidase be better for this substrate?
• Did you remove the trypsin before growth? How do you know that it's not being used as a substrate? The details about the preparation of the trypsin and proteinase K treated substrates is missing from the materials and methods
• Supplementary Figure 3
o where are the standards?
o For the no enzyme controls in some of them, the substrate peak is unclear e.g. 3'S-LeX and 3'S-LeA.
o Personally, I'm not keen on the way the chromatograms have been presented with a time-shift as I think you may miss something. I appreciate there are pros and cons of different ways to present. Also, some are shifted and some not, so maybe pick one?
o What is the peak size expected for full de-sulfation? Why was this control not included?
o Do the substrates not resolve well on the HPAEC runs? It's difficult to tell if small product peaks are due to incomplete
• Why were the 6'S-LewisA assays with 0121 not put on the HPAEC?
• Fig 2b – can you label the different substrates/products on the TLC? It is difficult to interpret.
• Fig 2b and line 173 – The control in the top TLC looks degraded already. It's difficult to see the 0121 activity.
• I think it would be good to have a figure for the mass spec gMO and cMO results so the reader can visually compare the differences. Do the cMOs have a much higher sulfation than the gMO in your work?
• For figure 2f
o How do you end up with more than what you started with?
o Some details of the linkages are absent – is this because they are unknown?
o I think these panels can take a moment to interpret as the accompanying structure can either be a substrate or a product. Maybe both could be presented and you can say which structure the bar charts represent?
o For the panels where there's no control and a product appears (left, third one down/centre, third one down/right, second one down) does the mass spec also show the substrate? And vice versa for the disappearing substrates – does the mass spec show a product?
• You've made clear that kinetics is important for a deeper understanding of the biology but not compared and contrasted the results you present in Fig1a between the different enzymes and what these mean for the reader. There's a bit in the discussion, but needs to be brought forward in the text.
• Whole cell assays –
o Why did you do the experiments with cells grown on both mucin and GlcNAc?
o why do you start off at such a high % desulfated product (in the bar charts)?
o I was not sure what the control samples were until I looked in the methods, so you may want to put that in the main text or legend.
o Why do the controls sometimes have high % values similar to a positive whole cell assay and why do they sometimes have low values like the T=0. E.g. Fig 3 and Supp fig. 4c red 6S-Gal compared 6S-GlcNAc green
o In Supp fig 4c – what does the "(GlcNAc)" mean at the top of each bar chart? Is that what they were grown on?
o Why do you think you have such a small change in the 3S-Gal compared to other substrates?
o Why are you not getting complete desulfation for any substrate?
o Line 219-220 – "The amuc1655::Tn mutant retained extracellular activity but the increased activity upon cell lysis was

markedly reduced." I think this could be phrased better
o Line 228 – I'm not sure that the CFE retains activity – it looks comparable to the WC to me, unless I'm missing something?
o Line 233 – inhibit the expression of the gene or inhibit the actual enzyme?... ah ok, so I read further now…I understand why you talk about inhibition, but would this still apply if you wash the cells etc AND is the enzyme expressed comparably under GlcNAc growth conditions. Shouldn't you have grown the cells on mucin, then washed, incubated with GlcNAc, then washed, then done the assay? I think the WC data-inhibition data-growth Figs 4a-c are nice experiments, but they are explained in an order that's difficult to follow. I think I understand, but it's not clear what exactly the different growth curves are and 253-257 needs to be clearer, the last sentence seems tacked on at the end.
• The novel CBM – the structure is comparable to known CAZymes, so what does the equivalent region on the CBM to their active sites look like i.e. is it possible to tell if the binding site of the CBM is likely to be in the same location as the active site of the CAZymes? Has this evolved to be a CBM?
• Line 344 – the tn mutants also had increased lag phases on control monosaccharides, so the effect is not to do with mucin as a substrate or the missing sulfatase
• Lines 365 – what does the Tnseq Davey paper suggest about the importance of GH16s/sulfatases/other CAZymes? Is there any evidence of this cross-feeding? Why would this benefit Amuc?

Minor issues
• Line 48 – only one species of Bacteroides has been characterised?
• Line 89 – include reference
• Line 97-99 – at this point the reader will not understand what is meant by processed and only the wild type growth curves revealed this, not the mutants?
• Line 111-113 – should this reference Figure 1 too?
• Figure 1 and Supp fig 1 – it would be useful to have all growth curves on the same scale to comparison
• Lines ~115 – Does Amuc also grow on GAGs? Probably should be mentioned in this contrast with the Btheta mutant
• Line 117 – PCM is not defined
• What do you think about the biphasic growth for the Btheta mutant on gMOs and the btheta wild type on CMO
• I find following the different substrates and what treatments they have had and which growths curves match the text quite difficult in the first section. Would it be worth labelling the figures in line with the text acronyms?
• Fig 2 - There's a mistake in the legend saying that 109s target beta-linkages
• For the non-NMR expert – which parts of the profiles correspond to glycopeptide? How can you the difference between glycopeptide and glycan+peptides? The mass spec only shows the released glycans?
• 0121 structure – It would be good to comment on how the accommodation of 6'S-LewisX compared to A might be?
• Supp figure 3 – Gal is a circle, not a curved-corners square
• Line 149-151 I am not sure of the context of this last sentence. You have stated that a sulfatase is upregulated under certain conditions and then said that this suggests something about protease activity? I think you need to spell this out better for the reader
• Line 159 – You say you worked out pH, but then you don't comment on that? What was optimal and what do you think about this from the point of view of the biology?
• Line 182 – You may want to explain the impact of method-of-production on kinetics better for the reader – what is FGly?
• Line 197 – the final sentence, similar to Btheta enzymes in what way? As in the subfamilies are acting on the same types of substrates?
• In the Figure 3 model – what is the difference between "weak sulfatase activity" and "inefficient or slow sulfatase activity"
• Fig 4c – the growth curves need to be put on the same scale
• Fig 4e – what are the different colours?
• Fig 5d – label different coloured parts of the structure. Bacon, ig-like, B-helix domain etc
• Line 354-356 – say which piece of data tells you this
• More detail needed in many of the legends
• Line 370-1 - info for the intro?
• Two paragraphs starting Line 388 – Aren't these the insights that you have solid data for, so they should be focussed on more?

Reviewer #2 (Remarks to the Author):

In this work, Dey and co-workers study the role of sulfatases from Akkermansia muciniphila, a mucin degrader and a major member of the human colonic microbiota. Using wild-type and sulfatase transposon mutants, the authors demonstrate that processed glycoproteins fragments, rather than colonic mucin glycoproteins, are the preferred substrates of the bacteria. The authors recombinantly expressed 10 sulfatases and detected activities for 8 of them, in line with recent reported study (doi:10.1038/s41564-024-01911-7), but using fluorescently labelled substrates as well as model and mucin derived glycans. The authors provide crystal structures of five sulfatases, including Amuc0121, Amuc0451, Amuc0953, Amuc1074 and Amuc1755, in the absence and presence of ligands. It is worth noting that Amuc0953 contains a novel CBM family that binds colonic mucin, which seems to be restricted to the genus Akkermansia. Based on cellular localization assays, the authors proposed a processing model for the reported sulfatases. The manuscript is well written, the interpretation of the experimental data supports the proposed model and markedly advances the field.

Some brief suggestion for improvement:

Please, incorporate images showing the overall fold of each Amuc0121, Amuc0451, Amuc0953, Amuc1074 and Amuc1755 structures in the corresponding main text figures for clarity.

Congratulations to the authors on this very nice manuscript.

Reviewer #3 (Remarks to the Author):

This manuscript by Dey, Salman et al presents a broad-scale survey of Akkermansia muciniphilia sulfatases, and their role in supporting microbial utilization of gut mucin substrates. Akkermansia muciniphilia in the gut is inversely correlated with health outcomes, so understanding the factors that support colonization is important in the context of microbiome and wider systemic health.

Here, the authors have conducted growth profiling of Akkermansia on different mucin preparations, finding that glycopeptide contexts are required for microbial utilization of mucins. There is also profiling of (limited) changes to sulfatase expression during growth on mucin, followed by fairly extensive structure/function characterization of different classes of mucins expressed by Akkermansia. A new CBM family is claimed for one particular sulfatase N-terminal domain (Amuc_0953). Finally, the effect of different Akkermansia transposon mutants on sulfated mucin utilization is explored.

Whilst the topic in this paper is of broad importance, my enthusiasm is lessened somewhat by (in my opinion) slightly incomplete claims in some areas. In particular, one of the important findings seems to be that the nature of colonic mucin substrates matters for utilization by Akkermansia, with peptidic mucins preferred. However, this observation not explored in much depth beyond growth curves.

A secondary comment is that the overall presentation of results that is hard to follow. As a caveat, I have expertise in carbohydrate enzyme mechanisms but not microbiome, so I may lack some contextual knowledge in the field, but presumably the manuscript does not want to be so specialized as to be inaccessible to outsiders.

Some scientific comments

1 – It is claimed that Akkermansia requires glycoprotein forms of mucin for proper growth with porcine colonic mucins (PCMs), but not porcine gastric mucins (PGMs). As I understand it, this stems from a serendipitous observation that a second batch of colonic mucin oligosaccharides (cMOs) was enriched in protein/peptides. This led the authors to try protease digests of cMO before bacterial growth, which subsequently supported Akkermansia growth to a limited extent. This observation seems important but its context and implications are not discussed in much detail.

My inference from the rest of the text is that more sulfated cMOs (vs PGMs) require breakdown to peptidic substrates to support sulfatase function before carbohydrates can be utilized by glycosidases. However, there don't seem to be further experiments to probe this. Functional studies on recombinant enzymes use model fluorescent mono/oligosaccharide substrates (Fig 2a,b) or whole cMOs (2f), but not cMOs in glycopeptide contexts, hence this observation of mucin context ends up being sidelined.

2 – Discovery of a new CBM family with Amuc0953. Again this seems important, but the evidence presented is a single pulldown carried out with PCM, with BSA as a control. I see negative PGM interaction is proposed in the Supplmental Information using TLC (Fig S12). However, TLC may not reveal interactions - PGM would also be a useful control in the pulldown experiment to gauge CBM interaction with more/less sulfated mucins. Similarly, differences in interaction between PCM and trypsin/proteinaseK PCM should be looked at, and if possible, also interactions with cMOs.

3 – Minimal differences in growth between Tn mutants and wt Akkermansia on a variety of mucin substrates certainly seems interesting. However, this section seems to lack some context e.g. growth lag observed with amuc0490-491::tn which is attributed to amuc0490. What is amuc0490? It is not mentioned elsewhere in the paper. Presumably not a sulfatase?

More broadly, what is the link that drives differential growth on sPGM and gpcMOs in this case? Are there issues to do with transport of different mucin sources? Are there cooperative effects for growth that would only become apparent upon mutating more sulfatases? This should at least be discussed more in the relevant results section, or in the final discussion.

On a minor note, the overall presentation, annotation and interpretation of figures seems poor in places. Some examples (I have not checked exhaustively).

1 – Fig 2c – in the text, this data is supposed to support the fact that Amuc0121 has lower affinity for 6SLewisA vs 6SGal (L174). But the data shows a thermal shift assay. Whilst this experiment can correlate with ligand affinity, it is fundamentally measuring something else, so the interpretation is not sound.

2 – Fig 4a – Inner Michaelis-Menten plot is so small as to be unreadable. What is this dotted line representing?

3 – Fig 4e – lots of different coloured loops going on here. Needs annotation.

4 – Fig 6b – again, annotation poor for quite a complex figure. Would be better if figure caption text was instead used to annotate figure directly.

5 - Supp figure 12 - typo on top line

Reviewer #4 (Remarks to the Author):

This study by Dey et al. presents a "deep dive" into the function of the family of 10 sulphatases in mucin utlisation by the human gut bacterium Akkermansia muciniphila. The authors present bacterial growth and targeted gene knock-out data, enzymology, and structural biology to provide a rather full picture of where and how these enzymes act on specific mucin substructures. The work is timely and valuable, following closely on a recent study in this journal (ref. 34, 2025) on the topic of mucin degradation by A. muciniphila, in which the same sulphatases were identified and characterized preliminarily. The present study takes the analysis significantly further. This notwithstanding, many journals now rightly have "scoop protection" policies that mitigate concerns of "novelty", and I would suggest that that same courtesy should be applied here.

The study comprises a large, diverse, and high quality set of data, the conclusions from which are clearly described and well-supported. The following comments should be addressed to improve further the study and its presentation:

Major comments:
1. Lines 316-335 and related text elsewhere: My only major concern with this manuscript is the claim that the distinct N-terminal domain of Amuc0953 constitutes a new carbohydrate binding module family. The pull-down data is certainly indicative of mucin binding, but this does not unambiguously reveal whether the interactions are mediated by protein-carbohydrate interactions, protein-protein interactions, or a combination of both. The specific residues in Amuc0953 responsible for binding and the specific epitope(s) on mucin are unresolved. Crystallographic complexes and interaction studies with mucin fragments of defined glycan structure would go along way to resolving this question. Regardless, however, the observation that this domain is found essentially exclusively in Akkermansia indicates that creation of a new CAZy family would constitute a small family with little sequence diversity and limited predictive power across microbial taxa, which I believe is the raison d'etre of the CAZy classification and bioinformatic analysis through modular dissection.

The revelation of this new structure is clearly one of the key novel and exciting aspects of the manuscript. I would only suggest that the authors refrain from calling this a new CBM, and refer it is simply as a "novel mucin binding domain (or module)", e.g. in the title of Fig. 6, in line 316, etc.

It is clear that this new domain is not similar to CBM89, based on the analysis provided in Fig. S12 (n.b. check spelling of legend). However, comparison with this particular beta-helix domain family may not be the most appropriate. What are the top hits from a structure-based search of the entire Protein Data Bank? The authors should confirm that their analysis was not unduly biased by only considering that this might be an evolutionary derivative of a CBM lineage.

2. Line 85: Bakshani et al. (ref. 34) identified 12 potential sulphatases in A. muciniphila, but here it is stated that there are 10. Why the discrepancy? This should be addressed explicitly in the text

Minor comments:
1. Line 40 and lines 70-84: Given the mixed biological effects of A. muciniphila on hosts, should the first sentence of the abstract be more balanced, e.g. "...and has been implicated in health."? Should the key study by co-author Martens (Cell. 2016 Nov 17;167(5):1339-1353.e21. doi: 10.1016/j.cell.2016.10.043.) be cited in the context of lines 80-84?

2. Please use either "A. muciniphila" or "A. muc." consistently throughout. Like "A.", "muc." is also an abbreviation, so requires a period. Ditto for "theta."

3. Lines 145 and 149: Replace "significantly" with "highly". I believe the authors are referring to the x-axis in Fig. 1g, not the y-axis. Throughout, the word "significant" should be reserved for use in the statistical sense.

4. Lines 214-216: This reads like a sentence fragment. Please revise.

5. Lines 237-239 and 370-378: Is the sulphation of 6S-GlcNAc in the periplasm biologically meaningful, or just a quirk of evolution? That is, is there a reason why the corresponding sulphatase is not delivered to the cell exterior? Do orthologues in other species or strains contain signal peptides for extracellular targeting?

6. Line 267: "loss of productive energy" Please rephrase in a form more typical for physical chemistry.

7. Lines 293-294: The use of the descriptor "BACON" should be deprecated. I understand that there is no experimental evidence to support the statement that these domains are carbohydrate binding. If otherwise, please provide references. The acronym can be suitably replaced with a corresponding InterPro or Pfam identifier with no detriment to the text. The authors may also wish to include a description of the overall fold of this domain, as done for other domains described in the surrounding text.

8. Line 301: Revise to "translocation of"

9. Line 305: Spell out "Histidine". "His" is easily mistaken for the pronoun at the start of the sentence.

10. Line 363: "the Bacteroides organism" is redundant in this sentence.

11. Line 394: Perhaps it is worth stating explicitly that salivary mucins would in fact be expected to transit the GI tract as a normal consequence of eating. As such, all members of the HGM/HCM might be anticipated to encounter this substrate.

12. Line 397-399, perhaps elsewhere: Is it possible that this protein requires 6S-GlcNAc to be presented on a polypeptide for

maximum activity? I maybe missing something here, but in general (e.g. Fig. 3) substrate specificity seems to be considered only for free mono/oligosaccharides.

13. Line 401: "Gram" should be capitalised as it is a surname.

14. In the legends for Fig. 5d and Fig. 6a, please clarify which structure(s) is experimental and which is modeled (and if so, how).

End of comments.

****

Version 1:

Reviewer comments:

Reviewer #1

(Remarks to the Author)
Thank you for your detailed responses. Most of the data you present is of a high standard, but unfortunately, I am not convinced about some of the data, some of the interpretations, or the answers provided on some of the issues raised first time.

My re-queries are:
1) The TLC in Fig2b – this is the core evidence of 6'S-LewisA/X activity, which is a really important element to the novelty. In my opinion the assays and TLCs need re-running. The two furthest left spots for the top TLC (control and +0121) - where have the blue and pink spots gone in the +0121 sample? How does this weird result relate to your response about the substrate being commercially acquired? They look like different substrates on that TLC. The bottom TLC is also not a clean result.
2) The lack of reproducibility for colonic mucin. I agree that this is a difficult procedure, but how can the results from one extraction be considered representative and how is anyone supposed to reproduce the work?
3) I am not sure how you have concluded increased lag phases for the 0490-491 (line 365). The lags are comparable to the monosaccharide control. They also reach the same OD, suggesting the same amount of substrate can be accessed. An increased lag suggest a response delay to substrate (both mucin and monosaccharides), not a substrate access issue. I suggest removing the conclusion of any phenotype.

Furthermore, some of your conclusions do not consider the full picture and from my perspective go too far or are incorrect. For example:
Line 146 - I would say that the growth profiles on the different substrates suggest (not reveal) substrate preferences - you have not directly confirmed this in your study.
Line 160 – an increase in the expression of the 0627 gene has been observed previously on PGM in the Desai, Ottman, Davey, and Bakshani papers, so this conclusion is not convincing. It is difficult to accept your conclusion if these publications are taken into consideration.
Line 57 and 377 – I do not think your results support this conclusion directly
Line 385 – there is no evidence that the GH16 enzymes only fit a cross-feeding role. The Davey paper shows a decrease in growth for the 2108 Tn mutant, for example. Akker must be able to grow on oligos to an extent as you show with your own data - Figure 1b.

Line 50 - A. muciniphilainiphila typo
Line 52 – what rare adaptations?
Figure 6 – the figure has changed but the legend was not updated. I am not convinced that both constructs were pulled down – it's mostly in the supernatant and there are thin lines for the wash an pellet? Furthermore, with the new data in b, the conclusions about binding are unclear as the 0953 is smaller then the BSA bands – is there another protein you could use as a comparison?

Reviewer #2

(Remarks to the Author)
The authors kindly answered all my questions/suggestions satisfactorily.

Reviewer #3

(Remarks to the Author)
I reviewed the previous version of this manuscript, and returned comments regarding the need for more evidence to support mucin substrate growth preferences. I also had comments regarding the overall clarity of the manuscript (also raised by Rev1).

This new version appears to be improved in terms of clarity. There are new gel affinity data regarding the interaction of Amuc0953 with gpcMO in response to one of my queries, and my other questions are also dealt with.

I am broadly happy for this version to go ahead, with some (minor) comments.

- Regarding the use of thermal shifts to infer binding affinities (originally Fig 2c, now Ext Fig 3b). I stand by my original assertion that this is not a solid interpretation of the data. To be clear, my specific issue is with the claim of measuring affinity.

The authors are correct that thermal shift measures stabilization of the protein by the ligand, and that this is indicative of an interaction. However, there appears to be conflation between the magnitude of the stabilization effect, and affinity of the ligand that is creating the stabilization (i.e. efficacy vs potency, to borrow terms from pharmacology).

I agree that larger stabilization magnitudes are likely to correlate with a better ligand affinities, as there is more binding/stabilization energy, but I do not believe this relationship is ironclad (may be ligand entropic/solvation effects on ligand that affect binding affinity, without necessarily stabilizing protein etc etc).

The thermal shift data in Ext Fig 3b shows 6S-Gal stabilize Amuc0121 with higher magnitude, and from 0.1 mM ligand. However, since saturating ligand concentrations where stabilization plateaus were never reached, it is difficult to be certain about affinity.

Better phrasing would just be that Amuc0121 shows lower thermal shift upon 6S-LewisA binding, indicative of a weaker, less stabilizing interaction.

Some other issues that will probably come out in editing:
- Fig 6 – caption doesn't match figure

- Abstract – A. muciniphilainiphila

- L158 - gpcMO samples did show that the mucin glycoprotease Amuc0627 was highly upregulated. The text refers to Fig g, which is a volcano plot. However, it is not shown in this plot where Amuc0627 is, and only GHs and sulfatases are highlighted. Annotation should be made more clearly in the figure (and if applicable, related volcano plots in Ext Fig 1)

Reviewer #4

(Remarks to the Author)
The authors have convincingly addressed all of my previous comments. Only minor typographical issues remain in this version:

- Line 50: "A. muciniphilainiphila"? Line 56: "monosaccharode"? Perhaps several of the authors, including the two corresponding authors, could proofread the text with more care.
- Lines 96-100: This sentence is grammatically challenging and unclear.
- Lines 95-96, with reference to my comment 2 on the previous version: In light of the authors' response, it would be most appropriate to insert a citation to the SulfAtlas database (current ref. 15) immediately after "...encodes 10 S1 carbohydrate sulfatases, classified into 6 subfamilies,"
- Line 100: "has shown that for"?
- Line 110: Change to "...to reveal that glycoprotein fragments,"
- Line 113: Change to "sulfatase-linked, mucin-binding" All similar noun-verb conjugates used as adjectives should be similarly hyphenated throughout the text, to improve readability.

End of comments.

Decision Letter:

29th January 2026

Dear Dr Cartmell,

Thank you for your patience while your manuscript "The role of Akkermansia muciniphila sulfatases in colonic mucin utilisation" was under peer review at Nature Microbiology. It has now been seen by our referees, whose expertise and comments you will find at the end of this email. In the light of their advice, we have decided that we cannot offer to publish your manuscript in Nature Microbiology.

From the reports, you will see that while they find your work of interest, there are still outstanding technical concerns and concerns regarding the robustness of evidence supporting some important claims made in the study. Unfortunately, these criticisms are sufficiently important as to preclude publication of your work in Nature Microbiology.

Although we cannot offer to publish your manuscript, I have discussed your manuscript and the reviewers' comments with my colleague Javier Martinez Vesga at Nature Communications. He would be happy to send a new revised manuscript that addresses the outstanding concerns back to the reviewers -when available-.

Should you wish to have a revised paper re-reviewed at Nature Communications, please use the transfer link once the revisions are completed. Please provide a detailed point-by-point response file to all the reviewer´s comments and a Cover Letter summarizing the changes.

If there is anything you would like to discuss before transferring, please don't hesitate to contact Javier by e-mail (javier.martinez-vesga@nature.com).

To transfer your manuscript please use our manuscript transfer portal. You will not have to re-supply manuscript metadata and files, unless you wish to make modifications. For more information, please see our manuscript transfer FAQ page.

I am sorry that we cannot be more positive on this occasion, but hope that you find the referees' comments helpful when preparing your paper for resubmission elsewhere.

Yours sincerely,

****
Reviewer Expertise:

Referee #1: Microbial mucin degradation
Referee #2: Glycobiology, Enzymology
Referee #3: Glycobiology, Structural Biology
Referee #4: Biochemistry, Glycobiology

Reviewers Comments:

Reviewer #1 (Remarks to the Author):

Thank you for your detailed responses. Most of the data you present is of a high standard, but unfortunately, I am not convinced about some of the data, some of the interpretations, or the answers provided on some of the issues raised first time.

My re-queries are:
1) The TLC in Fig2b – this is the core evidence of 6'S-LewisA/X activity, which is a really important element to the novelty. In my opinion the assays and TLCs need re-running. The two furthest left spots for the top TLC (control and +0121) - where have the blue and pink spots gone in the +0121 sample? How does this weird result relate to your response about the substrate being commercially acquired? They look like different substrates on that TLC. The bottom TLC is also not a clean result.
2) The lack of reproducibility for colonic mucin. I agree that this is a difficult procedure, but how can the results from one extraction be considered representative and how is anyone supposed to reproduce the work?
3) I am not sure how you have concluded increased lag phases for the 0490-491 (line 365). The lags are comparable to the monosaccharide control. They also reach the same OD, suggesting the same amount of substrate can be accessed. An increased lag suggest a response delay to substrate (both mucin and monosaccharides), not a substrate access issue. I suggest removing the conclusion of any phenotype.

Furthermore, some of your conclusions do not consider the full picture and from my perspective go too far or are incorrect. For example:
Line 146 - I would say that the growth profiles on the different substrates suggest (not reveal) substrate preferences - you have not directly confirmed this in your study.
Line 160 – an increase in the expression of the 0627 gene has been observed previously on PGM in the Desai, Ottman, Davey, and Bakshani papers, so this conclusion is not convincing. It is difficult to accept your conclusion if these publications are taken into consideration.
Line 57 and 377 – I do not think your results support this conclusion directly
Line 385 – there is no evidence that the GH16 enzymes only fit a cross-feeding role. The Davey paper shows a decrease in growth for the 2108 Tn mutant, for example. Akker must be able to grow on oligos to an extent as you show with your own data - Figure 1b.

Line 50 - A. muciniphilainiphila typo
Line 52 – what rare adaptations?
Figure 6 – the figure has changed but the legend was not updated. I am not convinced that both constructs were pulled down – it's mostly in the supernatant and there are thin lines for the wash an pellet? Furthermore, with the new data in b, the conclusions about binding are unclear as the 0953 is smaller then the BSA bands – is there another protein you could use as a comparison?

Reviewer #2 (Remarks to the Author):

The authors kindly answered all my questions/suggestions satisfactorily.

Reviewer #3 (Remarks to the Author):

I reviewed the previous version of this manuscript, and returned comments regarding the need for more evidence to support mucin substrate growth preferences. I also had comments regarding the overall clarity of the manuscript (also raised by Rev1).

This new version appears to be improved in terms of clarity. There are new gel affinity data regarding the interaction of

Amuc0953 with gpcMO in response to one of my queries, and my other questions are also dealt with.

I am broadly happy for this version to go ahead, with some (minor) comments.

- Regarding the use of thermal shifts to infer binding affinities (originally Fig 2c, now Ext Fig 3b). I stand by my original assertion that this is not a solid interpretation of the data. To be clear, my specific issue is with the claim of measuring affinity.

The authors are correct that thermal shift measures stabilization of the protein by the ligand, and that this is indicative of an interaction. However, there appears to be conflation between the magnitude of the stabilization effect, and affinity of the ligand that is creating the stabilization (i.e. efficacy vs potency, to borrow terms from pharmacology).

I agree that larger stabilization magnitudes are likely to correlate with a better ligand affinities, as there is more binding/stabilization energy, but I do not believe this relationship is ironclad (may be ligand entropic/solvation effects on ligand that affect binding affinity, without necessarily stabilizing protein etc etc).

The thermal shift data in Ext Fig 3b shows 6S-Gal stabilize Amuc0121 with higher magnitude, and from 0.1 mM ligand. However, since saturating ligand concentrations where stabilization plateaus were never reached, it is difficult to be certain about affinity.

Better phrasing would just be that Amuc0121 shows lower thermal shift upon 6S-LewisA binding, indicative of a weaker, less stabilizing interaction.

Some other issues that will probably come out in editing:
- Fig 6 – caption doesn't match figure

- Abstract – A. muciniphilainiphila

- L158 - gpcMO samples did show that the mucin glycoprotease Amuc0627 was highly upregulated. The text refers to Fig g, which is a volcano plot. However, it is not shown in this plot where Amuc0627 is, and only GHs and sulfatases are highlighted. Annotation should be made more clearly in the figure (and if applicable, related volcano plots in Ext Fig 1)

Reviewer #4 (Remarks to the Author):

The authors have convincingly addressed all of my previous comments. Only minor typographical issues remain in this version:

- Line 50: "A. muciniphilainiphila"? Line 56: "monosaccharode"? Perhaps several of the authors, including the two corresponding authors, could proofread the text with more care.
- Lines 96-100: This sentence is grammatically challenging and unclear.
- Lines 95-96, with reference to my comment 2 on the previous version: In light of the authors' response, it would be most appropriate to insert a citation to the SulfAtlas database (current ref. 15) immediately after "...encodes 10 S1 carbohydrate sulfatases, classified into 6 subfamilies,"
- Line 100: "has shown that for"?
- Line 110: Change to "...to reveal that glycoprotein fragments,"
- Line 113: Change to "sulfatase-linked, mucin-binding" All similar noun-verb conjugates used as adjectives should be similarly hyphenated throughout the text, to improve readability.

End of comments.

****

Version 2:

Decision Letter:

26th February 2026

Dear Dr Cartmell,

Thank you for your letter asking us to reconsider our decision on your Article entitled "The role of Akkermansia muciniphila sulfatases in colonic mucin utilisation". After careful consideration we have decided that we would be willing to consider a revised version of your manuscript.

We, however, don't feel the need to replace referee 1 but would ask other referees to share their opinion on the response/comments to referee 1.

Along with your revised manuscript, you should also submit a separate point-by-point response to all of the concerns raised by

the referees, in each case describing what changes have been made to the manuscript or, alternatively, if no action has been taken, providing a compelling argument for why that is the case. If we feel that a substantial attempt has been made to address the referees' comments, this response will be sent back to the referees - along with the revised manuscript - so that they can judge whether their concerns have been addressed satisfactorily or otherwise.

I should stress, however, that we would be reluctant to trouble our referees again unless we thought that their comments had been addressed in full.

When revising your paper:

- ensure it complies with our format requirements for Letters as set out in our guide to authors at www.nature.com/nmicrobiol/authors/index.html

- state in a cover note the length of the text, methods and legends; the number of references and the number of display items.

Please ensure that all correspondence is marked with your Nature Microbiology reference number in the subject line.

Please use the following link to submit your revised manuscript:

Link Redacted

We hope to receive your revised paper within four weeks. If you cannot send it within this time, please let us know so that we can close your file. In this event, we will still be happy to reconsider your paper at a later date so long as nothing similar has been accepted for publication at Nature Microbiology or published elsewhere in the meantime. Should you miss the four-week deadline and your paper is eventually published, the received date will be that of the revised, not the original, version.

I would appreciate it if you could tell me if you think you will be able to submit a revised manuscript, and also the likely timescale.

I look forward to hearing from you soon.

Yours sincerely,

Version 3:

Reviewer comments:

Reviewer #1

(Remarks to the Author)

Reviewer #3

(Remarks to the Author)
Please note I have also attached these comments in a word file, which contains font and color formatting to help with clarity.

I have been asked to mediate the exchange between the authors and Reviewer 1, following their critical review of the previous version of the manuscript.

In summary, I am mostly in agreement with the authors, and I feel Rev1's criticisms are adequately addressed in most cases. However, there are some technical issues on quality where I feel Rev1 is justified in their criticism.

Point by point:

1 - Author/reviewer exchange
The TLC in Fig2b – this is the core evidence of 6'S-LewisA/X activity, which is a really important element to the novelty. In my opinion the assays and TLCs need re-running. The two furthest left spots for the top TLC (control and +0121) - where have the blue and pink spots gone in the +0121 sample? How does this weird result relate to your response about the substrate being commercially acquired? They look like different substrates on that TLC. The bottom TLC is also not a clean result.

RESPONSE: The activity of Amuc0121 on 6S-Lewis antigens is supported by TLC, HPAEC and TSA. We do not rely solely on this TLC result; the substrate used in these assays was also co-crystallised with the protein revealing the atomic details of the protein-substrate interaction. The comment 'they look like different substrates' is curious as when an enzyme acts on a substrate it produces a product and this must look different from the start material i.e. spots for substrate disappear and new spots relating to product appear. The pink and blue spots are the substrate.

Focusing more on the 6S-LewisA; it is true the control has three spots, two of which (the blue and pink) respond to Amuc0121 to produce a single spot, meaning that they produce the same product upon removal of the O6 linked sulfate group. The 6S-LewisA substrate is unresponsive to the fucosidase BT4136 and galactosidase BT4241 which is consistent with them being active on LewisA but not 6S-LewisA. Upon addition of Amuc0121, and both BT4136 and BT4241, the products of galactose, fucose, and N-acetylglucosamine are produced, consistent with the removal of O6 sulfation thereby allowing access to the LewisA substrate. The data are corroborated with HPAEC analysis.

The 6S-LewisX TLC is a cleaner result and clearly shows the same trend as the 6S-LewisA data; there is a definitive shift upon the addition of Amuc0121, and Amuc0121 allows access of the galactosidase and fucosidase to degrade the substrate. Again, these data are backed up by HPAEC analysis.

The sulfatase, galactosidases, and fucosidases used above are stereospecific, further confirming the nature of the substrate. The reviewer should/will be aware of this as their activities are published; https://www.nature.com/articles/s41564-019-0466-x, https://www.nature.com/articles/s41467-025-58660-2, and https://doi.org/10.1042/EBC20220158.

Finally, these sulfated Lewis substrates are chemically synthesised and commercially available (from Dextra) with the presence of the 6S-Lewis structures of each substrate confirmed in a QC report. We would be willing to ask Dextra to supply additional copies of these QC reports.

1 - My response
I side with Rev1 here. The TLCs look strange, or at the very least are poorly explained.
- Fig2b top panel (6'S-Lewis A) - it is not explained why the control lane has 3 spots, when it is a (presumably) homogenous pure compound from Dextra. Without further context, I would expect pure, stable compounds to only give a single spot on TLC (I also see no reason why 6'S-Lewis A should not be stable on silica). The result is made more problematic as the top spot appears to be the same Rf and colour as GlcNAc only, suggesting contamination. If this discrepancy can be explained, then I think the rest of the TLC makes sense.
- I also agree with Rev1 that the TLC quality is poor. For example, the +Amuc0121/BT4136/BT4142 lane where we would expect to see digest products, the spot corresponding to Fuc is basically not visible. I suggest more material needs to be loaded, or another stain used. There are also issues with annotation - is BT4142 the b1,3 galactosidase, or BT4243 as per the figure caption text?
- Fig2b bottom panel (6'S-Lewis X) - suffers from the same problem as the top panel, but the quality looks even worse. Spots in the +Amuc0121/BT4136/BT0461 lane are basically not visible.

Action points
- Issues with TLC spots should ideally be resolved or clarified. QC reports from manufacturers would help, or else the authors should run their own LCMS or NMR analyses to confirm the substrates are pure.
- I appreciate the authors' response that there are multiple pieces of evidence for the Amuc0121 digestion profile. In particular, HPAEC data seems more convincing than the TLC. Why is this not in the main text?

2 - Author/reviewer exchange
The lack of reproducibility for colonic mucin. I agree that this is a difficult procedure, but how can the results from one extraction be considered representative and how is anyone supposed to reproduce the work?

RESPONSE: We have robustly benchmarked our work by providing mass spectrometry, NMR, and glycoprotein gel staining of the gpcMO material. For those wishing to reproduce our gpcMO substrate, we have provided full details: they can vary the reaction conditions until material is obtained that matches these bench marks. Details of the entire procedure, and our subsequent analysis, has been provided in the methods and results. This provides complete transparency to allow replication by other groups.

Finally, the extractions are from 10 porcine distal colons ~1 meter in length but this has little effect on the generation of gpcMOs/cMOs. This is done after extraction and is a separate process, as detailed in the methods.

2 - My response
Tricky, but I just about side with the authors here. I see the gpcMO result as an initial point of serendipity that drove subsequent analysis of the sulfatases, which is the main story of the paper. As the authors state, the gpcMO extract is benchmarked by several analytical techniques.

3 - Author/reviewer exchange
I am not sure how you have concluded increased lag phases for the 0490-491 (line 365). The lags are comparable to the monosaccharide control. They also reach the same OD, suggesting the same amount of substrate can be accessed. An increased lag suggest a response delay to substrate (both mucin and monosaccharides), not a substrate access issue. I suggest removing the conclusion of any phenotype.

RESPONSE: The reviewer has succinctly defined the phenotype; the 0490-491 mutant has a defect (increased lag phase) on all substrates tested compared to wildtype. We have not suggested any role of substrate access and stated the effect was observed across all substrates thus not mucin specific. We have placed the excerpt from the manuscript below:

'When grown on sPGM and gpcMOs, most mutants displayed no growth differences to wildtype except amuc0490-491::tn (which had an increased lag phase on both sPGM and gpcMOs) and amuc1182::tn (which had an increase in lag phase on sPGM). The

Tn insertion in amuc0490-491::tn spans both genes and the defect observed here is likely to be linked to amuc0490, since it is observed across all substrates tested.'

We have revised this to avoid confusion:

'On sPGM and gpcMOs most mutants displayed no growth differences to wildtype except amuc0490-491::tn and amuc1182::tn. The mutant amuc1182::tn had an increased lag phase only on sPGM, while the amuc0490-491::tn mutant show an increased lag on all substrates including GlcNAc. The Tn insertion in amuc0490-491::tn spans both genes and the defect observed here is likely linked to amuc0490 (a putative inner membrane protein of unknown function) and is a global defect not specific to mucin or the sulfatase Amuc04903S-Gal/GalNAc.'

3 - My response
I side with the authors. I think they are sufficiently clear about the phenotype.

4 - Author/reviewer exchange
Furthermore, some of your conclusions do not consider the full picture and from my perspective go too far or are incorrect. For example:
Line 146 - I would say that the growth profiles on the different substrates suggest (not reveal) substrate preferences - you have not directly confirmed this in your study.

RESPONSE: We strongly disagree with this comment. Growth profiles are the ultimate test of whether a bacterial strain prefers one substrate over another, and can be more informative than the sum of various reductionist enzyme assays. While growth experiments are simple, they reveal important biological information in the context of the whole cellular system.

4 - My response
Side with authors.

5 - Author/reviewer exchange
Line 160 – an increase in the expression of the 0627 gene has been observed previously on PGM in the Desai, Ottman, Davey, and Bakshani papers, so this conclusion is not convincing. It is difficult to accept your conclusion if these publications are taken into consideration.

RESPONSE: We have removed this point as it is a minor contribution to the paper which primarily focuses on how A. muciniphila grows on, and deploys carbohydrate sulfatases, to access colonic mucins. We accept the Amuc_0627 up regulation on gastric mucins is significant.

The revised sentence reads as such:

'When comparing gpcMOs to sPGM samples only three sulfatases (Amuc0491, Amuc1655 and Amuc1182) are more upregulated (Figure 1f and g). Overall, the different gastric and colonic mucin substrates did not lead to large differences in sulfatase expression (Extended data figure 1d and Supplementary dataset 3).'

5 - My response
N/A – issue has been dealt with

6 - Author/reviewer exchange
Line 57 and 377 – I do not think your results support this conclusion directly
RESPONSE: We presume this is in response to the growth phenotypes in figure 1 showing A. muciniphila has a preference for glycopeptide/proteins of a specific size, and is a repetition of the same point above about microbial growth curves. The results are clear and accepted by the other 3 expert reviewers. The confusion of the reviewer likely relates to their misinterpretation of microbial growth data as already detailed above.

6 - My response
N/A – related to the above point

7 - Author/reviewer exchange
Line 385 – there is no evidence that the GH16 enzymes only fit a cross-feeding role. The Davey paper shows a decrease in growth for the 2108 Tn mutant, for example. Akker must be able to grow on oligos to an extent as you show with your own data - Figure 1b.

RESPONSE: We note this is part of our discussion and not the results. A. muciniphila does not grow on highly sulfated colonic mucin oligosaccharides as confirmed by our data and Luis et al (https://www.nature.com/articles/s41586-021-03967-5#Sec28). Our data shows it grows poorly on low sulfation gastric mucin oligosaccharides. A. muciniphila has cell surface GH16 enzymes that produce oligosaccharides from sulfated mucins (https://www.nature.com/articles/s41467-020-17847-5) and A. muciniphila cannot use these, therefore, we suggest that they may be used by other organisms. This does not seem a large intellectual leap. Amuc_2106 is predicted to be a periplasmic enzyme which renders this reviewers' comparison invalid in the context of cell surface activities.

7 - My response
This is not my core expertise, but I feel like this is a reasonable thing to mention in the discussion.

8 - Author/reviewer exchange
Line 50 - A. muciniphilainiphila typo
RESPONSE: This has been corrected

8 - My response
N/A – issue has been dealt with

9 - Author/reviewer exchange
Line 52 – what rare adaptations?
RESPONSE: These are mutations that occur infrequently and afford the sulfatases tailored activities. This analysis was conducted by examining ~100s of sequences, adjusted for taxonomic bias, across the sulfatase subfamilies and looking at the frequency of specific amino acids at positions of interest; thus identifying the A. muciniphila sulfatases have rare adaptations in the active site explaining there specificity to disaccharide substrates.

9 - My response
N/A – issue has been dealt with

10 - Author/reviewer exchange
Figure 6 – the figure has changed but the legend was not updated. I am not convinced that both constructs were pulled down – it's mostly in the supernatant and there are
thin lines for the wash an pellet?

RESPONSE: We were remiss in not updating the legend, this has now been done. However, it is common to have unbound protein in the wash because an excess of protein (10 M) is used to saturate the mucin. The key is that both Amuc0953P395 and Amuc0953P490 are pulled down in the pellet, while BSA is not (Figure 6a (i)). A ten-fold enrichment of the pellet shows that Amuc0953P395 and Amuc0953P490 are enriched, and BSA is not (Figure 6a (ii)). Furthermore, this interaction is only observed with PCM and not the control polysaccharide, cellulose. Finally, western blots were run with an anti-His tag antibody to confirm the presence or absence of the recombinant protein.

10 - My response
I side with the authors. The pulldown is not designed to be quantitative, so leftovers in the supernatant are not unexpected. The observation that the Amus0953 constructs are pulled down by the PCM, not the BSA, and not in the cellulose controls, is consistent with the claimed interpretations

11 - Author/reviewer exchange
Furthermore, with the new data in b, the conclusions about binding are unclear as the 0953 is smaller then the BSA bands – is there another protein you could use as a comparison?

RESPONSE: This appears to be a misunderstanding of the nature of the assay; only the level of retardation matters relative to the control protein BSA rather than the absolute mass of the proteins. Amuc0953P395 is clearly retarded by the colonic and not the gastric substrate. BSA is the gold standard control for these assays, please see the following papers:

https://pubmed.ncbi.nlm.nih.gov/27298375/
https://www.sciencedirect.com/science/article/pii/S0014579315005888
https://www.nature.com/articles/srep19392
https://www.nature.com/articles/s41467-022-28310-y.

11 - My response
Overall I side with the authors, although the figure could be clearer. Annotation would be useful, especially e.g. MW markers that each gel could be benchmarked to. The fact that BSA itself looks like it has run differently on the gpcMO gel likely adds to confusion.

Decision Letter:

25th March 2026

*Please ensure you delete the link to your author homepage in this e-mail if you wish to forward it to your co-authors.

Dear Alan,

Thank you for your patience while your manuscript "The role of Akkermansia muciniphila sulfatases in colonic mucin utilisation"

was under peer-review at Nature Microbiology. You will see from the comments of the referees below that a few outstanding issues remain. We are very interested in the possibility of publishing your study in Nature Microbiology, but would like to consider your response to these concerns in the form of a revised manuscript before we make a final decision on publication.

In particular, you will see that R3 worked as a sort of adjudicator and found that most of the concerns of R1 are well addressed but they do share the same concern as R1 on the TLC plates which we would like you to address.

We are committed to providing a fair and constructive peer-review process. Do not hesitate to contact us if there are specific requests from the reviewers that you believe are technically impossible or unlikely to yield a meaningful outcome.

If you have not done so already please begin to revise your manuscript so that it conforms to our Article format instructions at http://www.nature.com/nmicrobiol/info/final-submission/

The usual length limit for a Nature Microbiology Article is six display items (figures or tables) and 3,000 words. We have some flexibility, and can allow a revised manuscript at 3,500 words, but please consider this a firm upper limit. There is a trade-off of ~250 words per display item, so if you need more space, you could move a Figure or Table to Supplementary Information.

Some reduction could be achieved by focusing any introductory material and moving it to the start of your opening ‘bold’ paragraph, whose function is to outline the background to your work, describe in a sentence your new observations, and explain your main conclusions. The discussion should also be limited. Methods should be described in a separate section following the discussion, we do not place a word limit on Methods.

Nature Microbiology titles should give a sense of the main new findings of a manuscript, and should not contain punctuation. Please keep in mind that we strongly discourage active verbs in titles, and that they should ideally fit within 90 characters each (including spaces).

We strongly support public availability of data. Please place the data used in your paper into a public data repository, if one exists, or alternatively, present the data as Source Data or Supplementary Information. If data can only be shared on request, please explain why in your Data Availability Statement, and also in the correspondence with your editor. For some data types, deposition in a public repository is mandatory - more information on our data deposition policies and available repositories can be found at https://www.nature.com/nature-research/editorial-policies/reporting-standards#availability-of-data.

Please include a data availability statement as a separate section after Methods but before references, under the heading "Data Availability”. This section should inform readers about the availability of the data used to support the conclusions of your study. This information includes accession codes to public repositories (data banks for protein, DNA or RNA sequences, microarray, proteomics data etc…), references to source data published alongside the paper, unique identifiers such as URLs to data repository entries, or data set DOIs, and any other statement about data availability. At a minimum, you should include the following statement: “The data that support the findings of this study are available from the corresponding author upon request”, mentioning any restrictions on availability. If DOIs are provided, we also strongly encourage including these in the Reference list (authors, title, publisher (repository name), identifier, year). For more guidance on how to write this section please see: http://www.nature.com/authors/policies/data/data-availability-statements-data-citations.pdf

To improve the accessibility of your paper to readers from other research areas, please pay particular attention to the wording of the paper’s opening bold paragraph, which serves both as an introduction and as a brief, non-technical summary in about 150 words. If, however, you require one or two extra sentences to explain your work clearly, please include them even if the paragraph is over-length as a result. The opening paragraph should not contain references. Because scientists from other sub-disciplines will be interested in your results and their implications, it is important to explain essential but specialised terms concisely. We suggest you show your summary paragraph to colleagues in other fields to uncover any problematic concepts.

If your paper is accepted for publication, we will edit your display items electronically so they conform to our house style and will reproduce clearly in print. If necessary, we will re-size figures to fit single or double column width. If your figures contain several parts, the parts should form a neat rectangle when assembled. Choosing the right electronic format at this stage will speed up the processing of your paper and give the best possible results in print. We would like the figures to be supplied as vector files - EPS, PDF, AI or postscript (PS) file formats (not raster or bitmap files), preferably generated with vector-graphics software (Adobe Illustrator for example). Please try to ensure that all figures are non-flattened and fully editable. All images should be at least 300 dpi resolution (when figures are scaled to approximately the size that they are to be printed at) and in RGB colour format. Please do not submit Jpeg or flattened TIFF files. Please see also 'Guidelines for Electronic Submission of Figures' at the end of this letter for further detail.

Figure legends must provide a brief description of the figure and the symbols used, within 350 words, including definitions of any error bars employed in the figures.

When submitting the revised version of your manuscript, please pay close attention to our href="https://www.nature.com/nature-research/editorial-policies/image-integrity">Digital Image Integrity Guidelines. and to the following points below:

-- that unprocessed scans are clearly labelled and match the gels and western blots presented in figures.
-- that control panels for gels and western blots are appropriately described as loading on sample processing controls
-- all images in the paper are checked for duplication of panels and for splicing of gel lanes.

EXTENDED DATA FIGURES

When re-submitting your manuscript, please ensure that any supplementary figures and tables that are crucial to the manuscript's conclusions are converted into Extended Data figures and tables to increase visibility of these data. Extended Data figures and tables are online-only (present in the online PDF and full-text HTML versions of the paper), peer-reviewed display items that provide essential background to the article but are not included in the main article due to space constraints. A maximum of ten Extended Data display items (figures and tables) is permitted.

Finally, please ensure that you retain unprocessed data and metadata files after publication, ideally archiving data in perpetuity, as these may be requested during the peer review and production process or after publication if any issues arise.

Please include a statement before the acknowledgements naming the author to whom correspondence and requests for materials should be addressed.

Finally, we require authors to include a statement of their individual contributions to the paper -- such as experimental work, project planning, data analysis, etc. -- immediately after the acknowledgements. The statement should be short, and refer to authors by their initials. For details please see the Authorship section of our joint Editorial policies at http://www.nature.com/authors/editorial_policies/authorship.html

When revising your paper:

* include a point-by-point response to any editorial suggestions and to our referees. Please include your response to the editorial suggestions in your cover letter, and please upload your response to the referees as a separate document.

* ensure it complies with our format requirements for Letters as set out in our guide to authors at www.nature.com/nmicrobiol/info/gta/

* state in a cover note the length of the text, methods and legends; the number of references; number and estimated final size of figures and tables

* resubmit electronically if possible using the link below to access your home page:

Link Redacted

*This url links to your confidential homepage and associated information about manuscripts you may have submitted or be reviewing for us. If you wish to forward this e-mail to co-authors, please delete this link to your homepage first.

Please ensure that all correspondence is marked with your Nature Microbiology reference number in the subject line.

Nature Microbiology is committed to improving transparency in authorship. As part of our efforts in this direction, we are now requesting that all authors identified as 'corresponding author' on published papers create and link their Open Researcher and Contributor Identifier (ORCID) with their account on the Manuscript Tracking System (MTS), prior to acceptance. This applies to primary research papers only. ORCID helps the scientific community achieve unambiguous attribution of all scholarly contributions. You can create and link your ORCID from the home page of the MTS by clicking on 'Modify my Springer Nature account'. For more information please visit please visit www.springernature.com/orcid.

We hope to receive your revised paper within three weeks. If you cannot send it within this time, please let us know.

We look forward to hearing from you soon.

Yours sincerely,

****

Reviewers Comments:

Reviewer #3 (Remarks to the Author):

Please note I have also attached these comments in a word file, which contains font and color formatting to help with clarity.

I have been asked to mediate the exchange between the authors and Reviewer 1, following their critical review of the previous version of the manuscript.

In summary, I am mostly in agreement with the authors, and I feel Rev1's criticisms are adequately addressed in most cases. However, there are some technical issues on quality where I feel Rev1 is justified in their criticism.

Point by point:

1 - Author/reviewer exchange
The TLC in Fig2b – this is the core evidence of 6'S-LewisA/X activity, which is a really important element to the novelty. In my opinion the assays and TLCs need re-running. The two furthest left spots for the top TLC (control and +0121) - where have the blue and pink spots gone in the +0121 sample? How does this weird result relate to your response about the substrate being commercially acquired? They look like different substrates on that TLC. The bottom TLC is also not a clean result.

RESPONSE: The activity of Amuc0121 on 6S-Lewis antigens is supported by TLC, HPAEC and TSA. We do not rely solely on this TLC result; the substrate used in these assays was also co-crystallised with the protein revealing the atomic details of the protein-substrate interaction. The comment 'they look like different substrates' is curious as when an enzyme acts on a substrate it produces a product and this must look different from the start material i.e. spots for substrate disappear and new spots relating to product appear. The pink and blue spots are the substrate.

Focusing more on the 6S-LewisA; it is true the control has three spots, two of which (the blue and pink) respond to Amuc0121 to produce a single spot, meaning that they produce the same product upon removal of the O6 linked sulfate group. The 6S-LewisA substrate is unresponsive to the fucosidase BT4136 and galactosidase BT4241 which is consistent with them being active on LewisA but not 6S-LewisA. Upon addition of Amuc0121, and both BT4136 and BT4241, the products of galactose, fucose, and N-acetylglucosamine are produced, consistent with the removal of O6 sulfation thereby allowing access to the LewisA substrate. The data are corroborated with HPAEC analysis.

The 6S-LewisX TLC is a cleaner result and clearly shows the same trend as the 6S-LewisA data; there is a definitive shift upon the addition of Amuc0121, and Amuc0121 allows access of the galactosidase and fucosidase to degrade the substrate. Again, these data are backed up by HPAEC analysis.

The sulfatase, galactosidases, and fucosidases used above are stereospecific, further confirming the nature of the substrate. The reviewer should/will be aware of this as their activities are published; https://www.nature.com/articles/s41564-019-0466-x, https://www.nature.com/articles/s41467-025-58660-2, and https://doi.org/10.1042/EBC20220158.

Finally, these sulfated Lewis substrates are chemically synthesised and commercially available (from Dextra) with the presence of the 6S-Lewis structures of each substrate confirmed in a QC report. We would be willing to ask Dextra to supply additional copies of these QC reports.

1 - My response
I side with Rev1 here. The TLCs look strange, or at the very least are poorly explained.
- Fig2b top panel (6'S-Lewis A) - it is not explained why the control lane has 3 spots, when it is a (presumably) homogenous pure compound from Dextra. Without further context, I would expect pure, stable compounds to only give a single spot on TLC (I also see no reason why 6'S-Lewis A should not be stable on silica). The result is made more problematic as the top spot appears to be the same Rf and colour as GlcNAc only, suggesting contamination. If this discrepancy can be explained, then I think the rest of the TLC makes sense.
- I also agree with Rev1 that the TLC quality is poor. For example, the +Amuc0121/BT4136/BT4142 lane where we would expect to see digest products, the spot corresponding to Fuc is basically not visible. I suggest more material needs to be loaded, or another stain used. There are also issues with annotation - is BT4142 the b1,3 galactosidase, or BT4243 as per the figure caption text?
- Fig2b bottom panel (6'S-Lewis X) - suffers from the same problem as the top panel, but the quality looks even worse. Spots in the +Amuc0121/BT4136/BT0461 lane are basically not visible.

Action points
- Issues with TLC spots should ideally be resolved or clarified. QC reports from manufacturers would help, or else the authors should run their own LCMS or NMR analyses to confirm the substrates are pure.
- I appreciate the authors' response that there are multiple pieces of evidence for the Amuc0121 digestion profile. In particular, HPAEC data seems more convincing than the TLC. Why is this not in the main text?

2 - Author/reviewer exchange
The lack of reproducibility for colonic mucin. I agree that this is a difficult procedure, but how can the results from one extraction be considered representative and how is anyone supposed to reproduce the work?

RESPONSE: We have robustly benchmarked our work by providing mass spectrometry, NMR, and glycoprotein gel staining of the gpcMO material. For those wishing to reproduce our gpcMO substrate, we have provided full details: they can vary the reaction conditions until material is obtained that matches these bench marks. Details of the entire procedure, and our subsequent analysis, has been provided in the methods and results. This provides complete transparency to allow replication by other groups.

Finally, the extractions are from 10 porcine distal colons ~1 meter in length but this has little effect on the generation of gpcMOs/cMOs. This is done after extraction and is a separate process, as detailed in the methods.

2 - My response
Tricky, but I just about side with the authors here. I see the gpcMO result as an initial point of serendipity that drove subsequent analysis of the sulfatases, which is the main story of the paper. As the authors state, the gpcMO extract is benchmarked by several analytical techniques.

3 - Author/reviewer exchange
I am not sure how you have concluded increased lag phases for the 0490-491 (line 365). The lags are comparable to the monosaccharide control. They also reach the same OD, suggesting the same amount of substrate can be accessed. An increased lag suggest a response delay to substrate (both mucin and monosaccharides), not a substrate access issue. I suggest removing the conclusion of any phenotype.

RESPONSE: The reviewer has succinctly defined the phenotype; the 0490-491 mutant has a defect (increased lag phase) on all substrates tested compared to wildtype. We have not suggested any role of substrate access and stated the effect was observed across all substrates thus not mucin specific. We have placed the excerpt from the manuscript below:

'When grown on sPGM and gpcMOs, most mutants displayed no growth differences to wildtype except amuc0490-491::tn (which had an increased lag phase on both sPGM and gpcMOs) and amuc1182::tn (which had an increase in lag phase on sPGM). The Tn insertion in amuc0490-491::tn spans both genes and the defect observed here is likely to be linked to amuc0490, since it is observed across all substrates tested.'

We have revised this to avoid confusion:

'On sPGM and gpcMOs most mutants displayed no growth differences to wildtype except amuc0490-491::tn and amuc1182::tn. The mutant amuc1182::tn had an increased lag phase only on sPGM, while the amuc0490-491::tn mutant show an increased lag on all substrates including GlcNAc. The Tn insertion in amuc0490-491::tn spans both genes and the defect observed here is likely linked to amuc0490 (a putative inner membrane protein of unknown function) and is a global defect not specific to mucin or the sulfatase Amuc04903S-Gal/GalNAc.'

3 - My response
I side with the authors. I think they are sufficiently clear about the phenotype.

4 - Author/reviewer exchange
Furthermore, some of your conclusions do not consider the full picture and from my perspective go too far or are incorrect. For example:
Line 146 - I would say that the growth profiles on the different substrates suggest (not reveal) substrate preferences - you have not directly confirmed this in your study.

RESPONSE: We strongly disagree with this comment. Growth profiles are the ultimate test of whether a bacterial strain prefers one substrate over another, and can be more informative than the sum of various reductionist enzyme assays. While growth experiments are simple, they reveal important biological information in the context of the whole cellular system.

4 - My response
Side with authors.

5 - Author/reviewer exchange
Line 160 – an increase in the expression of the 0627 gene has been observed previously on PGM in the Desai, Ottman, Davey, and Bakshani papers, so this conclusion is not convincing. It is difficult to accept your conclusion if these publications are taken into consideration.

RESPONSE: We have removed this point as it is a minor contribution to the paper which primarily focuses on how A. muciniphila grows on, and deploys carbohydrate sulfatases, to access colonic mucins. We accept the Amuc_0627 up regulation on gastric mucins is significant.

The revised sentence reads as such:

'When comparing gpcMOs to sPGM samples only three sulfatases (Amuc0491, Amuc1655 and Amuc1182) are more upregulated (Figure 1f and g). Overall, the different gastric and colonic mucin substrates did not lead to large differences in sulfatase expression (Extended data figure 1d and Supplementary dataset 3).'

5 - My response
N/A – issue has been dealt with

6 - Author/reviewer exchange
Line 57 and 377 – I do not think your results support this conclusion directly
RESPONSE: We presume this is in response to the growth phenotypes in figure 1 showing A. muciniphila has a preference for glycopeptide/proteins of a specific size, and is a repetition of the same point above about microbial growth curves. The results are clear and accepted by the other 3 expert reviewers. The confusion of the reviewer likely relates to their misinterpretation of microbial growth data as already detailed above.

6 - My response
N/A – related to the above point

7 - Author/reviewer exchange
Line 385 – there is no evidence that the GH16 enzymes only fit a cross-feeding role. The Davey paper shows a decrease in growth for the 2108 Tn mutant, for example. Akker must be able to grow on oligos to an extent as you show with your own data - Figure 1b.

RESPONSE: We note this is part of our discussion and not the results. A. muciniphila does not grow on highly sulfated colonic mucin oligosaccharides as confirmed by our data and Luis et al (https://www.nature.com/articles/s41586-021-03967-5#Sec28). Our data shows it grows poorly on low sulfation gastric mucin oligosaccharides. A. muciniphila has cell surface GH16 enzymes that produce oligosaccharides from sulfated mucins (https://www.nature.com/articles/s41467-020-17847-5) and A. muciniphila cannot use these, therefore, we suggest that they may be used by other organisms. This does not seem a large intellectual leap. Amuc_2106 is predicted to be a periplasmic enzyme which renders this reviewers' comparison invalid in the context of cell surface activities.

7 - My response
This is not my core expertise, but I feel like this is a reasonable thing to mention in the discussion.

8 - Author/reviewer exchange
Line 50 - A. muciniphilainiphila typo
RESPONSE: This has been corrected

8 - My response
N/A – issue has been dealt with

9 - Author/reviewer exchange
Line 52 – what rare adaptations?
RESPONSE: These are mutations that occur infrequently and afford the sulfatases tailored activities. This analysis was conducted by examining ~100s of sequences, adjusted for taxonomic bias, across the sulfatase subfamilies and looking at the frequency of specific amino acids at positions of interest; thus identifying the A. muciniphila sulfatases have rare adaptations in the active site explaining there specificity to disaccharide substrates.

9 - My response
N/A – issue has been dealt with

10 - Author/reviewer exchange
Figure 6 – the figure has changed but the legend was not updated. I am not convinced that both constructs were pulled down – it's mostly in the supernatant and there are
thin lines for the wash an pellet?

RESPONSE: We were remiss in not updating the legend, this has now been done. However, it is common to have unbound protein in the wash because an excess of protein (10 M) is used to saturate the mucin. The key is that both Amuc0953P395 and Amuc0953P490 are pulled down in the pellet, while BSA is not (Figure 6a (i)). A ten-fold enrichment of the pellet shows that Amuc0953P395 and Amuc0953P490 are enriched, and BSA is not (Figure 6a (ii)). Furthermore, this interaction is only observed with PCM and not the control polysaccharide, cellulose. Finally, western blots were run with an anti-His tag antibody to confirm the presence or absence of the recombinant protein.

10 - My response
I side with the authors. The pulldown is not designed to be quantitative, so leftovers in the supernatant are not unexpected. The observation that the Amus0953 constructs are pulled down by the PCM, not the BSA, and not in the cellulose controls, is consistent with the claimed interpretations

11 - Author/reviewer exchange
Furthermore, with the new data in b, the conclusions about binding are unclear as the 0953 is smaller then the BSA bands – is there another protein you could use as a comparison?

RESPONSE: This appears to be a misunderstanding of the nature of the assay; only the level of retardation matters relative to the control protein BSA rather than the absolute mass of the proteins. Amuc0953P395 is clearly retarded by the colonic and not the gastric substrate. BSA is the gold standard control for these assays, please see the following papers:

https://pubmed.ncbi.nlm.nih.gov/27298375/
https://www.sciencedirect.com/science/article/pii/S0014579315005888
https://www.nature.com/articles/srep19392
https://www.nature.com/articles/s41467-022-28310-y.

11 - My response
Overall I side with the authors, although the figure could be clearer. Annotation would be useful, especially e.g. MW markers that

each gel could be benchmarked to. The fact that BSA itself looks like it has run differently on the gpcMO gel likely adds to confusion.

****

Version 4:

Reviewer comments:

Reviewer #3

(Remarks to the Author)
My two main criticisms last time were to do with the quality of the TLC analysis for 6'S-Sia-LewisA/X digestion (Figure 2b), and annotation needed for the gel shift assay (Figure 6b).

TLC analysis - The rerun TLCs with new/cleaner substrate look ok, especially for Lewis A. The TLC for Lewis X still looks slightly weak, but the main findings are also confirmed by HPAEC, so as a package of results the conclusions seem sound. The HPAEC for Lewis A digest has been moved to the main text, which is good as these data are higher quality than the TLC.

The authors however still need to fix their annotation in the figure caption. For Figure 2d, the caption mentions BT4241, but the figure has BT4142. Which is correct? The caption also still mentions BT0461, despite the fact the relevant data have now been moved to extended data.

Annotation for gel shift assay - this is fine for me now.

Decision Letter:

Our ref: NMICROBIOL-25093383D

7th May 2026

Dear Alan,

Thank you for submitting your revised manuscript "The role of Akkermansia muciniphila sulfatases in colonic mucin utilisation" (NMICROBIOL-25093383D). It has now been seen by the original referees and their comments are below. The reviewers find that the paper has improved in revision, and therefore we'll be happy in principle to publish it in Nature Microbiology, pending minor revisions to satisfy the referees' final requests and to comply with our editorial and formatting guidelines.

If the current version of your manuscript is in a PDF format, please email us a copy of the file in an editable format (Microsoft Word or LaTex)-- we can not proceed with PDFs at this stage.

We are now performing detailed checks on your paper and will send you a checklist detailing our editorial and formatting requirements in about a week. Please do not upload the final materials and make any revisions until you receive this additional information from us.

Thank you again for your interest in Nature Microbiology Please do not hesitate to contact me if you have any questions.

Sincerely,

Reviewer #3 (Remarks to the Author):

My two main criticisms last time were to do with the quality of the TLC analysis for 6'S-Sia-LewisA/X digestion (Figure 2b), and annotation needed for the gel shift assay (Figure 6b).

TLC analysis - The rerun TLCs with new/cleaner substrate look ok, especially for Lewis A. The TLC for Lewis X still looks slightly weak, but the main findings are also confirmed by HPAEC, so as a package of results the conclusions seem sound. The HPAEC for Lewis A digest has been moved to the main text, which is good as these data are higher quality than the TLC.

The authors however still need to fix their annotation in the figure caption. For Figure 2d, the caption mentions BT4241, but the figure has BT4142. Which is correct? The caption also still mentions BT0461, despite the fact the relevant data have now been moved to extended data.

Annotation for gel shift assay - this is fine for me now.

Version 5:

Decision Letter:

10th June 2026

Dear Alan,

I am pleased to accept your Article "Unique adaptations in sulfatases underpin colonic mucin degradation by Akkermansia muciniphila" for publication in Nature Microbiology. Thank you for choosing Nature Microbiology to submit your work and many congratulations.

Over the next few weeks, your paper will be copyedited to ensure that it conforms to Nature Microbiology style. We look particularly carefully at the titles of all papers to ensure that they are relatively brief and understandable.

Once your paper is typeset, you will receive an email with a link to choose the appropriate publishing options for your paper and our Author Services team will be in touch regarding any additional information that may be required. Once your paper has been scheduled for online publication, the Nature press office will be in touch to confirm the details.

You may wish to make your media relations office aware of your accepted publication, in case they consider it appropriate to organize some internal or external publicity. Once your paper has been scheduled you will receive an email confirming the publication details. This is normally 3-4 working days in advance of publication. If you need additional notice of the date and time of publication, please let the production team know when you receive the proof of your article to ensure there is sufficient time to coordinate. Further information on our embargo policies can be found here: https://www.nature.com/authors/policies/embargo.html

After the grant of rights is completed, you will receive a link to your electronic proof via email with a request to make any corrections within 48 hours. If, when you receive your proof, you cannot meet this deadline, please inform us at rjsproduction@springernature.com immediately. You will not receive your proofs until the publishing agreement has been received through our system

Due to the importance of these deadlines, we ask you please us know now whether you will be difficult to contact over the next month. If this is the case, we ask you provide us with the contact information (email, phone and fax) of someone who will be able to check the proofs on your behalf, and who will be available to address any last-minute problems.

Acceptance of your manuscript is conditional on all authors' agreement with our publication policies (see https://www.nature.com/nmicrobiol/editorial-policies). In particular your manuscript must not be published elsewhere.

Authors may need to take specific actions to achieve compliance with funder and institutional open access mandates. If your research is supported by a funder that requires immediate open access (e.g. according to Plan S principles or the NIH public access policy) then you should select the gold OA route, and we will direct you to the compliant route where possible. Because authors warrant under our subscription licensing terms that they haven’t committed to licensing any version of their article under a licence inconsistent with the terms of our agreement – including the applicable embargo period – publication under the subscription model isn’t suitable for authors whose funders require no embargo.

If you have any questions about our publishing options, costs, Open Access requirements, or our legal forms, please contact ASJournals@springernature.com

An online order form for reprints of your paper is available at https://www.nature.com/reprints/author-reprints.html. All co-authors, authors' institutions and authors' funding agencies can order reprints using the form appropriate to their geographical region.

We welcome the submission of potential cover material (including a short caption of around 40 words) related to your manuscript; suggestions should be sent to Nature Microbiology as electronic files (the image should be 300 dpi at 210 x 297 mm in either TIFF or JPEG format). Please note that such pictures should be selected more for their aesthetic appeal than for their scientific content, and that colour images work better than black and white or grayscale images. Please do not try to design a cover with the Nature Microbiology logo etc., and please do not submit composites of images related to your work. I am sure you will understand that we cannot make any promise as to whether any of your suggestions might be selected for the cover of the journal.

You can now use a single sign-on for all your accounts, view the status of all your manuscript submissions and reviews, access usage statistics for your published articles and download a record of your refereeing activity for the Nature journals.

To assist our authors in disseminating their research to the broader community, our SharedIt initiative provides you with a unique shareable link that will allow anyone (with or without a subscription) to read the published article. Recipients of the link with a subscription will also be able to download and print the PDF.

As soon as your article is published, you will receive an automated email with your shareable link.

best wishes,

P.S. Click on the following link if you would like to recommend Nature Microbiology to your librarian http://www.nature.com/subscriptions/recommend.html#forms

** Visit the Springer Nature Editorial and Publishing website at www.springernature.com/editorial-and-publishing-jobs for more information about our career opportunities. If you have any questions please click here.**

Open Access This Peer Review File is licensed under a Creative Commons Attribution 4.0 International License, which permits use, sharing, adaptation, distribution and reproduction in any medium or format, as long as you give appropriate credit to the original author(s) and the source, provide a link to the Creative Commons license, and indicate if changes were made.
In cases where reviewers are anonymous, credit should be given to 'Anonymous Referee' and the source.
The images or other third party material in this Peer Review File are included in the article's Creative Commons license, unless indicated otherwise in a credit line to the material. If material is not included in the article's Creative Commons license and your intended use is not permitted by statutory regulation or exceeds the permitted use, you will need to obtain permission directly from the copyright holder.
To view a copy of this license, visit https://creativecommons.org/licenses/by/4.0/

We would like to thank the reviewers for their constructive critique of the work and hope we have been able to address all their comments in a positive manner below:

Reviewer Comments:

Reviewer #1 (Remarks to the Author):

Salman et al. have used a combination of growth assays, whole cell assays, biochemistry, structural biology, and bioinformatics to assemble a deeper understanding of the sulfatases in Akkermansia muciniphila. There is further clarity around the preferred substrates for some of these sulfatases, but transposon mutants showed little to no phenotypes when grown on mucin in comparison to the wild type. I have several questions/clarifications for the non-reproducibility of one of the substrates, growth curves, and the whole cell assays. Some of the interpretations may be going too far e.g. linking localisation to cross-feeding/competition, tn mutant lag phases. All these are detailed below and I hope they are helpful.

My interpretation is that the novel parts are 1) the 0121 structure and the activities against 6'S-Lewis A and Lewis X (although these activities are very weak?) 2) the CBM and 3) the inhibition data. However, these get a bit lost in the current narrative and you may want to elaborate on their analysis and conversation around them to bring out the parts of the work that is new information.

Narrative

• The abstract jumps around in terms of topic/focus and the main point the authors are trying to make/focus on is not clear e.g. what are the unique adaptations, it's not clear why the localisation of de-sulfation matters as a point here, "paints a contrasting picture" but how? It's a bit cryptic. Do you not want to focus on your novel parts?

RESPONSE: The abstract has been rewritten to be more focussed.

• Throughout the abstract and introduction a comparison is made between the way Akkermansia and btheta deploy sulfatases – but it is not clear what the differences are or why that really matters until the discussion e.g. line 104 the differences are not clear or why different is interesting.

RESPONSE: Lines 104-107 have been removed.

• The reader is drip-fed information about the Btheta enzymes throughout and then you don't say until half way through the discussion that the knockout of just one sulfatase in Btheta kills its growth on mucin whereas for Akkermansia you don't see any impact with sulfatase mutants (but don't mention the Tnseq findings?)

RESPONSE: This detail of a single sulfatase being required by B. theta. has been added

• I would drop the comparison with Btheta until the discussion, especially lines 104-107

RESPONSE: These lines have been removed.

• If you are going to focus on the comparison between Btheta and Akkermansia then the conservation of the different sulfatases across different strains/species needs to be assessed to strengthen why that comparison is important i.e. are these sulfatase present in many microbes and thus apply to many humans etc

RESPONSE: Much of the comparison to B. theta. has been removed but we still outline the work that link Bacteroides sulfatases to disease and mucin degradation using B. theta as the model.

Major issues

• Line 122 onwards- I think it's good that you have talked about the difference between batch preparations of cMOs, but reproducibility is a key issue here and it sounds like it would be difficult for other people to replicate your work.

RESPONSE: We are sure that the referee will appreciate that, owing to the inherent variation between individual animals, it is always challenging to perfectly replicate a particular extract. Furthermore, it is labourious to obtain and extract the colonic material from pig colons in the first place prior to generating the cMOs which is likely why most studies have utilised gastric mucin. That said, the general method for generating the cMOs employs proteinase K digestion and base catalysed elimination and, we think that the most likely source of variability is incomplete elimination of the cMOs from the protein component which, in this instance produced the gpcMOs with the particular characteristics we describe. Thus, in principle, we certainly agree that it should be possible to replicate the conditions to generate products that closely resemble ours.

In light of this, we would recommend that any isolated and prepared cMOs would need to be tested for its similarity against the structural data we provide, rather than relying on following an experimental procedure 'blind' as it were, and failing to check the structural features of the extract. We have provided the characteristics against which to check any attempted future isolate within Figure1 and Extended data figure 1. These will allow other researchers to benchmark their cMO preparations and we hope of value to the community.

• Why did the growth experiments not include the control of growths of both Akkermansia and Btheta on colonic mucin? Why go straight to oligosaccharides?

RESPONSE: Colonic mucin, as prepared from porcine distal colons, is insoluble and incompatible with spectrophotometric plate reader assays.

• Line 130 – how do you know that the trypsin and proteinase K have done anything? From the NMR? Wouldn't a glycopeptidase be better for this substrate?

RESPONSE: It is well published that trypsin cleaves colonic mucin into two large glycoproteins (https://doi.org/10.1016/S0021-9258(17)46696-8). Due to the insoluble nature of the colonic mucin (PCM) it can be observed as physically changing upon digestion. PCM incubated with water shows little to no soluble material upon analysis

by NMR whilst the NMR profile of the soluble fraction of Trypsin and proteinase K treated material shows an abundance of amino acid and carbohydrate signals with these materials supporting growth of Akkermansia. This data is in Figures 1 and Extended data figure 1.

It is likely a glycoprotease could potentially be better, but which microbiome glycoprotease would be key? This is an exciting project but outside the scope of the work. The use of trypsin and PK was facile and demonstrated that gpcMOs support enhanced growth compared to more intact digests of mucin glycoproteins produced by trypsin or PK.

• Did you remove the trypsin before growth? How do you know that it's not being used as a substrate? The details about the preparation of the trypsin and proteinase K treated substrates is missing from the materials and methods

RESPONSE: The proteases were not removed. *A. muc.* has not been shown to grow on proteins alone as nutrient source (The CMM base media is rich in peptides and proteins and does not support growth without an appropriate carbon source added). Furthermore, only 100 ug of the proteases was added in total (see methods below) making the amounts in the growth assay very low; >1000:1 for mucin to protease ratio. We would also point out that growth on trypsin treated PCM correlated with the concentration of the trypsin treated PCM. The concentration of trypsin was the same in both thus growth was independent of the trypsin of which the concentration was very low.

This information has been added to the methods:

Porcine colonic mucin (PCM) (400-500 mg) of was mixed with 30 ml of filtered milliQ water and incubated overnight at 37°C with shaking at 120 rpm. The suspension was centrifuged (30,000 x *g*, 15 minutes) and the supernatant was discarded. Pellet was re-suspended in 30 ml reaction buffer (50 mM Tris-HCl pH 8.0, 1 mM $CaCl_2$). Next, 100 µg of Proteinase K/Trypsin was added and incubated overnight at 37°C with shaking at 120 rpm. Digested material was centrifuged (30,000 x *g*, 15 minutes) and the pellet was discarded. The supernatant was heated at 95°C for 10 minutes for enzyme deactivation then dialysed extensively against water and finally filtered. The solution was then freeze-dried and final concentration was adjusted as per requirement. The PCM digests were kept at -20°C.

As a test we boiled trypsin and proteinase K and used this boiled material as a growth substrate at a final concentration of 5 mg/ml, see below. Although some growth is evident it is low and indicates the amount of inactive protease (µgs) in our assays did not support the growth observed.

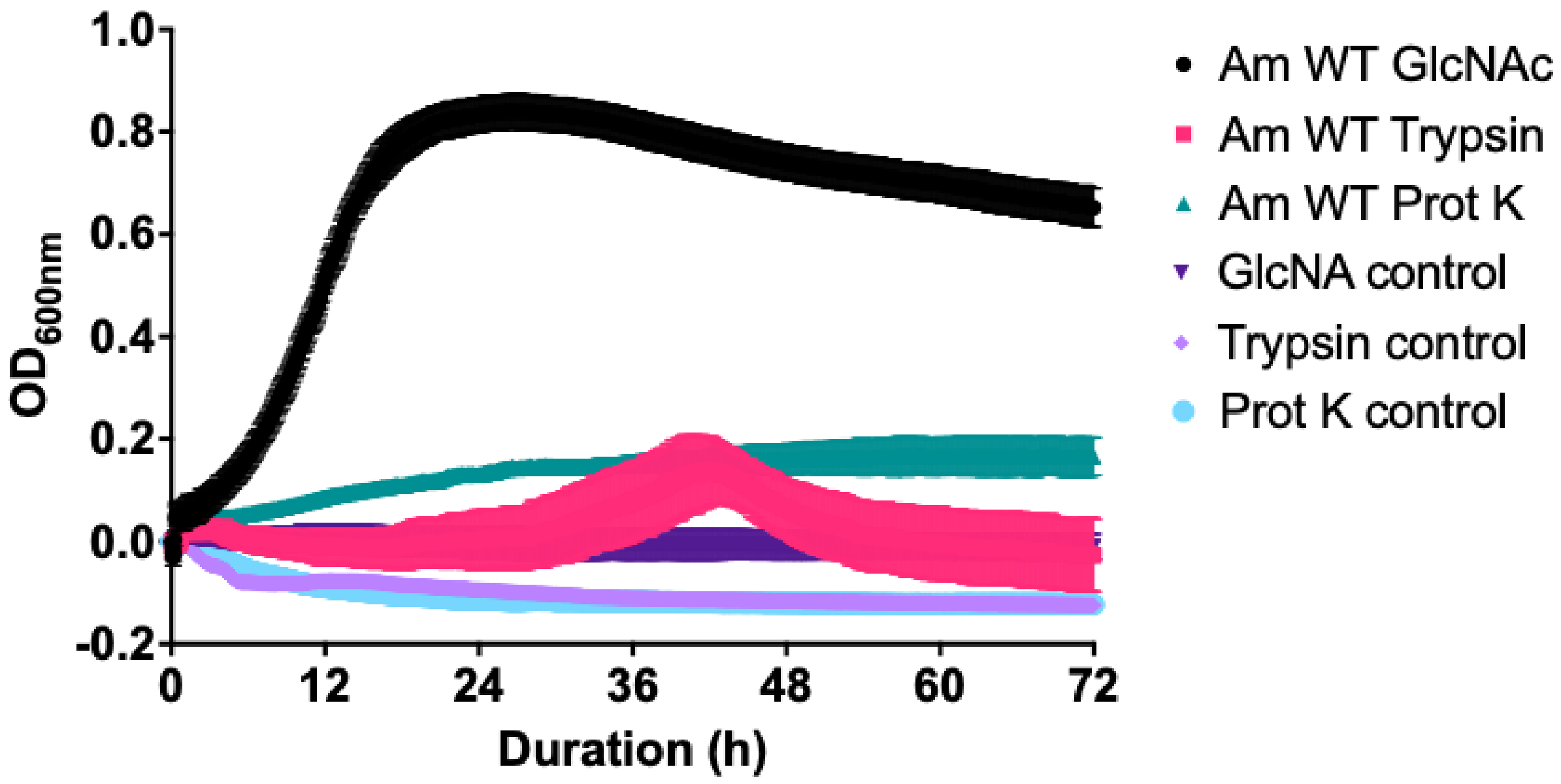

• Supplementary Figure 3

o where are the standards?

RESPONSE: The standards are not shown for clarity as in some instances they provide a much larger signal due to the amounts injected. We have added a panel showing the elution profiles of standards under the conditions used from the experimental run.

o For the no enzyme controls in some of them, the substrate peak is unclear e.g. 3'S-LeX and 3'S-LeA.

RESPONSE: The sulfated sugars either give no response or an extremely weak response using HPAEC coupled to electrochemical detection under our experimental conditions and we are exclusively looking at the production of the unsulfated sugar product.

o Personally, I'm not keen on the way the chromatograms have been presented with a time-shift as I think you may miss something. I appreciate there are pros and cons of different ways to present. Also, some are shifted and some not, so maybe pick one?

RESPONSE: These have been modified so as to not be shifted in this figure, now extended data figure 2 .

o What is the peak size expected for full de-sulfation? Why was this control not included?

RESPONSE: These experiments were qualitative to provide broad yes/no, active/not active answers, similar to the mass spectrometry data. We then paired this up with quantitative kinetic data and combined, this is far beyond the analysis previously attempted for these enzymes. We feel that no significant additional information would be garnered.

o Do the substrates not resolve well on the HPAEC runs? It's difficult to tell if small product peaks are due to incomplete

RESPONSE: Product peaks resolve well but sulfated sugars do not when using PAD as the detection method. The 3S-Lewis antigens are poor substrates for the S1_20 enzymes as they are degraded >100 times slower as shown by the kinetic data in figure 2a.

• Why were the 6'S-LewisA assays with 0121 not put on the HPAEC?

RESPONSE: This came down to substrate availability and HPAEC traces have now been included.

• Fig 2b – can you label the different substrates/products on the TLC? It is difficult to interpret.

RESPONSE: We have added lines between the standards of the breakdown products from the enzyme cocktail, we hope this helps. We have added HPAEC traces of this reaction as extended data figure 3.

• Fig 2b and line 173 – The control in the top TLC looks degraded already. It's difficult to see the 0121 activity.

RESPONSE: We understand the comment, but this is what the commercially acquired sample runs like in our solvent system. The higher bands are responsive to 0121 treatment and the result in lane 2 is a more homogenous sample where a desulfated trisaccharide is dominant. In lane 3 the addition of the fucosidase BT4136 and the galactosidase BT4243 cause no change and only with the enzyme cocktail are the monosaccharide constituents released.

• I think it would be good to have a figure for the mass spec gMO and cMO results so the reader can visually compare the differences. Do the cMOs have a much higher sulfation than the gMO in your work?

RESPONSE: Yes, cMOs and gpcMOs have a much higher level of sulfation than gMOs, consistent with the mucin from which they are generated. We have added extended data 1c which is a graphical representation of the major differences in supplementary dataset 2:

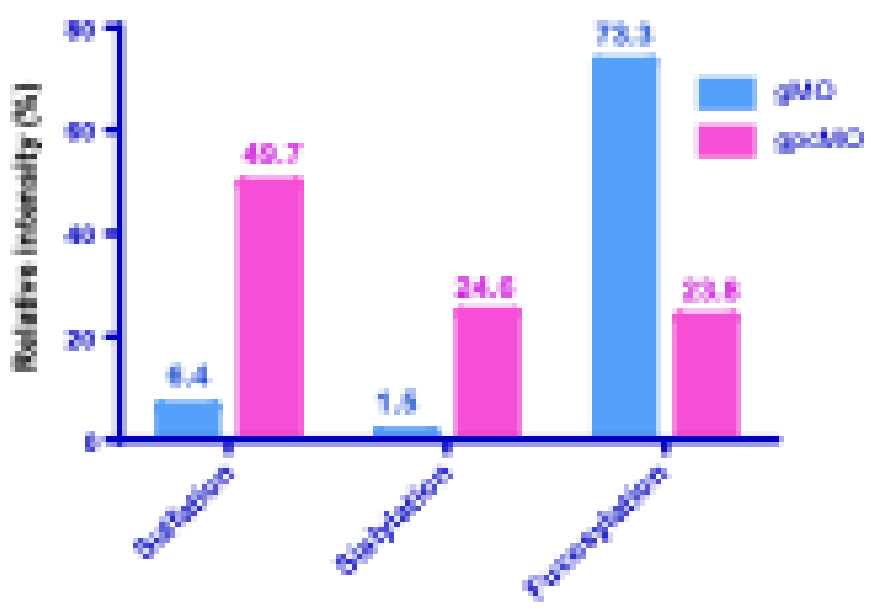

• For figure 2f

o How do you end up with more than what you started with?

RESPONSE: The bar charts represent relative abundances rather than absolute quantities. Just because the enzyme has not acted on that structure does not mean it has not altered the sample overall, and thus altered the relative abundance. The amounts measured for each individual glycan are also small, so relatively small changes can have a significant effect. Importantly, the complete absence of a bar (0%) reliably indicates enzymatic activity on the tested structure.

o Some details of the linkages are absent – is this because they are unknown?
RESPONSE: In short, yes. Some linkage details are absent because they cannot be determined unambiguously with the available data. This limitation arises because distinct glycan structures can share identical masses; for example, both 3-O-sulfated Gal and 6-O-sulfated Gal can occur at terminal position of lacto-N-biose I (type 1 LacNAc) or N-acetyllactosmine (type 2 LacNAc) structures in cMO. In such cases, MS^1/MS^2 analysis alone is insufficient to distinguish between these isomers due to lack of diagnostic fragmentation. Resolving these ambiguities often requires additional CAZyme digestions to enable unambiguous structural assignment. Without these complementary analyses, certain linkage positions cannot be confidently defined by mass spec alone.

o I think these panels can take a moment to interpret as the accompanying structure can either be a substrate or a product. Maybe both could be presented and you can say which structure the bar charts represent?
RESPONSE: All the sulfated structures are substrate for some of the sulfatases, or not at all for the others. The former product panels Column 1 and 2, row3, and column 3 row 2 show the desulfated product of the graph directly above them. For clarity we have now removed the product chromatograms as they are not needed to understand the activity of the enzyme and may confuse the reader.

o For the panels where there's no control and a product appears (left, third one down/centre, third one down/right, second one down) does the mass spec also show the substrate? And vice versa for the disappearing substrates – does the mass spec show a product?
RESPONSE: Yes, see above comment.

• You've made clear that kinetics is important for a deeper understanding of the biology but not compared and contrasted the results you present in Fig1a between the different enzymes and what these mean for the reader. There's a bit in the discussion, but needs to be brought forward in the text.
RESPONSE: We believe the discussion is the appropriate place for these comparisons within the limited word count of the manuscript.

• Whole cell assays –
o Why did you do the experiments with cells grown on both mucin and GlcNAc?

RESPONSE: Before we had carried out the proteomic expression data we postulated sulfatase activity would vary by substrate. The data with two substrates are more robust and support that sulfatase expression is constitutive for the sulfatases examined by this method.

o why do you start off at such a high % desulfated product (in the bar charts)?
RESPONSE: The method of labelling we use is simple and facile (incubation of sulfosugar and label in anhydrous methanol at 65C) but results in some desulfation. The level of which varies for each sulfo sugar, very little occurs for 6S-GlcNAc whilst 4S-GalNAc is more susceptible. The critical point is the ability to see a loss of substrate and increase in product in response to sulfatases, which the substrates allow.

o I was not sure what the control samples were until I looked in the methods, so you may want to put that in the main text or legend.
RESPONSE: The line 'Control samples were cells that had been heat-deactivated at 95℃ for 10 minutes.' Has been added to the legend.

o Why do the controls sometimes have high % values similar to a positive whole cell assay and why do they sometimes have low values like the T=0. E.g. Fig 3 and Supp fig. 4c red 6S-Gal compared 6S-GlcNAc green
RESPONSE: As stated above the labelling method can cause some desulfation of the substrate and this appears to vary with substrate.

o In Supp fig 4c – what does the "(GlcNAc)" mean at the top of each bar chart? Is that what they were grown on?
RESPONSE: This is indeed the substrate they were grown on, but we have removed this and made sure the information is in the legend to, hopefully, avoid confusion.

o Why do you think you have such a small change in the 3S-Gal compared to other substrates?
RESPONSE: This links very well to the kinetic data in figure 2a. The Amuc S1_20 enzymes are tenfold less active on 3S-Gal than 3S-LacNAc. Unfortunately, we did not have more of the latter substrate readily available.

o Why are you not getting complete desulfation for any substrate?
RESPONSE: The assays were run for 4 hours only and we do not know how much active sulfatase is present. We did not run the experiments longer as we were concerned that beyond 4 h one may start to get cell lysis. We know that there is no detectable cell lysis up to 4h when the cells are maintained in the growth media with no centrifugation.

o Line 219-220 – "The amuc1655::Tn mutant retained extracellular activity but the increased activity upon cell lysis was markedly reduced." I think this could be phrased better
RESPONSE: We have rewritten the sentence:

The *amuc1655::Tn* mutant show decreased activity from the CFE indicating Amuc1655$^{4S\text{-}Gal}$ is periplasmic. The remaining activity observed for whole cells suggests Amuc1755$^{4S\text{-}Gal}$ is located at the cell surface and is consistent with it possessing an SPII signal peptide allowing it to be membrane anchored **(Figure 3a)**.

o Line 228 – I'm not sure that the CFE retains activity – it looks comparable to the WC to me, unless I'm missing something?
RESPONSE: We agree the patterns look similar to the control and we have removed this statement: 'Interestingly, the cell free extracts of *amuc0121::Tn* mutant retain activity indicating the presence of a second, periplasmic, 6S-Gal sulfatase.'

o Line 233 – inhibit the expression of the gene or inhibit the actual enzyme?... ah ok, so I read further now…I understand why you talk about inhibition, but would this still apply if you wash the cells etc AND is the enzyme expressed comparably under GlcNAc growth conditions. Shouldn't you have grown the cells on mucin, then washed, incubated with GlcNAc, then washed, then done the assay?
RESPONSE: We could not reliably wash the cells as centrifugation and washing with PBS caused cell lysis between 30 and 50 % the majority of the time, and invalidated the approach. We showed this using the LIVE/DEAD™ BacLight™ bacterial viability kit (Table S2).

I think the WC data-inhibition data-growth Figs 4a-c are nice experiments, but they are explained in an order that's difficult to follow. I think I understand, but it's not clear what exactly the different growth curves are and 253-257 needs to be clearer, the last sentence seems tacked on at the end.
RESPONSE: These data demonstrate that Amuc1074 is important for desulfating 6S-GlcNAc in the periplasm. The amuc1074::Tn mutant shows no defect when GlcNAc is the main carbon source in the presence of 1mg/ml sPGM whilst it shows a significant defect when 6S-GlcNAc is the main carbon source demonstrating Amuc1033 is not sufficient to fully metabolise 6S-GlcNAc. This correlates with kinetic data on 6S-GlcNAc-B-MU that demonstrate Amuc1033 has a 50 fold lower kcat than Amuc1074, and expression data showing lower abundances of Amuc1033. This is also borne out in whole assay data where activity of Amuc1033, in the amuc1074::Tn background, is only evident at high 6S-GlcNAc, and not at low concentrations.

We have rewritten the paragraph as below:

The lower efficiency and expression **(Figure 1f and 4a)** of Amuc1033$^{6S\text{-}GlcNAc}$ are borne out in growth assays where 6S-GlcNAc is the main carbon source in the presence of 1 mg/ml sPGM. The *amuc1074::Tn* mutant, where Amuc1033$^{6S\text{-}GlcNAc}$ is the main 6S-GlcNAc periplasmic sulfatase, is unable to fully metabolise 6S-GlcNAc with a lower growth rate and maximum O.D. compared to wildtype **(Figure 4c)**. Whilst, no phenotype is observed on the GlcNAc control.

• The novel CBM – the structure is comparable to known CAZymes, so what does the equivalent region on the CBM to their active sites look like i.e. is it possible to tell

if the binding site of the CBM is likely to be in the same location as the active site of the CAZymes? Has this evolved to be a CBM?

Unfortunately, Foldseek, and PDBeFold return no convincing matches to any CAZymes, or proteins for that matter, in the PDB (see below). We picked CBM89 to show our mucin binding protein is not related to this carbohydrate binding thus a distinct evolutionary event. We have now made supplementary figure 7 comparing the top 5 closest unique matches to the structures in the PDB as predicted by PDBeFold; they are all very poor.

FoldSeek

Foldseek Search — GITHUB — SÖDING LAB — STEINEGGER LAB

Results for job: eUaRhesTON5GjmyiGsq-okYqu-jXS4EnhADIlg

ALL DATABASES — AFDB-PROTEOME (4) — AFDB-SWISSPROT (7) — AFDB50 (177) — CATH50 (4) — MGNIFY_ESM30 (946) — PDB100 (2)

PDB100 2 hits

SHOW TAXONOMY — GRAPHICAL — NUMERIC

Target	Description	Scientific Name	Prob.	Seq. Id.	E-Value	Position in query	Alignment
2iou-assembly1_H	Major Tropism Determinant P1 (Mtd-P1) Variant Compl...	Bordetella bronchiseptica RB50	0.11	13.2	1.73e+0	38–372	
7c7d-assembly2_B	Crystal structure of the catalytic unit of thermostable GH...	Streptomyces thermodiastaticus	0.04	11.3	3.98e+0	84–373	

PDBeFOLD

##	Scoring Q	P	Z	RMSD	N_{align}	N_g	$\%_{seq}$	Query $\%_{sse}$	Target (PDB entry) Match	$\%_{sse}$	N_{res}	×	Title
1	0.14	-0.0	5.3	3.76	204	27	7	54	7o9q:B	56	308	☐	CRYSTAL STRUCTURE OF THE AWP1 (ADHESIN-LIKE WALL PROTEIN 1) A-DOMAIN FROM CANDIDA GLABRATA
2	0.14	-0.0	5.3	3.80	203	27	7	54	7o9q:A	56	307	☐	CRYSTAL STRUCTURE OF THE AWP1 (ADHESIN-LIKE WALL PROTEIN 1) A-DOMAIN FROM CANDIDA GLABRATA
3	0.14	-0.0	6.3	3.46	191	20	8	51	7o9p:A	60	307	☐	CRYSTAL STRUCTURE OF THE AWP3B (ADHESIN-LIKE WALL PROTEIN 3B) A-DOMAIN FROM CANDIDA GLABRATA SHOWING A GADOLINIUM CLUSTER
4	0.13	-0.0	6.1	3.64	198	20	9	51	7o9o:A	58	328	☐	CRYSTAL STRUCTURE OF THE AWP3B (ADHESIN-LIKE WALL PROTEIN 3B) A-DOMAIN FROM CANDIDA GLABRATA
5	0.12	-0.0	3.2	4.03	256	30	8	63	2iou:G	45	508	☐	MAJOR TROPISM DETERMINANT P1 (MTD-P1) VARIANT COMPLEXED WITH BORDETELLA BROCHISEPTICA VIRULENCE FACTOR PERTACTIN EXTRACELLULAR DOMAIN (PRN-E).
6	0.12	-0.0	2.3	4.16	261	27	8	54	2iou:H	44	508	☐	MAJOR TROPISM DETERMINANT P1 (MTD-P1) VARIANT COMPLEXED WITH BORDETELLA BROCHISEPTICA VIRULENCE FACTOR PERTACTIN EXTRACELLULAR DOMAIN (PRN-E).
7	0.12	-0.0	4.1	3.90	224	27	7	60	7jvi:A	55	421	☐	CRYSTAL STRUCTURE OF A BETA-HELIX DOMAIN RETRIEVED FROM CAPYBARA GUT METAGENOME
8	0.12	-0.0	3.6	3.99	225	32	6	63	7jvi:B	56	421	☐	CRYSTAL STRUCTURE OF A BETA-HELIX DOMAIN RETRIEVED FROM CAPYBARA GUT METAGENOME
9	0.11	-0.0	2.7	4.43	230	29	6	51	7osh:A	46	392	☐	ABC TRANSPORTER COMPLEX NOSDFYL, R-DOMAIN 2
10	0.11	-0.0	2.8	4.54	217	22	9	46	5g5o:A	42	338	☐	STRUCTURE OF THE SNAKE ADENOVIRUS 1 HEXON-INTERLACING LH3 PROTEIN, NATIVE
11	0.11	-0.0	0.7	4.88	258	32	8	46	6bea:A	43	433	☐	CRYSTAL STRUCTURE OF THE AUTOTRANSPORTER UPAB FROM E. COLI STRAIN CFT073
12	0.11	-0.0	3.1	4.28	223	29	4	49	7osj:A	47	392	☐	ABC TRANSPORTER COMPLEX NOSDFYL, MEMBRANE ANCHOR
13	0.11	-0.0	5.6	3.84	187	23	7	43	1xg2:A	50	317	☐	CRYSTAL STRUCTURE OF THE COMPLEX BETWEEN PECTIN METHYLESTERASE AND ITS INHIBITOR PROTEIN
14	0.11	-0.0	2.3	4.15	202	25	8	43	5g5o:D	42	338	☐	STRUCTURE OF THE SNAKE ADENOVIRUS 1 HEXON-INTERLACING LH3 PROTEIN, NATIVE
15	0.11	-0.0	2.8	4.44	230	35	6	49	7qba:A	52	403	☐	CRYOEM STRUCTURE OF THE ABC TRANSPORTER NOSDFY COMPLEXED WITH NITROUS OXIDE REDUCTASE NOSZ
16	0.11	-0.0	2.5	4.43	229	31	7	54	7o14:A	49	402	☐	ABC TRANSPORTER NOSDFY, NUCLEOTIDE-FREE IN LIPID NANODISC, R-DOMAIN 1
17	0.11	-0.0	2.6	4.48	230	31	7	54	7o13:A	49	402	☐	ABC TRANSPORTER NOSDFY, NUCLEOTIDE-FREE IN LIPID NANODISC
18	0.11	-0.0	2.6	4.70	220	28	9	46	5g5o:B	42	345	☐	STRUCTURE OF THE SNAKE ADENOVIRUS 1 HEXON-INTERLACING LH3 PROTEIN, NATIVE
19	0.11	-0.0	3.2	4.40	209	36	8	46	5g5o:C	42	342	☐	STRUCTURE OF THE SNAKE ADENOVIRUS 1 HEXON-INTERLACING LH3 PROTEIN, NATIVE
20	0.11	-0.0	2.5	4.66	216	28	9	46	5g5o:F	42	338	☐	STRUCTURE OF THE SNAKE ADENOVIRUS 1 HEXON-INTERLACING LH3 PROTEIN, NATIVE

- Line 344 – the tn mutants also had increased lag phases on control monosaccharides, so the effect is not to do with mucin as a substrate or the missing sulfatase

RESPONSE: This rather shows the importance of mucin as a substrate providing some essential nutrient beyond glycans. There are also two sets of GlcNAc growths

with only one showing some mild effects which for all but two mutants disappear on mucin substrates.

• Lines 365 – what does the Tnseq Davey paper suggest about the importance of GH16s/sulfatases/other CAZymes? Is there any evidence of this cross-feeding? Why would this benefit Amuc?
RESPONSE: The Davey et al. paper did not look at inter species cross-feeding.

The Davey et al. study did use a droplet-based Tn-seq approach to distinguish genes required for growth in isolation (single *A. muciniphila* Tn mutant cells in droplets) versus in a pooled *A. muciniphila* Tn mutant community. While most GH mutants showed no difference between these conditions, mutants in sulfatases, fucosidases, and sialidases displayed a strong growth defect when grown in isolation but not when grown in bulk (Extended Data Fig. 3e of Davey et al.). This pattern is consistent with cross-feeding, enzymes that cleave sulfate, fucose, or sialic acid from mucin glycans release products that can be shared among neighbouring cells, or allow neighbouring cells to access mucin structures. In bulk culture, mutants lacking these enzymes can scavenge products released by their neighbours, or access modified mucin structures that enzymes have acted on. This might benefit *A. muciniphila* by enabling a division of labour within the population.

Our point in the discussion was that the literature states GH16s are at the cell surface and produce cMOs, both in *A. muciniphila* and Bacteroides species. A. muc cannot use these so where do they go? This is what has been briefly discussed.

Minor issues
• Line 48 – only one species of Bacteroides has been characterised?
RESPONSE: The abstract has been rewritten.

• Line 89 – include reference
RESPONSE: We have added appropriate references.

• Line 97-99 – at this point the reader will not understand what is meant by processed and only the wild type growth curves revealed this, not the mutants?
RESPONSE: We have removed the term processed from this sentence. Wildtype and mutants growth curves all grow better on gpcMOs revealing the same trend but indeed the wildtype is sufficient to show this.

• Line 111-113 – should this reference Figure 1 too?
RESPONSE: Yes, we have added the callout.

• Figure 1 and Supp fig 1 – it would be useful to have all growth curves on the same scale to comparison
RESPONSE: We have adjusted all the mucin substrate growth curves to the same scale.

• Lines ~115 – Does Amuc also grow on GAGs? Probably should be mentioned in this contrast with the Btheta mutant
RESPONSE: No, Amuc does not grow on intact GAGs or the breakdown product of recombinantly expressed B. theta. GAG degrading enzymes (Supp figure 1).

• Line 117 – PCM is not defined
RESPONSE: The acronym has now been defined in the sentence.

• What do you think about the biphasic growth for the Btheta mutant on gMOs and the btheta wild type on CMO
RESPONSE: We agree this is an interesting observation. We have two potential theories bearing in mind the oligos are a complex mixture and 15 PULs in BT have been implicated in mucin metabolism.

As the gMOs and cMOs are a complex mixture of glycans it could be that some structures are transported into the periplasm more readily than others, and cause the upregulation of certain PUL components then the slower transported ones upregulate a different set of PUL components.

It could be that some structures are broken down less efficiently than others and these are only used when the simpler structures have been depleted, or a combination of these factors.

• I find following the different substrates and what treatments they have had and which growths curves match the text quite difficult in the first section. Would it be worth labelling the figures in line with the text acronyms?
RESPONSE: We have labelled the figures with the text acronyms and hope this helps the readability.

• Fig 2 - There's a mistake in the legend saying that 109s target beta-linkages
RESPONSE: GH109 can target alpha and beta linkages, as can a number of GHs that use the NAD dependent method of hydrolysis https://www.cazy.org/GH109_characterized.html. We are in fact using the very same GH109 in a linked assay against alpha and beta linked GalNac to methylumbelliferone to measure sulfatase activity (see below). We would point out the chemistry of these enzymes begins by abstraction of the O3 proton.

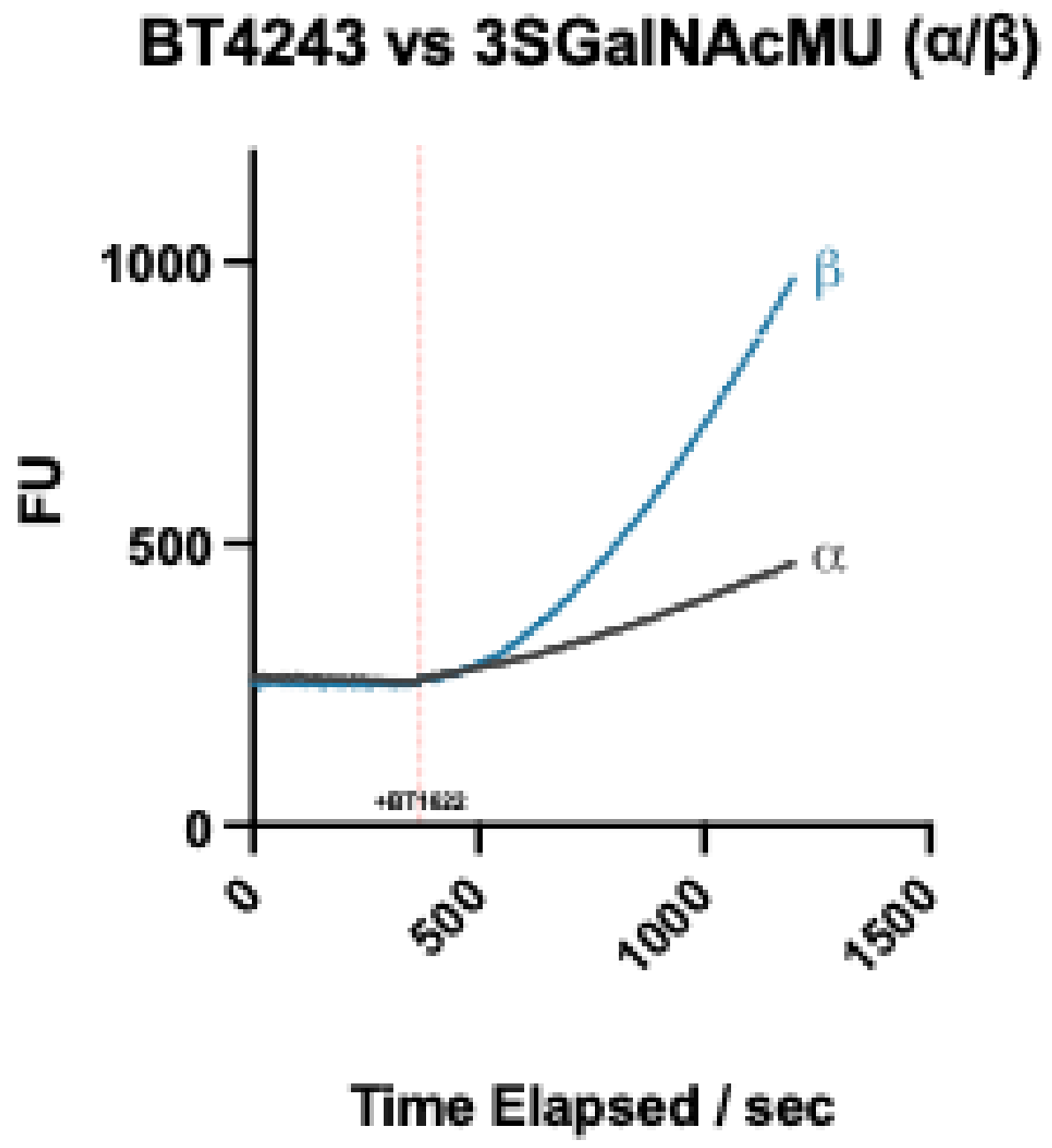

• For the non-NMR expert – which parts of the profiles correspond to glycopeptide? How can you the difference between glycopeptide and glycan+peptides? The mass spec only shows the released glycans?
RESPONSE: The quick answer is none. The profiles show signals for amino acids and glycans. A. muc. does not grow on just free glycans, or just proteins extracted from the mucin. We then confirmed glycoproteins using a glycoprotein stained gel (fig 1c), and the mass spec data in (fig 1d) which shows of fragments of glycopeptides from the fragmentation. The data are very much to be interpreted as a whole.

• 0121 structure – It would be good to comment on how the accommodation of 6'S-LewisX compared to A might be?
RESPONSE: 6'S-Lewis X will be accommodated in a similar fashion to 6'S-LewisA. The GlcNAc sugar is essentially flipped with the galactose and fucose adopting the same positions. We have added supplemental figure 3 to show this, and in the text stated the line in yellow:

'This allows the Fuc to sit above a hydrophobic patch created by the conserved residues Val77, Ile99, and Tyr361[32], whilst the *O*6 of *N*-acetyl-D-glucosamine (GlcNAc) interacts with the conserved residue Arg170 that is part of the Gal recognition triad within roughly half of S1_15 members[32]; a similar binding mode is likely to be adopted by 6'S-LewisX **(Supplementary figure 3)**.'

• Supp figure 3 – Gal is a circle, not a curved-corners square
This has been corrected.

• Line 149-151 I am not sure of the context of this last sentence. You have stated that a sulfatase is upregulated under certain conditions and then said that this

suggests something about protease activity? I think you need to spell this out better for the reader

RESPONSE: We have attempted to clarify the paragraph by indicating that colonic mucins largely do not alter sulfatase expression, compared to gastric, but our data do show that they are required for upregulation of the glycoprotease Amuc_0627. This means either sulfation, glycopetides, or both are needed to upregulate this gene. See below:

When comparing gpcMOs to sPGM samples only three sulfatases (Amuc0491, Amuc1655 and Amuc1182) are more upregulated (**Figure 1f and g**). The gpcMO samples did show that the mucin glycoprotease, Amuc_0627, was highly upregulated **(Figure 1g).** Overall, the different gastric and colonic mucin substrates did not lead to large differences in sulfatase expression but suggest that Amuc_0627 expression requires colonic glycoproteins but not gastric **(Supplemental figure 1c and Supplementary dataset 3).**

• Line 159 – You say you worked out pH, but then you don't comment on that? What was optimal and what do you think about this from the point of view of the biology?

RESPONSE: As standard practice we determine the pH optimum of the enzymes we work on. It can be important for determining activity if the pH optimum is particularly narrow and not centred around neutral. The data here show all the pH optima centre around pH 6, close to the pH one may expect in parts of the colon.

• Line 182 – You may want to explain the impact of method-of-production on kinetics better for the reader – what is FGly?

RESPONSE: We have amended the sentence to state formylglycine is the catalytic nucleophile, see below:

'No kinetic measurement could be accurately made for the Amuc0121$^{6S\text{-}Gal/GalNAc}$ likely to be due to lower installation of the catalytic formylglycine nucleophile by *E. coli* during recombinant expression, reported previously for the S1_15 subfamily[32].'

• Line 197 – the final sentence, similar to Btheta enzymes in what way? As in the subfamilies are acting on the same types of substrates?

RESPONSE: We have amended the sentence to read:

These results indicate that *A. muciniphila* encodes sulfatases to remove the main sulfated linkages found in complex cMOs and uses the same S1 subfamilies as reported for *B. theta.*[9].

• In the Figure 3 model – what is the difference between "weak sulfatase activity" and "inefficient or slow sulfatase activity"

RESPONSE: We have removed these terms and replaced them with the term low sulfatase activity and do not try to differentiate between weak and inefficient.

• Fig 4c – the growth curves need to be put on the same scale

RESPONSE: It would be more difficult to observe the phenotype and the X-axis is clearly labelled. We believe the data to be clearer as presented.

• Fig 4e – what are the different colours?

RESPONSE: We have added the line below to the legend:
'The differing loop colours indicate the five different predictions of the loop confirmation from the AF2 prediction. '

• Fig 5d – label different coloured parts of the structure. Bacon, ig-like, B-helix domain etc
RESPONSE: The domains have been named as requested.

• Line 354-356 – say which piece of data tells you this
RESPONSE: We have adjusted this line:

'Our growth data validate this finding but indicate that *A. muciniphila* requires glycopeptides of a specific size and/or motif to support strong growth on colonic mucin.'

• More detail needed in many of the legends
RESPONSE: We have added details to the legends where they have been specified.

• Line 370-1 - info for the intro?
RESPONSE: This information on colonic mucin sulfation has been moved to the introduction.

• Two paragraphs starting Line 388 – Aren't these the insights that you have solid data for, so they should be focussed on more?
RESPONSE: We have tried to balance the discussion between the discussing the data and the context of which it sits in the literature.

Reviewer #2 (Remarks to the Author):

In this work, Dey and co-workers study the role of sulfatases from Akkermansia muciniphila, a mucin degrader and a major member of the human colonic microbiota. Using wild-type and sulfatase transposon mutants, the authors demonstrate that processed glycoproteins fragments, rather than colonic mucin glycoproteins, are the preferred substrates of the bacteria. The authors recombinantly expressed 10 sulfatases and detected activities for 8 of them, in line with recent reported study (doi:10.1038/s41564-024-01911-7), but using fluorescently labelled substrates as well as model and mucin derived glycans. The authors provide crystal structures of five sulfatases, including Amuc0121, Amuc0451, Amuc0953, Amuc1074 and Amuc1755, in the absence and presence of ligands. It is worth noting that Amuc0953 contains a novel CBM family that binds colonic mucin, which seems to be restricted to the genus Akkermansia. Based on cellular localization assays, the authors proposed a processing model for the reported sulfatases. The manuscript is well written, the interpretation of the experimental data supports the proposed model and markedly advances the field.

Some brief suggestion for improvement:

Please, incorporate images showing the overall fold of each Amuc0121, Amuc0451,

Amuc0953, Amuc1074 and Amuc1755 structures in the corresponding main text figures for clarity.
RESPONSE: Structures of Amuc0121, Amuc1074, and Amuc1755 have been added to the figures. The overall fold structures of Amuc0451 and Amuc0953 were already present.

Congratulations to the authors on this very nice manuscript.

RESPONSE: We thank the reviewer for their kind assessment and are happy they approve of the work.

Reviewer #3 (Remarks to the Author):

This manuscript by Dey, Salman et al presents a broad-scale survey of Akkermansia muciniphilia sulfatases, and their role in supporting microbial utilization of gut mucin substrates. Akkermansia muciniphilia in the gut is inversely correlated with health outcomes, so understanding the factors that support colonization is important in the context of microbiome and wider systemic health.

Here, the authors have conducted growth profiling of Akkermansia on different mucin preparations, finding that glycopeptide contexts are required for microbial utilization of mucins. There is also profiling of (limited) changes to sulfatase expression during growth on mucin, followed by fairly extensive structure/function characterization of different classes of mucins expressed by Akkermansia. A new CBM family is claimed for one particular sulfatase N-terminal domain (Amuc_0953). Finally, the effect of different Akkermansia transposon mutants on sulfated mucin utilization is explored.

Whilst the topic in this paper is of broad importance, my enthusiasm is lessened somewhat by (in my opinion) slightly incomplete claims in some areas. In particular, one of the important findings seems to be that the nature of colonic mucin substrates matters for utilization by Akkermansia, with peptidic mucins preferred. However, this observation not explored in much depth beyond growth curves.
RESPONSE: We have explored the mucin substrates and growth preferences within the scope of the manuscript, see comments below.

A secondary comment is that the overall presentation of results that is hard to follow. As a caveat, I have expertise in carbohydrate enzyme mechanisms but not microbiome, so I may lack some contextual knowledge in the field, but presumably the manuscript does not want to be so specialized as to be inaccessible to outsiders.
RESPONSE: We have responded to all comments on the figures as suggested by the reviewer, but without specific guidance it is hard to rectify specific oversights.

Some scientific comments

1 – It is claimed that Akkermansia requires glycoprotein forms of mucin for proper growth with porcine colonic mucins (PCMs), but not porcine gastric mucins (PGMs).

As I understand it, this stems from a serendipitous observation that a second batch of colonic mucin oligosaccharides (cMOs) was enriched in protein/peptides. This led the authors to try protease digests of cMO before bacterial growth, which subsequently supported Akkermansia growth to a limited extent. This observation seems important but its context and implications are not discussed in much detail.

My inference from the rest of the text is that more sulfated cMOs (vs PGMs) require breakdown to peptidic substrates to support sulfatase function before carbohydrates can be utilized by glycosidases. However, there don't seem to be further experiments to probe this. Functional studies on recombinant enzymes use model fluorescent mono/oligosaccharide substrates (Fig 2a,b) or whole cMOs (2f), but not cMOs in glycopeptide contexts, hence this observation of mucin context ends up being sidelined.

RESPONSE: We agree this is an interesting area and the data we have produced lays the foundations for further investigation. We would highlight the material we use here is extracted from porcine colons and is not off the shelf so takes considerable effort to prepare. Furthermore, we went to extensive efforts to characterise our mucin preparations through NMR, mass spectrometry, glycoprotein gel staining, and microbial growth experiments to reach the conclusions we have. To follow up this work would need strategies for characterising a variety of glycoproteases on PCM, and/or modifying the chemical methods of glycan liberation to reproduce the gpcMOs so they could be fractionated and characterised. This is an exciting project/paper in itself and beyond the scope of this manuscript.

2 – Discovery of a new CBM family with Amuc0953. Again this seems important, but the evidence presented is a single pulldown carried out with PCM, with BSA as a control. I see negative PGM interaction is proposed in the Supplmental Information using TLC (Fig S12). However, TLC may not reveal interactions - PGM would also be a useful control in the pulldown experiment to gauge CBM interaction with more/less sulfated mucins. Similarly, differences in interaction between PCM and trypsin/proteinaseK PCM should be looked at, and if possible, also interactions with cMOs.
RESPONSE: The TLC analysis was only to show there was no enzymatic activity from the domain against the glycans tested.

We have added affinity gel electrophoresis analysis using gastric mucin oligosaccharides (gMOs) and the last of our glycoprotein colonic mucin oligosaccharides (gpcMOs). A marked shift is observed only with the gpcMOs relative to BSA as compared to the no glycan control gel. These data confirm an interaction with gpcMOs and not gMOs.

3 – Minimal differences in growth between Tn mutants and wt Akkermansia on a variety of mucin substrates certainly seems interesting. However, this section seems to lack some context e.g. growth lag observed with amuc0490-491::tn which is attributed to amuc0490. What is amuc0490? It is not mentioned elsewhere in the

paper. Presumably not a sulfatase?

RESPONSE: amuc0490 is an inner membrane helical protein. We have data showing that it displays a phenotype on GlcNAc and soluble PGM, but its exact role is unclear and beyond the scope of the manuscript.

More broadly, what is the link that drives differential growth on sPGM and gpcMOs in this case? Are there issues to do with transport of different mucin sources? Are there cooperative effects for growth that would only become apparent upon mutating more sulfatases? This should at least be discussed more in the relevant results section, or in the final discussion.

RESPONSE: There seems to be some confusion on this. A. muc. grows very well on sPGM and gpcMOs. The major difference is gMOs vs. cMOs, where the former provides weaker growth and the latter no growth. Thus, the conclusion is that processed glycoprotein/peptide forms of mucin are needed for efficient growth on gastric mucin but are essential for colonic mucin. As stated in the manuscript line 134-139

' These data reveal that *A. muc* targets glycopeptide/protein forms of mucin and these are needed for robust growth on gastric mucins but absolutely required for growth on colonic mucins. By contrast, *B. theta* could grow on gMOs, cMOs, and gpcMOs but grew more poorly on gpcMOs **(Figure 1a)**. This indicates that *A. muc* targets specific glycopeptides of a particular size and/or motif that were serendipitously captured in our gpcMO preparation, and these cannot be fully recapitulated by trypsin or proteinase K treatment.'

On a minor note, the overall presentation, annotation and interpretation of figures seems poor in places. Some examples (I have not checked exhaustively).

1 – Fig 2c – in the text, this data is supposed to support the fact that Amuc0121 has lower affinity for 6SLewisA vs 6SGal (L174). But the data shows a thermal shift assay. Whilst this experiment can correlate with ligand affinity, it is fundamentally measuring something else, so the interpretation is not sound.

RESPONSE: Thermal shift assays measure a change in melting point of protein which is indicative of an interaction. The stronger the shift the stronger the interaction, and in our assay only the substrate is changing, not the protein i.e. both substrates are interacting with the same active site. The methodology and its interpretation is well-established and we believe our interpretation is sound.

2 – Fig 4a – Inner Michaelis-Menten plot is so small as to be unreadable. What is this dotted line representing?

RESPONSE: The plot has been expanded and the figure adjusted to accommodate it.

3 – Fig 4e – lots of different coloured loops going on here. Needs annotation.

RESPONSE: The different coloured loops have been detailed in the legend, they belong to 5 different AF2 model predictions and demonstrate the relative amount of flexibility in terms of 1033.

4 – Fig 6b – again, annotation poor for quite a complex figure. Would be better if figure caption text was instead used to annotate figure directly.
RESPONSE: We have added additional annotation to the panel b.

5 - Supp figure 12 - typo on top line
RESPONSE: The incorrect spelling of binding has been corrected.

Reviewer #4 (Remarks to the Author):

This study by Dey et al. presents a "deep dive" into the function of the family of 10 sulphatases in mucin utlisation by the human gut bacterium Akkermansia muciniphila. The authors present bacterial growth and targeted gene knock-out data, enzymology, and structural biology to provide a rather full picture of where and how these enzymes act on specific mucin substructures. The work is timely and valuable, following closely on a recent study in this journal (ref. 34, 2025) on the topic of mucin degradation by A. muciniphila, in which the same sulphatases were identified and characterized preliminarily. The present study takes the analysis significantly further. This notwithstanding, many journals now rightly have "scoop protection" policies that mitigate concerns of "novelty", and I would suggest that that same courtesy should be applied here.

The study comprises a large, diverse, and high quality set of data, the conclusions from which are clearly described and well-supported. The following comments should be addressed to improve further the study and its presentation:

Major comments:
1. Lines 316-335 and related text elsewhere: My only major concern with this manuscript is the claim that the distinct N-terminal domain of Amuc0953 constitutes a new carbohydrate binding module family. The pull-down data is certainly indicative of mucin binding, but this does not unambiguously reveal whether the interactions are mediated by protein-carbohydrate interactions, protein-protein interactions, or a combination of both. The specific residues in Amuc0953 responsible for binding and the specific epitope(s) on mucin are unresolved. Crystallographic complexes and interaction studies with mucin fragments of defined glycan structure would go along way to resolving this question. Regardless, however, the observation that this domain is found essentially exclusively in Akkermansia indicates that creation of a new CAZy family would constitute a small family with little sequence diversity and limited predictive power across microbial taxa, which I believe is the raison d'etre of the CAZy classification and bioinformatic analysis through modular dissection.

The revelation of this new structure is clearly one of the key novel and exciting aspects of the manuscript. I would only suggest that the authors refrain from calling this a new CBM, and refer it is simply as a "novel mucin binding domain (or module)", e.g. in the title of Fig. 6, in line 316, etc.
RESPONSE: We agree with this conclusion and have altered the text accordingly to

only describe the domain as a novel mucin binding domain. We have also expanded our analysis by including AGE with gMOs and gpCMOs (see comment above to reviewer 3).

It is clear that this new domain is not similar to CBM89, based on the analysis provided in Fig. S12 (n.b. check spelling of legend). However, comparison with this particular beta-helix domain family may not be the most appropriate. What are the top hits from a structure-based search of the entire Protein Data Bank? The authors should confirm that their analysis was not unduly biased by only considering that this might be an evolutionary derivative of a CBM lineage.
Unfortunately, Foldseek, and PDBeFold return no convincing matches to any CAZymes, or proteins, in the PDB (see above in response to reviewer 3). We picked CBM89 to show our mucin binding protein is not related to this carbohydrate binding thus a distinct evolutionary event to CBM89. We also acknowledge that we do not know what lineage the domain descends from and have refrained from calling it a CBM as the reviewer correctly advised. We have also included supplementary figure 7, where we compare the closest five matches from the PDB. All are poor, but CBM89 seems to score slightly better when considering RMSD and the number of residues aligned.

2. Line 85: Bakshani et al. (ref. 34) identified 12 potential sulphatases in A. muciniphila, but here it is stated that there are 10. Why the discrepancy? This should be addressed explicitly in the text
RESPONSE: Bakshani et al claim to have identified 11 sulfatases in their published paper, but this was 12 in their initial preprint. In their preprint they had incorrectly identified an unrelated protein, Amuc_1290, as a sulfatase, but corrected this prior to publication in Nature Microbiology. They persisted with the claim that Amuc_0864 is an S1_26 sulfatase, but the protein sequence does not have the C/S-X-A/P-R consensus sequence for formylglycine incorporation, thus cannot be an S1 sulfatase, nor does it have the calcium binding site, and is not contained in the SulfAtlas database. Thus, 10 S1 sulfatases (as we state) is correct, rather than 12 or 11. We do not feel we have enough space in the text to correct other papers, but we hope our correct analysis will perpetuate in the literature. Those authors could correct their article with an erratum/corrigendum.

Minor comments:
1. Line 40 and lines 70-84: Given the mixed biological effects of A. muciniphila on hosts, should the first sentence o f the abstract be more balanced, e.g. "...and has been implicated in health."? Should the key study by co-author Martens (Cell. 2016 Nov 17;167(5):1339-1353.e21. doi: 10.1016/j.cell.2016.10.043.) be cited in the context of lines 80-84?
RESPONSE: We have added the reference.

2. Please use either "A. muciniphila" or "A. muc." consistently throughout. Like "A.", "muc." is also an abbreviation, so requires a period. Ditto for "theta."

RESPONSE: *A. muc* has been replaced with *A. muciniphila*, and *B. theta* with *B. theta*. throughout.

3. Lines 145 and 149: Replace "significantly" with "highly". I believe the authors are referring to the x-axis in Fig. 1g, not the y-axis. Throughout, the word "significant" should be reserved for use in the statistical sense.
RESPONSE: We have changed to the use of 'highly' in these lines, and yes we are referring to the x-axis.

4. Lines 214-216: This reads like a sentence fragment. Please revise.
RESPONSE: The sentence has been revised as follows:

'These data indicate that Amuc0953$^{3S\text{-}LacNAc/LNB}$ is responsible for the extracellular activity and Amuc0451$^{3S\text{-}LacNAc/LNB}$ for intracellular activity. Amuc0953$^{3S\text{-}LacNAc/LNB}$ possesses an SPI signal peptide which drives localisation to the periplasm thus an additional mechanism must translocate and anchor the protein to the extracellular membrane.'

5. Lines 237-239 and 370-378: Is the sulphation of 6S-GlcNAc in the periplasm biologically meaningful, or just a quirk of evolution? That is, is there a reason why the corresponding sulphatase is not delivered to the cell exterior? Do orthologues in other species or strains contain signal peptides for extracellular targeting?
RESPONSE: We have identified two orthologues for most activities with a copy outside and inside the cell for galactose desulfation. The periplasmic location of both 6S-GlcNAc sulfatases could be a way to make sure that A. muc. prioritises GlcNAc for itself by not desulfating these sugars at the cell surface for organisms that lack sulfatases to access, such as R. torques and R. gnavus. The S1_20 orthologue, BT1636, in B. theta. has been shown experimentally to be at the cell surface and has an SPI signal peptide. How the sulfatase gets to the cell surface is unknown.

Predicting protein localisation in gram negative bacteria based solely on SPI or SPII signal peptides is difficult. SPI proteins should be periplasmic, but have been seen in extracellular locations. SPII signal peptides illustrate membrane attachment, this could be in the periplasm or on the bacterial cell surface. In the Bakshani et al paper they have solely relied on signalP predictions and have incorrectly predicted the location of at least 4 of the 7 sulfatases they have illustrated in figure 6 of their paper. Thus, experimental validation of enzyme location is important in understanding the biology of the organism; it allows one to detail what parts of metabolism happen where.

6. Line 267: "loss of productive energy" Please rephrase in a form more typical for physical chemistry.
RESPONSE: We were a little unclear as to how to rephrase this but we hope the following sentence is okay:

'This leads to a substantially reduced k_{cat} but not K_M, indicating reduced transition state stabilisation.'

7. Lines 293-294: The use of the descriptor "BACON" should be deprecated. I

understand that there is no experimental evidence to support the statement that these domains are carbohydrate binding. If otherwise, please provide references. The acronym can be suitably replaced with a corresponding InterPro or Pfam identifier with no detriment to the text. The authors may also wish to include a description of the overall fold of this domain, as done for other domains described in the surrounding text.
RESPONSE: We have used the term Ig-like PA14 for the abutted C-term domain as described by interpro scan.

8. Line 301: Revise to "translocation of"
RESPONSE: This has been revised

9. Line 305: Spell out "Histidine". "His" is easily mistaken for the pronoun at the start of the sentence.
RESPONSE: His has been changed to Histidine at the start of this sentence.

10. Line 363: "the Bacteroides organism" is redundant in this sentence.
RESPONSE: We have removed this redundant text.

11. Line 394: Perhaps it is worth stating explicitly that salivary mucins would in fact be expected to transit the GI tract as a normal consequence of eating. As such, all members of the HGM/HCM might be anticipated to encounter this substrate.
RESPONSE: We have modified the relevant section to be:

'The apparent rare specificity for Gal over GalNAc exhibited by the S1_16 enzymes suggests that 4S-Gal, detected in salivary mucins[48] which transit the colon as part of the digestive process, is a motif targeted by *A. muc*, and the specificity of the *A. muc* sulfatases may give it a competitive edge on this substrate.'

12. Line 397-399, perhaps elsewhere: Is it possible that this protein requires 6S-GlcNAc to be presented on a polypeptide for maximum activity? I maybe missing something here, but in general (e.g. Fig. 3) substrate specificity seems to be considered only for free mono/oligosaccharides.
RESPONSE: This is an excellent suggestion and we have amended the sentence:

'The S1_11 enzymes in *A. muc* that target 6S-GlcNAc have vastly different activities driven by differences in the flexibility of a key *N*-acetyl loop. Why *A. muc* has an apparent low efficiency 6S-GlcNAc sulfatase is unclear, but it is possible this enzyme targets a larger 6S-GlcNAc containing glycan, or glycopeptide.'

13. Line 401: "Gram" should be capitalised as it is a surname.
RESPONSE: Gram has been capitalised

14. In the legends for Fig. 5d and Fig. 6a, please clarify which structure(s) is experimental and which is modeled (and if so, how).
RESPONSE: In the legend of d. we state Amuc1074 is a crystal structures and the PDB code is given for BT3177. We have now added the resolution to which it was

solved to reduce the ambiguity.
End of comments.

The reviewers' comments, with the exception of those from reviewer 1 which, as we will explain below are erroneous, are all supportive of the work and require only typographical corrections which we are happy to provide. This also covers reviewer 2's comment concerning TSA. We were, therefore, surprised by the decision to reject our revised manuscript. We ask that you consider our responses to reviewer 1's comments (outlined below), since the criticisms are largely unfounded and, indeed, incorrect in places.

May we suggest that an equitable course of action could be to enlist an additional, 5th, reviewer to read the revised manuscript and the responses that we provide below to reviewer 1?

Reviewer Expertise:

Referee #1: Microbial mucin degradation
Referee #2: Glycobiology, Enzymology
Referee #3: Glycobiology, Structural Biology
Referee #4: Biochemistry, Glycobiology

Reviewers Comments:

Reviewer #1 (Remarks to the Author):

Thank you for your detailed responses. Most of the data you present is of a high standard, but unfortunately, I am not convinced about some of the data, some of the interpretations, or the answers provided on some of the issues raised first time.

My re-queries are:
1) The TLC in Fig2b – this is the core evidence of 6'S-LewisA/X activity, which is a really important element to the novelty. In my opinion the assays and TLCs need re-running. The two furthest left spots for the top TLC (control and +0121) - where have the blue and pink spots gone in the +0121 sample? How does this weird result relate to your response about the substrate being commercially acquired? They look like different substrates on that TLC. The bottom TLC is also not a clean result.

RESPONSE: The activity of Amuc0121 on 6S-Lewis antigens is supported by TLC, HPAEC and TSA. In addition, we do not rely solely on this TLC result; the substrate used in these assays was also co-crystallised with the protein revealing the atomic details of the protein-substrate interaction. The comment 'they look like different substrates' is curious as when an enzyme acts on a substrate it produces a product and this must look different from the start material i.e. spots for substrate disappear and new spots relating to product appear. The pink and blue spots are the substrate.

Focusing more on the 6S-LewisA; it is true the control has three spots, two of which (the blue and pink) respond to Amuc0121 to produce a single spot, meaning that they produce the same product upon removal of the *O*6 linked sulfate group. The 6S-LewisA substrate is unresponsive to the fucosidase BT4136 and galactosidase BT4241 which is consistent with them being active on LewisA but not 6S-LewisA. Upon addition of Amuc0121, and both BT4136 and BT4241, the products of

galactose, fucose, and N-acetylglucosamine are produced, consistent with the removal of *O*6 sulfation thereby allowing access to the LewisA substrate. The data are corroborated with HPAEC analysis.

The 6S-LewisX TLC is a cleaner result and clearly shows the same trend as the 6S-LewisA data; there is a definitive shift upon the addition of Amuc0121, and Amuc0121 allows access of the galactosidase and fucosidase to degrade the substrate. Again, these data are backed up by HPAEC analysis.

The sulfatase, galactosidases, and fucosidases used above are **stereospecific**, further confirming the nature of the substrate. The reviewer should/will be aware of this as their activities are published; https://www.nature.com/articles/s41564-019-0466-x, https://www.nature.com/articles/s41467-025-58660-2, and https://doi.org/10.1042/EBC20220158.

Finally, these sulfated Lewis substrates are chemically synthesised and commercially available (from Dextra) with the presence of the 6S-Lewis structures of each substrate confirmed in a QC report. We would be willing to ask Dextra to supply additional copies of these QC reports.

2) The lack of reproducibility for colonic mucin. I agree that this is a difficult procedure, but how can the results from one extraction be considered representative and how is anyone supposed to reproduce the work?

RESPONSE: We have robustly benchmarked our work by providing mass spectrometry, NMR, and glycoprotein gel staining of the gpcMO material. For those wishing to reproduce our gpcMO substrate, we have provided full details: they can vary the reaction conditions until material is obtained that matches these bench marks. Details of the entire procedure, and our subsequent analysis, has been provided in the methods and results. This provides complete transparency to allow replication by other groups.

Finally, the extractions are from 10 porcine distal colons ~1 meter in length but this has little effect on the generation of gpcMOs/cMOs. This is done *after* extraction and is a separate process, as detailed in the methods.

3) I am not sure how you have concluded increased lag phases for the 0490-491 (line 365). The lags are comparable to the monosaccharide control. They also reach the same OD, suggesting the same amount of substrate can be accessed. An increased lag suggest a response delay to substrate (both mucin and monosaccharides), not a substrate access issue. I suggest removing the conclusion of any phenotype.

RESPONSE: The reviewer has succinctly defined the phenotype; the 0490-491 mutant has a defect (increased lag phase) on all substrates tested compared to wildtype. We have not suggested any role of substrate access and stated the effect

was observed across all substrates thus not mucin specific. We have placed the excerpt from the manuscript below:

'When grown on sPGM and gpcMOs, most mutants displayed no growth differences to wildtype except amuc0490-491::tn (which had an increased lag phase on both sPGM and gpcMOs) and amuc1182::tn (which had an increase in lag phase on sPGM). The Tn insertion in amuc0490-491::tn spans both genes and the defect observed here is likely to be linked to amuc0490, since it is observed across all substrates tested.'

We have revised this to avoid confusion:

'On sPGM and gpcMOs most mutants displayed no growth differences to wildtype except *amuc0490-491*::tn and *amuc1182*::tn. The mutant *amuc1182*::tn had an increased lag phase only on sPGM, while the *amuc0490-491*::tn mutant show an increased lag on all substrates including GlcNAc. The Tn insertion in *amuc0490-491*::tn spans both genes and the defect observed here is likely linked to amuc0490 (a putative inner membrane protein of unknown function) and is a global defect not specific to mucin or the sulfatase Amuc0490$^{3S\text{-}Gal/GalNAc}$.'

Furthermore, some of your conclusions do not consider the full picture and from my perspective go too far or are incorrect. For example:
Line 146 - I would say that the growth profiles on the different substrates suggest (not reveal) substrate preferences - you have not directly confirmed this in your study.

RESPONSE: We strongly disagree with this comment. Growth profiles are the ultimate test of whether a bacterial strain prefers one substrate over another, and can be more informative than the sum of various reductionist enzyme assays. While growth experiments are simple, they reveal important biological information in the context of the whole cellular system.

Line 160 – an increase in the expression of the 0627 gene has been observed previously on PGM in the Desai, Ottman, Davey, and Bakshani papers, so this conclusion is not convincing. It is difficult to accept your conclusion if these publications are taken into consideration.

RESPONSE: We have removed this point as it is a minor contribution to the paper which primarily focuses on how *A. muciniphila* grows on, and deploys carbohydrate sulfatases, to access colonic mucins. We accept the Amuc_0627 up regulation on gastric mucins is significant.

The revised sentence reads as such:

'When comparing gpcMOs to sPGM samples only three sulfatases (Amuc0491, Amuc1655 and Amuc1182) are more upregulated (**Figure 1f and g**). Overall, the different gastric and colonic mucin substrates did not lead to large differences in sulfatase expression **(Extended data figure 1d and Supplementary dataset 3).'**

Line 57 and 377 – I do not think your results support this conclusion directly

RESPONSE: We presume this is in response to the growth phenotypes in figure 1 showing *A. muciniphila* has a preference for glycopeptide/proteins of a specific size, and is a repetition of the same point above about microbial growth curves. **The results are clear and accepted by the other 3 expert reviewers.** The confusion of the reviewer likely relates to their misinterpretation of microbial growth data as already detailed above.

Line 385 – there is no evidence that the GH16 enzymes only fit a cross-feeding role. The Davey paper shows a decrease in growth for the 2108 Tn mutant, for example. Akker must be able to grow on oligos to an extent as you show with your own data - Figure 1b.

RESPONSE: We note this is part of our discussion and not the results. *A. muciniphila* **does not** grow on highly sulfated colonic mucin oligosaccharides as confirmed by our data and Luis et al (https://www.nature.com/articles/s41586-021-03967-5#Sec28). Our data shows it grows poorly on low sulfation gastric mucin oligosaccharides. *A. muciniphila* has cell surface GH16 enzymes that produce oligosaccharides from sulfated mucins (https://www.nature.com/articles/s41467-020-17847-5) and *A. muciniphila* cannot use these, therefore, we suggest that they may be used by other organisms. This does not seem a large intellectual leap. Amuc_2106 is predicted to be a **periplasmic** enzyme which renders this reviewers' comparison invalid in the context of cell surface activities.

Line 50 - A. muciniphilainiphila typo
RESPONSE: This has been corrected

Line 52 – what rare adaptations?
RESPONSE: These are mutations that occur infrequently and afford the sulfatases tailored activities. This analysis was conducted by examining ~100s of sequences, adjusted for taxonomic bias, across the sulfatase subfamilies and looking at the frequency of specific amino acids at positions of interest; thus identifying the A. muciniphila sulfatases have rare adaptations in the active site explaining there specificity to disaccharide substrates.

Figure 6 – the figure has changed but the legend was not updated. I am not convinced that both constructs were pulled down – it's mostly in the supernatant and there are
thin lines for the wash an pellet?

RESPONSE: We were remiss in not updating the legend, this has now been done. However, it is common to have unbound protein in the wash because an excess of protein (10 μM) is used to saturate the mucin. The key is that both Amuc0953^{P395} and Amuc0953^{P490} are pulled down in the pellet, while BSA is not (Figure 6a (i)). A ten-fold enrichment of the pellet shows that Amuc0953^{P395} and Amuc0953^{P490} are enriched, and BSA is not (Figure 6a (ii)). Furthermore, this interaction is only observed with PCM and not the control polysaccharide, cellulose. Finally, western blots were run with an anti-His tag antibody to confirm the presence or absence of the recombinant protein.

Furthermore, with the new data in b, the conclusions about binding are unclear as the 0953 is smaller then the BSA bands – is there another protein you could use as a comparison?

RESPONSE: This appears to be a misunderstanding of the nature of the assay; only the level of retardation matters relative to the control protein BSA rather than the absolute mass of the proteins. Amuc0953^{P395} is clearly retarded by the colonic and not the gastric substrate. BSA is the gold standard control for these assays, please see the following papers:

https://pubmed.ncbi.nlm.nih.gov/27298375/
https://www.sciencedirect.com/science/article/pii/S0014579315005888
https://www.nature.com/articles/srep19392
https://www.nature.com/articles/s41467-022-28310-y.

Reviewer #2 (Remarks to the Author):

The authors kindly answered all my questions/suggestions satisfactorily.

RESPONSE: We thank the reviewer for their support of the manuscript.

Reviewer #3 (Remarks to the Author):

I reviewed the previous version of this manuscript, and returned comments regarding the need for more evidence to support mucin substrate growth preferences. I also had comments regarding the overall clarity of the manuscript (also raised by Rev1).

This new version appears to be improved in terms of clarity. There are new gel affinity data regarding the interaction of Amuc0953 with gpcMO in response to one of my queries, and my other questions are also dealt with.

I am broadly happy for this version to go ahead, with some (minor) comments.

RESPONSE: We thank the reviewer for their support of the manuscript.

- Regarding the use of thermal shifts to infer binding affinities (originally Fig 2c, now Ext Fig 3b). I stand by my original assertion that this is not a solid interpretation of the data. To be clear, my specific issue is with the claim of measuring affinity.

The authors are correct that thermal shift measures stabilization of the protein by the ligand, and that this is indicative of an interaction. However, there appears to be conflation between the magnitude of the stabilization effect, and affinity of the ligand that is creating the stabilization (i.e. efficacy vs potency, to borrow terms from pharmacology).

I agree that larger stabilization magnitudes are likely to correlate with a better ligand affinities, as there is more binding/stabilization energy, but I do not believe this relationship is ironclad (may be ligand entropic/solvation effects on ligand that affect binding affinity, without necessarily stabilizing protein etc etc).

The thermal shift data in Ext Fig 3b shows 6S-Gal stabilize Amuc0121 with higher magnitude, and from 0.1 mM ligand. However, since saturating ligand concentrations where stabilization plateaus were never reached, it is difficult to be certain about affinity.

Better phrasing would just be that Amuc0121 shows lower thermal shift upon 6S-LewisA binding, indicative of a weaker, less stabilizing interaction.

RESPONSE: We agree with the reviewer and realise we misunderstood their initial assertion and have adjusted the text as suggested.

'although it shows lower thermal shift upon 6S-LewisA binding compared to 6S-Gal, indicative of a weaker, less stabilizing interaction'

Some other issues that will probably come out in editing:
- Fig 6 – caption doesn't match figure
RESPONSE: This has been corrected

- Abstract – A. muciniphilainiphila
RESPONSE: This has been corrected

- L158 - gpcMO samples did show that the mucin glycoprotease Amuc0627 was highly upregulated. The text refers to Fig g, which is a volcano plot. However, it is not shown in this plot where Amuc0627 is, and only GHs and sulfatases are highlighted. Annotation should be made more clearly in the figure (and if applicable, related volcano plots in Ext Fig 1)

Reviewer #4 (Remarks to the Author):

The authors have convincingly addressed all of my previous comments. Only minor typographical issues remain in this version:

RESPONSE: We thank the reviewer for their support of the manuscript.

- Line 50: "A. muciniphilainiphila"?
RESPONSE: This has been corrected

Line 56: "monosaccharode"? Perhaps several of the authors, including the two corresponding authors, could proofread the text with more care.

RESPONSE: This has been corrected

- Lines 96-100: This sentence is grammatically challenging and unclear.
RESPONSE: This sentence has been broken into two sentences and rewritten as below:

'To date most studies of *A. muciniphila* have focused on characterising its glycoside hydrolases (GHs), using gastric mucins which are low in sulfation. In gastric mucin only the

*O*6 position of *N*-acetyl-D-glucosamine (GlcNAc) is known to be sulfated, whilst sulfation of colonic mucin is known to occur on the *O*3, *O*4, and *O*6 of D-Galactose (Gal), as well as the *O*6 of GlcNAc[11,33]'

- Lines 95-96, with reference to my comment 2 on the previous version: In light of the authors' response, it would be most appropriate to insert a citation to the SulfAtlas database (current ref. 15) immediately after "...encodes 10 S1 carbohydrate sulfatases, classified into 6 subfamilies,"

RESPONSE: reference has been added

- Line 100: "has shown that for"?

RESPONSE: the 'for' has been deleted the sentence now reads as:

'Recent data, however, has shown that heavily sulfated colonic mucin glycoprotein, and not free colonic mucin oligosaccharides (cMOs), are needed to support growth[34,35].'

- Line 110: Change to "...to reveal that glycoprotein fragments,"

RESPONSE: sentence has been changed as requested.

- Line 113: Change to "sulfatase-linked, mucin-binding" All similar noun-verb conjugates used as adjectives should be similarly hyphenated throughout the text, to improve readability.

RESPONSE: These have been amended.

End of comments.

We thank reviewer 3 for their helpful and fair review of our work and acting as a mediator. We have responded to reviewers 3 comments in **green** below their comments in **red** in the hope this keeps the document clear.

Reviewers Comments:

Reviewer #3 (Remarks to the Author):

I have been asked to mediate the exchange between the authors and Reviewer 1, following their critical review of the previous version of the manuscript.

In summary, I am mostly in agreement with the authors, and I feel Rev1's criticisms are adequately addressed in most cases. However, there are some technical issues on quality where I feel Rev1 is justified in their criticism.

Point by point

- *Rev1*
- *Authors response*
- *My response*

1) *The TLC in Fig2b – this is the core evidence of 6'S-LewisA/X activity, which is a really important element to the novelty. In my opinion the assays and TLCs need re-running. The two furthest left spots for the top TLC (control and +0121) - where have the blue and pink spots gone in the +0121 sample? How does this weird result relate to your response about the substrate being commercially acquired? They look like different substrates on that TLC. The bottom TLC is also not a clean result.*

RESPONSE: *The activity of Amuc0121 on 6S-Lewis antigens is supported by TLC, HPAEC and TSA. We do not rely solely on this TLC result; the substrate used in these assays was also co-crystallised with the protein revealing the atomic details of the protein-substrate interaction. The comment 'they look like different substrates' is curious as when an enzyme acts on a substrate it produces a product and this must look different from the start material i.e. spots for substrate disappear and new spots relating to product appear. The pink and blue spots are the substrate.*

Focusing more on the 6S-LewisA; it is true the control has three spots, two of which (the blue and pink) respond to Amuc0121 to produce a single spot, meaning that they produce the same product upon removal of the O6 linked sulfate group. The 6S-LewisA substrate is unresponsive to the fucosidase BT4136 and galactosidase BT4241 which is consistent with them being active on LewisA but not 6S-LewisA. Upon addition of Amuc0121, and both BT4136 and BT4241, the products of galactose, fucose, and N-acetylglucosamine are produced, consistent with the removal of O6 sulfation thereby allowing access to the LewisA substrate. The data are corroborated with HPAEC analysis.

The 6S-LewisX TLC is a cleaner result and clearly shows the same trend as the 6S-LewisA data; there is a definitive shift upon the addition of Amuc0121, and Amuc0121 allows access of the galactosidase and fucosidase to degrade the substrate. Again, these data are backed up by HPAEC analysis.

*The sulfatase, galactosidases, and fucosidases used above are **stereospecific**, further confirming the nature of the substrate. The reviewer should/will be aware of this as their activities are published; https://www.nature.com/articles/s41564-019-0466-x, https://www.nature.com/articles/s41467-025-58660-2, and https://doi.org/10.1042/EBC20220158.*

Finally, these sulfated Lewis substrates are chemically synthesised and commercially available (from Dextra) with the presence of the 6S-Lewis structures of each substrate confirmed in a QC report. We would be willing to ask Dextra to supply additional copies of these QC reports.

I side with Rev1 here. The TLCs look strange, or at the very least are poorly explained.

- Fig2b top panel (6'S-Lewis A) - it is not explained why the control lane has 3 spots, when it is a (presumably) homogenous pure compound from Dextra. Without further context, I would expect pure, stable compounds to only give a single spot on TLC - I also see no reason why 6'S-Lewis A should **not** be stable on silica. The result is made more problematic as the top spot appears to be the same Rf and colour as GlcNAc only, suggesting contamination. If this discrepancy can be explained, then I think the rest of the TLC makes sense.

- I also agree with Rev1 that the TLC quality is poor. For example, the +Amuc0121/BT4136/BT4142 lane where we would expect to see digest products, the spot corresponding to Fuc is basically not visible. I suggest more material needs to be loaded, or another stain used. There are also issues with annotation - is BT4142 the b1,3 galactosidase, or BT4243 as per the figure caption text?
- Fig2b bottom panel (6'S-Lewis X) - suffers from the same problem as the top panel, but the quality looks even worse. Spots in the +Amuc0121/BT4136/BT0461 lane are basically not visible.

Action points
- Issues with TLC spots should ideally be resolved or clarified. QC reports from manufacturers would help, or else the authors should run their own LCMS or NMR analyses to confirm the substrates are pure.
- I appreciate the authors' response that there are multiple pieces of evidence for the Amuc0121 digestion profile. In particular, HPAEC data seems more convincing than the TLC. Why is this not in the main text?

We have purchased two new batches of the 6'S-LewisA and 6'S-LewisX from Dextra and reran the TLC and HPAEC experiments. We have now also included TSA data for 6'S-LewisX.

The 6'S-LewisA TLC is now much cleaner (now extended data figure 3a) with only a single spot in the control. We have however moved the HPAEC data into the main text (Figure 2b), as suggested by reviewer 3, as this more clearly shows the presence of all three constituent monosaccharides. We would like to stress that the HPAEC and the TLC data are the same samples/reactions ran by two different techniques.

The new batch of 6'S-LewisX, although cleaner, still has contaminating bands visible on the TLC. However, both the TLC and HPAEC show that only when the sulfatase Amuc0121 is present are the fucosidase and galactosidase able to cleave the 6'S-LewisX structure to its constituent monosaccharides of Gal, GlcNAc, and Fuc. Although contaminating Fuc obscures the released Fuc in the HPAEC trace one can only get GlcNAc if the Fucosidase, BT4136, has acted and this only occurs after treatment with Amuc0121. Qualitatively there appears weaker activity against 6'S-LewisX by HPAEC and TLC (compared to 6'S-LewisA) but the TSA assays, however, show an interaction comparable with 6S-Gal.

We hope collectively these new experiments, with new material, will help alleviate the concerns reviewer 3 may still have

2) The lack of reproducibility for colonic mucin. I agree that this is a difficult procedure, but how can the results from one extraction be considered representative and how is anyone supposed to reproduce the work?

RESPONSE: *We have robustly benchmarked our work by providing mass spectrometry, NMR, and glycoprotein gel staining of the gpcMO material. For those wishing to reproduce our gpcMO substrate, we have provided full details: they can vary the reaction conditions until material is obtained that matches these bench marks. Details of the entire procedure, and our subsequent analysis, has been provided in the methods and results. This provides complete transparency to allow replication by other groups.*

Finally, the extractions are from 10 porcine distal colons ~1 meter in length but this has little effect on the generation of gpcMOs/cMOs. This is done after extraction and is a separate process, as detailed in the methods.

Tricky, but I just about side with the authors here. I see the gpcMO result as an initial

point of serendipity that drove subsequent analysis of the sulfatases, which is the main story of the paper. As the authors state, the gpcMO extract is benchmarked by several analytical techniques.

We are glad the reviewer has sided with us on this point. We worked hard to benchmark the work using several analytical techniques.

3) I am not sure how you have concluded increased lag phases for the 0490-491 (line 365). The lags are comparable to the monosaccharide control. They also reach the same OD, suggesting the same amount of substrate can be accessed. An increased lag suggest a response delay to substrate (both mucin and monosaccharides), not a substrate access issue. I suggest removing the conclusion of any phenotype.

RESPONSE: *The reviewer has succinctly defined the phenotype; the 0490-491 mutant has a defect (increased lag phase) on all substrates tested compared to wildtype. We have not suggested any role of substrate access and stated the effect was observed across all substrates thus not mucin specific. We have placed the excerpt from the manuscript below:*

'When grown on sPGM and gpcMOs, most mutants displayed no growth differences to wildtype except amuc0490-491::tn (which had an increased lag phase on both sPGM and gpcMOs) and amuc1182::tn (which had an increase in lag phase on sPGM). The Tn insertion in amuc0490-491::tn spans both genes and the defect observed here is likely to be linked to amuc0490, since it is observed across all substrates tested.'

We have revised this to avoid confusion:

'On sPGM and gpcMOs most mutants displayed no growth differences to wildtype except amuc0490-491::tn and amuc1182::tn. The mutant amuc1182::tn had an increased lag phase only on sPGM, while the amuc0490-491::tn mutant show an increased lag on all substrates including GlcNAc. The Tn insertion in amuc0490-491::tn spans both genes and the defect observed here is likely linked to amuc0490 (a putative inner membrane protein of unknown function) and is a global defect not specific to mucin or the sulfatase Amuc0490$^{3S\text{-}Gal/GalNAc}$.'

I side with the authors. I think they are sufficiently clear about the phenotype.

We are glad we were clear about the phenotype.

Furthermore, some of your conclusions do not consider the full picture and from my perspective go too far or are incorrect. For example:
Line 146 - I would say that the growth profiles on the different substrates suggest (not reveal) substrate preferences - you have not directly confirmed this in your study.

RESPONSE: *We strongly disagree with this comment. Growth profiles are the ultimate test of whether a bacterial strain prefers one substrate over another, and can be more informative than the sum of various reductionist enzyme assays. While growth experiments are simple, they reveal important biological information in the context of the whole cellular system.*

Side with authors.

We thank the reviewer for their insight.

Line 57 and 377 – I do not think your results support this conclusion directly
RESPONSE: *We presume this is in response to the growth phenotypes in figure 1 showing A. muciniphila has a preference for glycopeptide/proteins of a specific size, and is a repetition of the same point above about microbial growth curves.* ***The results are clear and accepted by the other 3 expert reviewers.*** *The confusion of the reviewer likely relates to their misinterpretation of microbial growth data as already detailed above.*

N/A – related to the above point

Line 385 – there is no evidence that the GH16 enzymes only fit a cross-feeding role. The Davey paper shows a decrease in growth for the 2108 Tn mutant, for example. Akker must be able to grow on oligos to an extent as you show with your own data - Figure 1b.

RESPONSE: *We note this is part of our discussion and not the results. A. muciniphila* ***does not*** *grow on highly sulfated colonic mucin oligosaccharides as confirmed by our data and Luis et al (https://www.nature.com/articles/s41586-021-03967-5#Sec28). Our data shows it grows poorly on low sulfation gastric mucin oligosaccharides. A. muciniphila has cell surface GH16 enzymes that produce oligosaccharides from sulfated mucins (https://www.nature.com/articles/s41467-020-17847-5) and A. muciniphila cannot use these, therefore, we suggest that they may be used by other organisms. This does not seem a large intellectual leap. Amuc_2106 is predicted to be a* ***periplasmic*** *enzyme which renders this reviewers' comparison invalid in the context of cell surface activities.*

This is not my core expertise, but I feel like this is a reasonable thing to mention in the discussion.

In the interest of reducing the word count we have removed this from the discussion.

Line 50 - A. muciniphilainiphila typo
RESPONSE: *This has been corrected*

N/A – issue has been dealt with

Line 52 – what rare adaptations?
RESPONSE: *These are mutations that occur infrequently and afford the sulfatases tailored activities. This analysis was conducted by examining ~100s of sequences, adjusted for taxonomic bias, across the sulfatase subfamilies and looking at the frequency of specific amino acids at positions of interest; thus identifying the A. muciniphila sulfatases have rare adaptations in the active site explaining there specificity to disaccharide substrates.*

N/A – issue has been dealt with

Figure 6 – the figure has changed but the legend was not updated. I am not convinced that both constructs were pulled down – it's mostly in the supernatant and there are thin lines for the wash an pellet?

RESPONSE: *We were remiss in not updating the legend, this has now been done. However, it is common to have unbound protein in the wash because an excess of protein (10 μM) is used to saturate the mucin. The key is that both Amuc0953^{P395} and Amuc0953^{P490} are pulled down in the pellet, while BSA is not (Figure 6a (i)). A ten-fold enrichment of the pellet shows that Amuc0953^{P395} and Amuc0953^{P490} are enriched, and BSA is not (Figure 6a (ii)). Furthermore, this interaction is only observed with PCM and not the control polysaccharide, cellulose. Finally, western blots were run with an anti-His tag antibody to confirm the presence or absence of the recombinant protein.*

I side with the authors. The pulldown is not designed to be quantitative, so leftovers in the supernatant are not unexpected. The observation that the Amus0953 constructs are pulled down by the PCM, not the BSA, and not in the cellulose controls, is consistent with the claimed interpretations

We thank the reviewer for their insight.

Furthermore, with the new data in b, the conclusions about binding are unclear as the 0953 is smaller then the BSA bands – is there another protein you could use as a comparison?

RESPONSE: *This appears to be a misunderstanding of the nature of the assay; only the level of retardation matters relative to the control protein BSA rather than the absolute mass of the proteins. Amuc0953^{P395} is clearly retarded by the colonic and not the gastric substrate. BSA is the gold standard control for these assays, please see the following papers:*

https://pubmed.ncbi.nlm.nih.gov/27298375/
https://www.sciencedirect.com/science/article/pii/S0014579315005888
https://www.nature.com/articles/srep19392
https://www.nature.com/articles/s41467-022-28310-y.

Overall I side with the authors, although the figure could be clearer. Annotation would be useful, especially e.g. MW markers that each gel could be benchmarked to. The fact that BSA itself looks like it has run differently on the gpcMO gel likely adds to confusion.

Unfortunately, the technique does not lend itself to markers. It is only the retardation relative to a control protein that can be effectively observed. The issue is that these are native gels with and without a glycan embedded in the gel. The charge of the protein is imparted by its pI. One could have five proteins of the exact same molecular mass but they will run differently due to their different pIs and the different charges imparted. For some proteins where the pI is very high (this is rare in our experience) they will be positively charged and run out of the gel towards the cathode, so would not work with the technique. We have added M, D, and T to the side of the gel to indicate the monomeric, dimeric, and tetrameric bands of BSA. These would be ~66, 122, and 244 kDa, respectively and included this information in the figure legend.

Reviewer #3:
Remarks to the Author:
My two main criticisms last time were to do with the quality of the TLC analysis for 6'S-Sia-LewisA/X digestion (Figure 2b), and annotation needed for the gel shift assay (Figure 6b).
We are happy to have satisfied this point.

TLC analysis - The rerun TLCs with new/cleaner substrate look ok, especially for Lewis A. The TLC for Lewis X still looks slightly weak, but the main findings are also confirmed by HPAEC, so as a package of results the conclusions seem sound. The HPAEC for Lewis A digest has been moved to the main text, which is good as these data are higher quality than the TLC.
We are happy to have satisfied this point.

The authors however still need to fix their annotation in the figure caption. For Figure 2d, the caption mentions BT4241, but the figure has BT4142. Which is correct? The caption also still mentions BT0461, despite the fact the relevant data have now been moved to extended data.
This has now been corrected in the figure as the legend is correct.

Annotation for gel shift assay - this is fine for me now.
We are happy to have satisfied this point.
